# Ligand functionalization as a deactivation pathway in a *fac*-Ir(ppy)_3_-mediated radical addition†
†Electronic supplementary information (ESI) available. See DOI: 10.1039/c4sc03064h



**DOI:** 10.1039/c4sc03064h

**Published:** 2014-10-20

**Authors:** James J. Devery III, James J. Douglas, John D. Nguyen, Kevin P. Cole, Robert A. Flowers II, Corey R. J. Stephenson

**Affiliations:** a Department of Chemistry , University of Michigan , Ann Arbor , USA . Email: crjsteph@umich.edu ; Fax: +1-734-647-4865 ; Tel: +1-734-763-8283; b Small Molecule Design and Development , Eli Lilly and Company , Indianapolis , USA; c Department of Chemistry , Lehigh University , Bethlehem , USA . Email: rof2@lehigh.edu ; Fax: +1-610-758-6538 ; Tel: +1-610-758-4048

## Abstract

Knowledge of the kinetic behavior of catalysts under synthetically relevant conditions is vital for the efficient use of compounds that mediate important transformations regardless of their composition or driving force. In particular, these data are of great importance to add perspective to the growing number of applications of photoactive transition metal complexes. Here we present kinetic, synthetic, and spectroscopic evidence of the mechanistic behavior of *fac*-Ir(ppy)_3_ in a visible light-mediated radical addition to 3-methylindole, demonstrating the instability of *fac*-Ir(ppy)_3_ under these conditions. During the reaction, rapid *in situ* functionalization of the photocatalyst occurs, eventually leading to deactivation. These findings demonstrate a conceivable deactivation process for catalytic single electron reactions in the presence of radicophilic ligands. Attempts to inhibit photocatalyst deactivation through structural modification provide further insight into catalyst selection for a given system of interest.

## Abstract 2

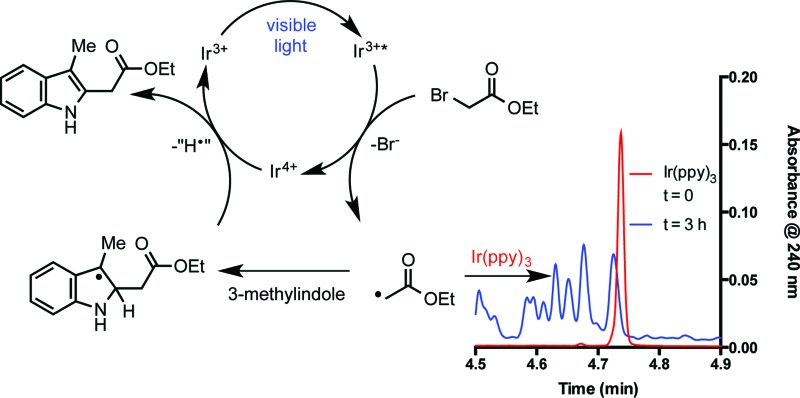
Kinetic, synthetic, and spectroscopic evidence demonstrates the instability of *fac*-Ir(ppy)_3_ under visible light-mediated photoredox conditions resulting from *in situ* functionalization.

## Introduction

Visible light-mediated photoredox catalysis is a rapidly developing field, encompassing methods for the activation of organic molecules *via* the formation of ions, radicals, and radical ions.^1^ These transformations utilize either photoactive transition metal-centered complexes or organic dyes to mediate reactions ranging from simple reductions and oxidations to complex domino processes.^2^ Historically, metal-centered photoredox catalysts have been employed in inorganic and materials chemistry, facilitating chemical methods in hydrogen and oxygen production,^3^ as well as the generation of methane *via* multiple reductions of CO_2_.^4^ Furthermore, new materials have been developed for photovoltaics,^5^ optical sensing,^6^ OLEDs,^7^ and photodynamic therapy.^8^ These diverse applications have been accomplished through exhaustive analysis of the thermodynamic properties of these complexes, characterization of their ground and excited states, photochemical transitions, and the electrochemistry involved in the modification of the oxidation state of the metal center.^9^ This array of data, for an ever-increasing number of complexes, allows the synthetic chemist to select a catalyst with the specific electrochemical properties required for a proposed transformation. Despite this wealth of information, little is known about the kinetic properties of these complexes in organic reactions beyond classical Stern–Volmer analysis.^10^

Tris[2-phenylpyridinato-C^2^,*N*]iridium(iii) (*fac*-Ir(ppy)_3_, **1**)^11^ has recently been utilized in a variety of organic processes ranging from benzylation,^12^ α-amino arylation,^13^ intramolecular cyclization,^14^ polymerization,^15^ addition to styrenes,^16^ decarboxylative arylations,^17^ β-arylation of carbonyls,^18^ to decarboxylative trifluoromethylations.^19^ Our group applied **1** to the reduction of unactivated alkyl, alkenyl, and aryl iodides.^20^ The reducing power of this complex in its excited state (Ir^iv/iii^* = –1.73 V *vs.* SCE) is greater than the reductively quenched state of tris(bipyridine)ruthenium(ii) chloride (Ru(bpy)_3_Cl_2_, Ru^ii/i^ = –1.33 V *vs.* SCE). After single electron reduction of the substrate, the resulting quenched state (Ir^iv/iii^ = +0.77 V *vs.* SCE) performs mild oxidations. As a result, we applied **1** to the redox neutral coupling of 3-methylindole (**2**) and ethyl bromoacetate (**3**) to form alkylated indole **4** in excellent yield (eqn (1)). This process features many of the standard mechanistic steps present in radical-based photoredox processes: photoexcitation of a transition metal complex to generate a long-lived excited state, bimolecular quenching of the excited state *via* single electron transfer, and formation of a carbon-centered radical capable of further reactivity.^21^ Because this system bears many of the properties of a typical visible light-mediated process, it makes an excellent model for studying the kinetic properties of a photocatalyzed organic reaction.1
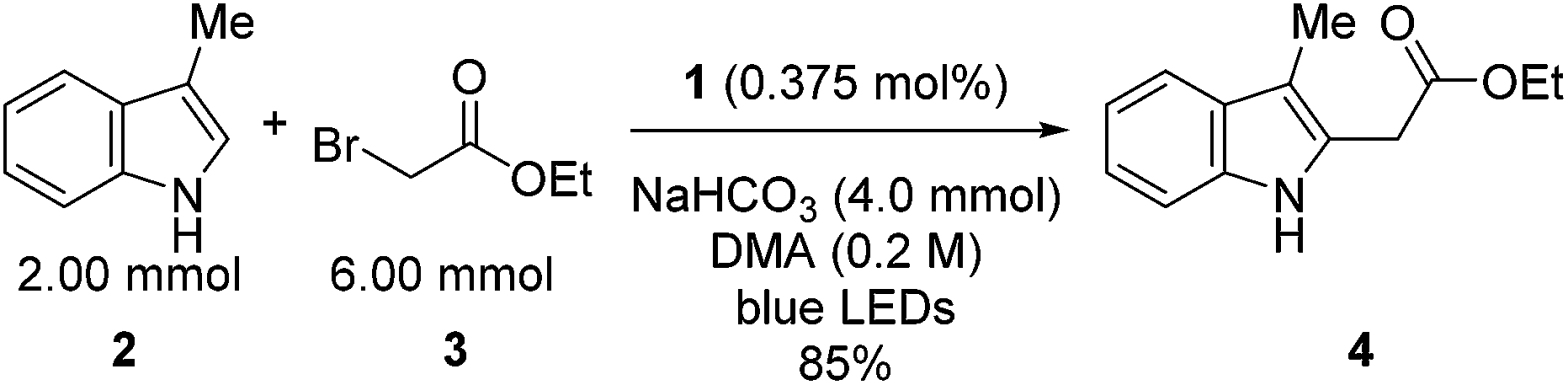



## Results and discussion

**Fig. 1:**
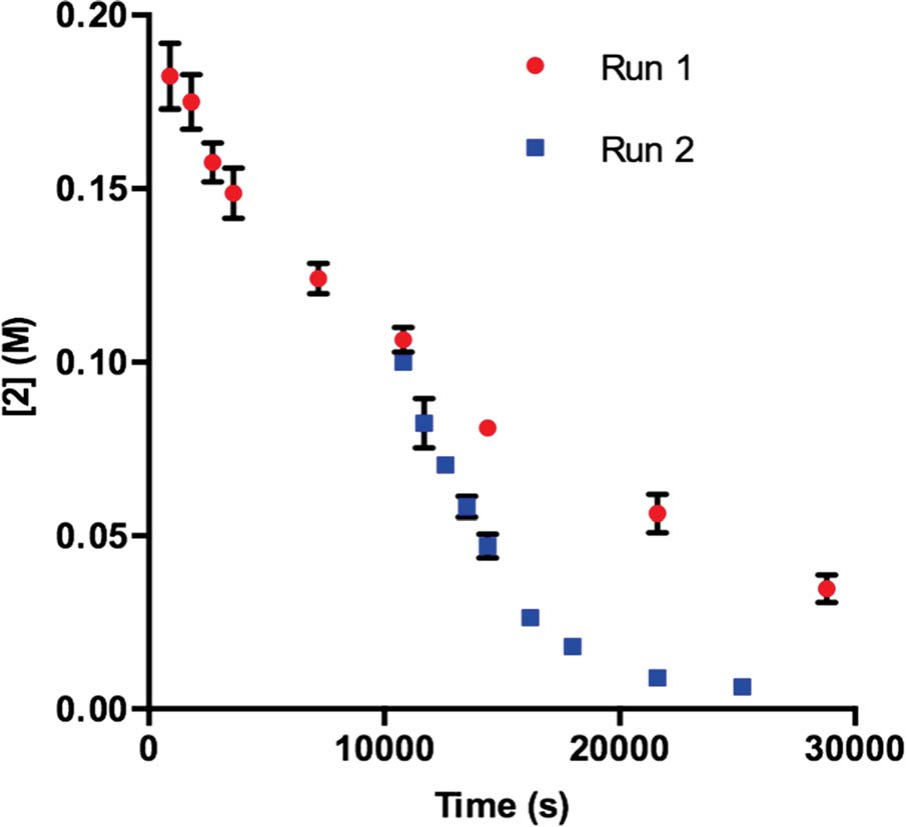
Profile of Run 1 as [**2**] *vs.* time and the time-adjusted profile of Run 2 as [**2**] *vs.* adjusted time.^24^

Our initial efforts to elucidate the mechanistic behavior of **1** in the model reaction began with observation of the stability of the catalyst under synthetically relevant conditions.^22,23^ Using the reaction defined in eqn (1), we designated indole **2** as the limiting substrate with all other components except **1** added in excess and extracted kinetic information by monitoring [**2**] *via* reversed-phase ultra performance liquid chromatography (UPLC) coupled with a photo-diode array (PDA) detector. All kinetic data were determined from the mean of three different reactions with respect to an internal standard and plotted as [**2**] as a function of time (Fig. 1, Run 1).^24^

Under ideal conditions, when the reaction reaches the first half-life, with respect to [**2**] (time = 10^4^ s, Fig. 1, Run 1), equivalent amounts of **3** and NaHCO_3_ are consumed, and, most importantly, the total **1** should remain constant. A reaction initiated under these same conditions should follow the concentration profile and provide a graphical overlay. In the absence of the reaction products, we initiated a transformation at the first half-life of Run 1, in an attempt to duplicate the reaction composition at 10^4^ s. When these concentration data are plotted *vs.* time, where time_0_ = 10^4^ s and subsequent concentrations are time-adjusted accordingly, Run 2 does not overlay with Run 1 (Fig. 1).^24^ This graphical observation manifests as a result of the [**2**] decreasing at a much higher rate in Run 2 under what should be identical conditions. These data are consistent with two mechanistic possibilities: (1) total **1** is not constant due to deactivation or, (2) product indole **4** or other reaction byproducts inhibit the turnover of the catalyst.

**Fig. 2:**
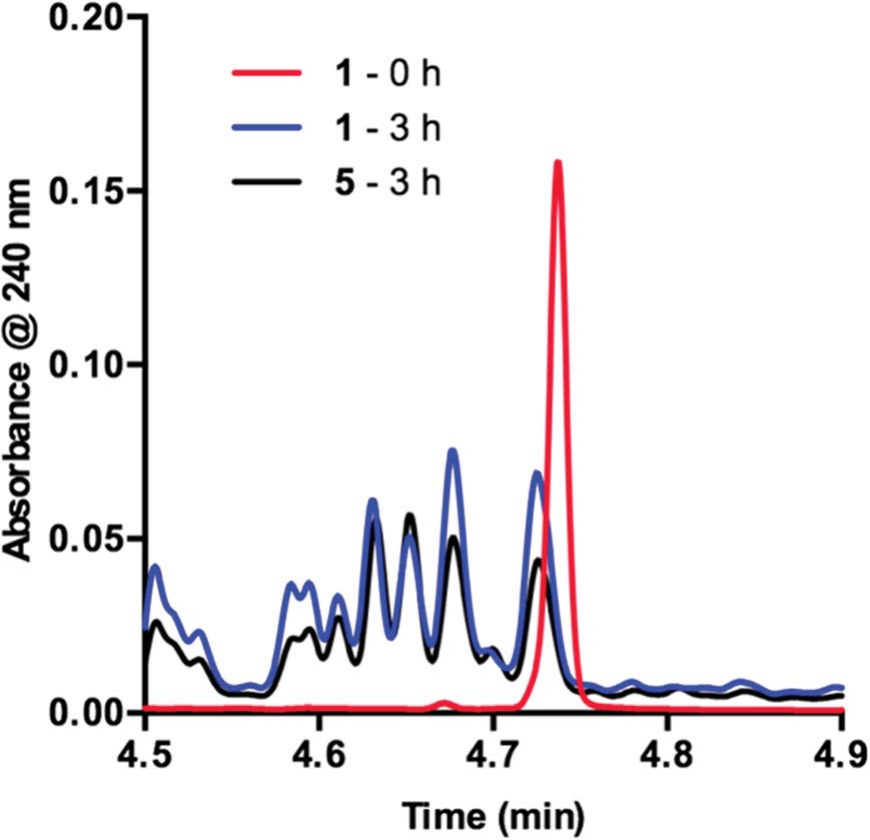
UPLC trace of **1** at time = 0 h and time = 3 h. Signal observed at 240 nm.

To obtain more insight into this result, we examined the UPLC traces generated at each of the time points throughout the course of the reaction. Prior to irradiation with blue LEDs, a clearly defined peak corresponding to **1** is present at 4.74 min (Fig. 2, **1** – 0 h). As the reaction proceeds under irradiation, this peak diminishes and is replaced by a variety of signals at shorter retention times, suggestive of species with increased polarity (Fig. 2, **1** – 3 h). These data are consistent with deactivation of **1**.

In order to determine the cause of this inhibitory process, we irradiated the catalyst under reaction conditions in the absence of **2** [eqn (2)]. This system led to complete conversion of **1** to an intractable mixture of products after 2 h. Addition of **2** to this mixture of complexes and then irradiation with light yielded 1.5% conversion of substrate in the first 2 h with no further change detected after 24 h. Under normal reaction conditions, 38 ± 2% conversion of **2** occurs in the initial 2 h of the reaction. Reduction in the amount of **3** with respect to **1** yielded one characterizable product, monoalkylated complex **5**.^25^ To determine if this complex is a deactivated form of the catalyst, we substituted **5** into the model system to act as the catalyst. To our surprise, the reaction proceeded efficiently. Kinetic analysis of this new complex showed complete conversion of **2**, as well as catalyst deactivation. Additionally, comparison of the UPLC traces of **1**- and **5**-mediated reactions at 3 h displays higher polarity peaks with identical retentions times (Fig. 2, **5** – 3 h). These data suggest that, while capable of catalyzing the reaction, complex **5** may be an intermediate formed in the process of deactivation. To gain further insight into the degree of functionalization that can occur, we subjected the intractable mixture of complexes to mass spectrometric analysis. These data displayed masses corresponding to mono-, di-, tri-, tetra-, and pentaalkylations of **1**.^26^
2
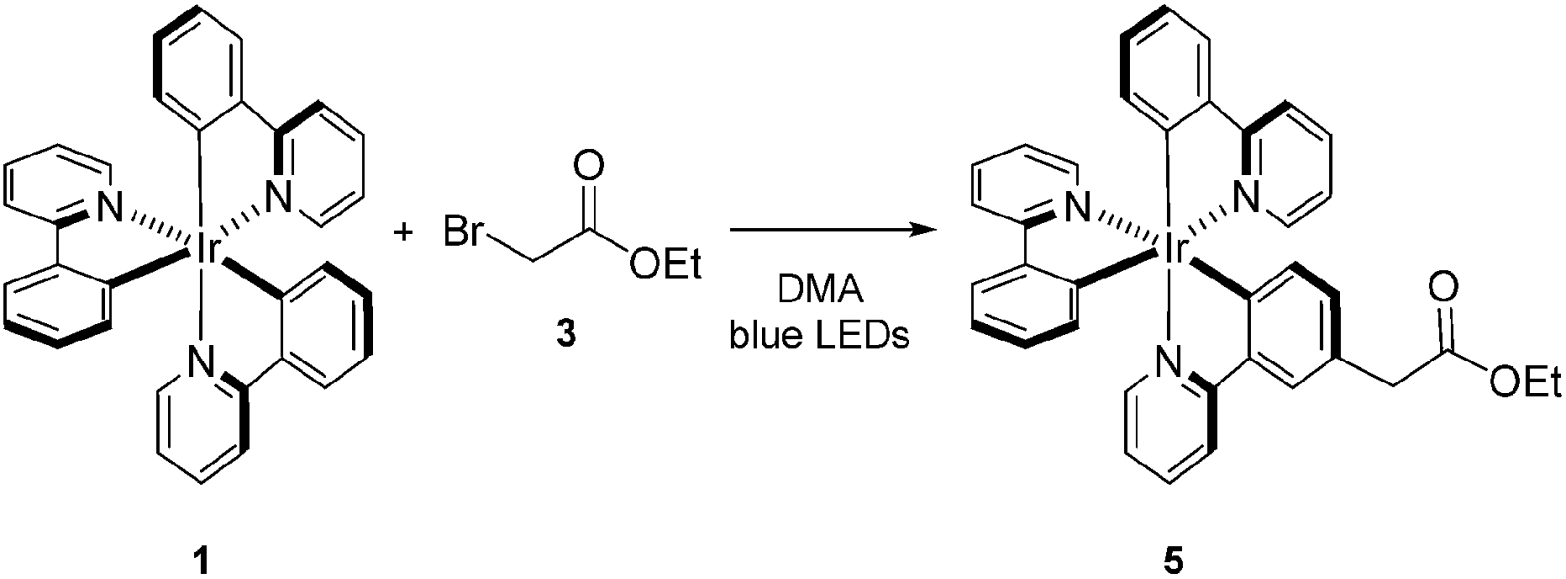



**Fig. 3:**
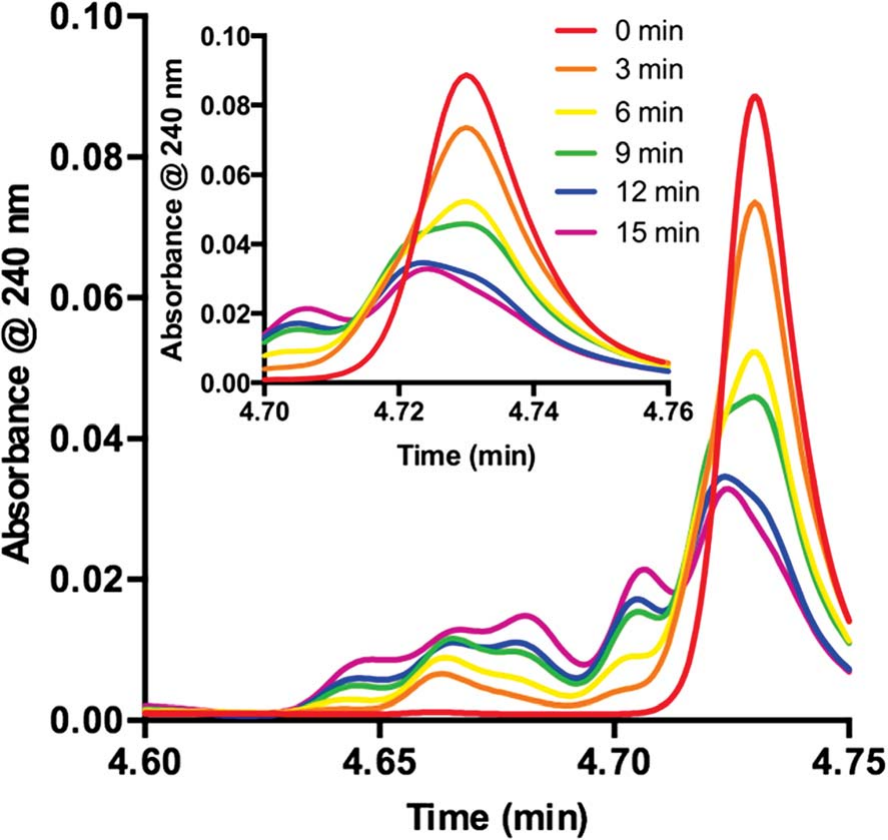
Consumption of **1** as observed during the first 15 minutes of the reaction displayed as an overlay of UPLC traces. Inset: UPLC trace from 4.70–4.76 min.

Knowing that **1** is functionalized over the course of the reaction, determination of the properties of this off-cycle pathway was necessary. Careful examination of the chromatographic data showed that the signal corresponding to **1** was greatly diminished in the first 15 min when the desired radical addition required 8 h to reach completion. To examine this process further, we probed the initial time period of the reaction in detail. Sampling every 3 min for the first 15 min of the reaction yielded a series of UPLC traces (Fig. 3). As the reaction proceeds from 0 (red) to 15 min (purple), the signal corresponding to **1** decreases with time. Conversely, a range of peaks with higher polarity (shorter retention time) formed as the reaction proceeded. Interestingly, these signals each appeared to form at different rates. Similar observations are present at the onset of the **5**-catalyzed system.^26^ Closer examination of the decaying signal of **1** (Fig. 3, inset) displays an interesting feature. At a slightly lower retention time than the peak maximum for **1**, a shoulder increases with time. This shoulder displays an identical retention time to **5**. These combined data indicate that **1** is almost entirely consumed in the first 15 min of the reaction. However, 8 h are required for complete consumption of **2**, suggesting that **1** is not the active catalyst for the majority of the transformation.

**Fig. 4:**
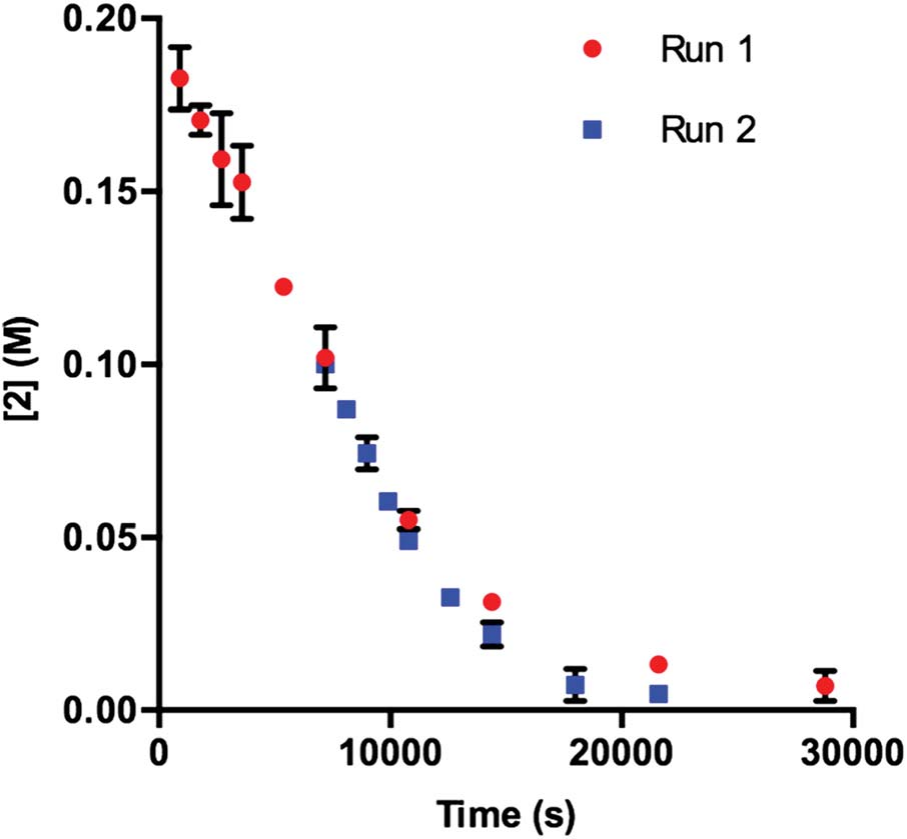
Profile of Run 1 as [**2**] *vs.* time and the time-adjusted profile of Run 2 as [**2**] *vs.* adjusted time for the **1**-catalyzed reaction at 2 mol% catalyst.^24^

In DMA, the solubility of **1** is limited. When amounts of **1** greater than 7.50 μmol are used in the reaction, insoluble complex is visibly apparent. The limited solubility masks the rate order of the catalyst when **1** is added in amounts larger than 3.75–7.50 μmol.^26^ When deactivation is taken into consideration alongside the limited solubility of **1**, a potential issue presents itself: deactivation of the catalyst could be masked due to phase transfer. To examine this supposition, we probed catalyst stability on the model system utilizing 2 mol% **1** (40 μmol) with respect to **2**. These data, shown in Fig. 4, do in fact display deactivation of the catalyst; however, the degree of deactivation appears to be suppressed compared to when 0.375 mol% **1** is utilized (Fig. 1). Given the solubility of **1** in DMA, only a percentage of the catalyst is present in solution at any given time. As functionalization of the catalyst occurs, more **1** dissolves. This process acts as an *in situ* slow addition to maintain a sufficient level of active catalyst at any given point during the course of the reaction. It is important to note that in a system that requires efficient irradiation, the presence of insoluble catalyst will scatter the light and as a consequence decrease the efficiency of the reaction.

**Fig. 5:**
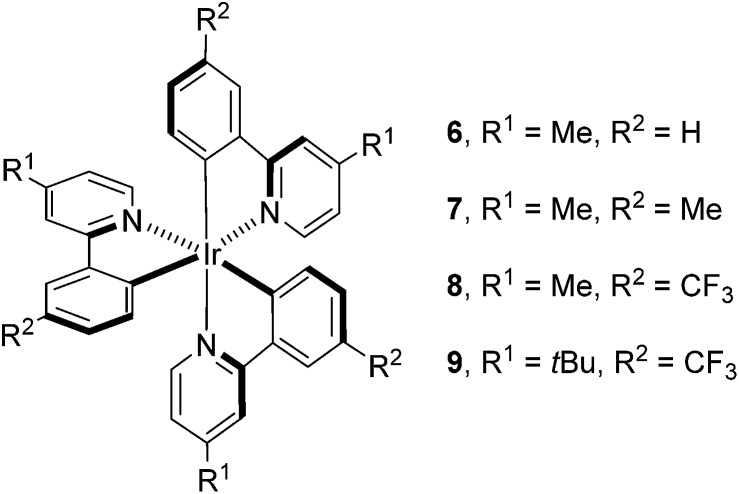
Mechanistically directed catalyst design.

**Fig. 6:**
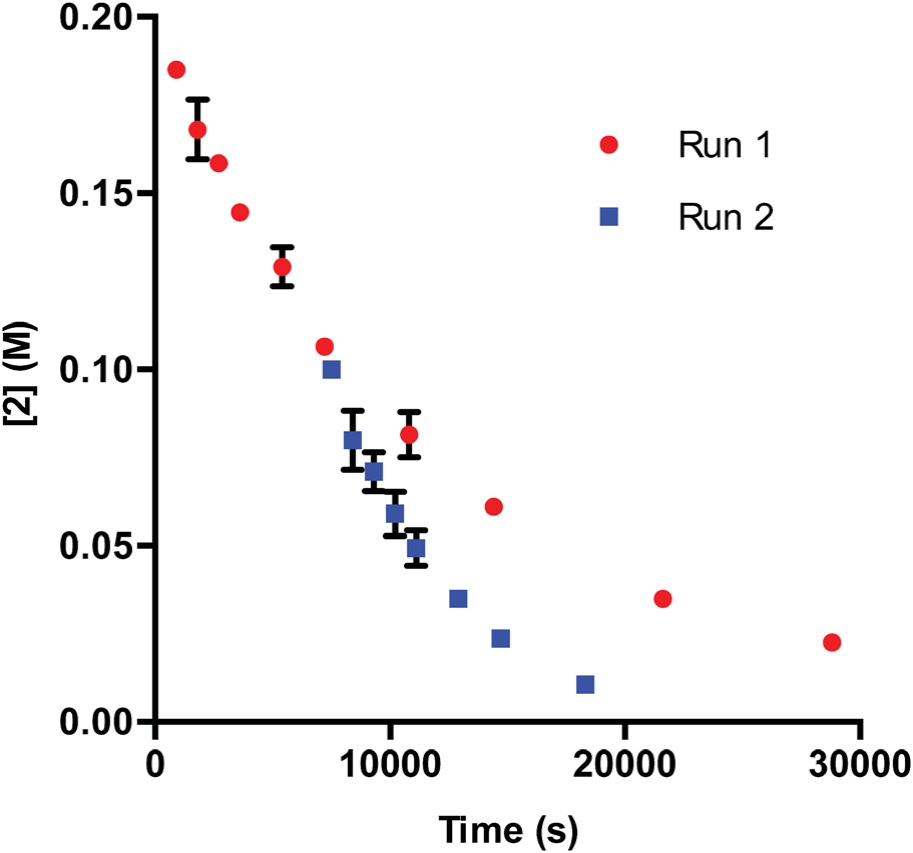
Profile of Run 1 as [**2**] *vs.* time and the time-adjusted profile of Run 2 as [**2**] *vs.* adjusted time.^24^

Having determined the probable cause of deactivation of **1**, we next examined if inhibition of this process was possible by functionally blocking potential sites of alkylation. The design principle utilized to propose new catalyst structures focused on the positions *para* to the C–Ir and N–Ir bonds of the ligands (Fig. 5).^25^ Knowing that the *in situ* monoalkylation of the phenyl ring of the catalyst did not stop the reaction, we initially attempted to block sterically the pyridine rings (**6**). This substitution pattern provided a functional catalyst, capable of facilitating the reaction. Kinetic analysis of the stability of complex **6** is consistent with deactivation of the catalyst to a decreased extent (Fig. 6).^26^ Furthermore, this catalyst was competent at lower catalyst loading when compared to **1** – 94% conversion was obtained in 18 h using only 0.187 mol% of **6**. In contrast, the reaction initiated by catalyst **1** reached only 72% conversion for the same catalyst loading and reaction time. These results illustrate that catalyst design in concert with kinetic analysis can be used to develop novel photocatalysts with improved function.

Knowing that targeted design of the catalyst mitigated the observed deactivation of the complex, we next pursued structures bearing functional groups on all six *para*-positions relative to the Ir center. Complex **7**, possessing methyl groups at all six positions catalyzed the reaction; however, after 48 h of irradiation, the complete consumption of **2** did not occur (<50%). Electronically, complex **7** possesses six donor groups. This extra electron density should stabilize the Ir^iv^ oxidation state, making the complex a more powerful reductant and a weaker oxidant. Examination of the known electronic properties of this complex shows that the Ir^iv^ oxidation state of **7** (Ir^iv/iii^ = +0.49 V) is 220 mV weaker than that of **1** (Ir^iv/iii^ = +0.77 V).^27^ These data suggest that the donor groups on the phenyl rings, in addition to donor groups on the pyridyl rings, greatly impact catalyst performance. Based on this observation, complex **8** was prepared, substituting trifluoromethyl groups for the donor substituents on the phenyl rings. This complex also catalyzes the reaction, but does not proceed to completion. The reaction attained 71% conversion after 12 h and only increased to 75% conversion after 48 h, suggesting that the methyl groups present on the pyridine rings, while capable of blocking alkylation, may provide an avenue for benzylic functionalization of the complex.^28^ To eliminate this possibility, the methyl groups were replaced with *t*-butyl groups (**9**). The **9**-catalyzed system did not proceed to completion (<50% conversion after 48 h).

Finally, we utilized Stern–Volmer analysis in order to gain insight into the comparative quenching efficiencies of complexes **1**, **5**, **6**, **7**, **8**, and **9**, employing **3** as the quencher.^10^ The most efficiently quenched complex is **7**, indicated by a Stern–Volmer constant of 2.00 ± 0.05 M^–1^. **6** provided the second highest value of 0.65 ± 0.05 M^–1^, followed by native complex **1** (0.34 ± 0.02 M^–1^) and monoalkylated complex **5** (0.35 ± 0.01 M^–1^). Interestingly, quenching was not detected for either complex **8** (–0.03 ± 0.05 M^–1^) or **9** (0.02 ± 0.03 M^–1^). These results, combined with the fact that all complexes facilitated the process to some degree, suggest that quenching ability is not necessarily the absolute determining factor for catalytic reactivity.

## Conclusions

In summary, the studies presented herein reveal the complex behavior of a photocatalyst under reaction conditions through the following findings: (1) **1** deactivates over the course of the reaction. (2) The deactivation pathway is initiated *via* alkylation of **1**, presumably through radical addition. (3) Both **1** and **5** are consumed in the first 15 minutes of the 8 h reaction. (4) The [**1**] is limited under reaction conditions, leading to kinetic properties that are masked by phase transfer. (5) Modest structural modification of the catalyst can inhibit deactivation to a degree; whereas, significant changes can result in termination of reactivity. This work, along with the photodegradation studies of König *et al.*,^28^ indicate that synthetic chemists should consider *in situ* catalyst functionalization when designing new methods. This process is, most likely, not unique to photocatalysis. The ability of intermediate radicals to add to arene ligands may be a concern in all transition metal-mediated radical processes. This consideration is especially true of efforts to reuse catalyst through chromatographic isolation, attachment to a surface, or attachment to a polymer. We are currently examining the mechanisms of other photoredox systems to determine the generality of photocatalyst deactivation *via in situ* functionalization.
