# Supplementary material for: Ligand functionalization as a deactivation pathway in a fac-Ir(ppy)3-mediated radical addition
Source: Chem Sci. 2014 Oct 20;6(1):537–41. doi: 10.1039/c4sc03064h (PMC5491958; doi:10.1039/c4sc03064h)
Supplement: Supplementary file 1 [file SC-006-C4SC03064H-s001.pdf]

Electronic Supplementary Material (ESI) for Chemical Science.
This journal is © The Royal Society of Chemistry 2014

Supporting Information

Ligand functionalization as a deactivation pathway in a *fac*-Ir(ppy)$_3$ mediated radical addition

James J. Devery, III,[†] James J. Douglas,[†,‡] John D. Nguyen,[†] Kevin. P. Cole, [‡] Robert A. Flowers, II,[§] Corey R. J. Stephenson*,[†]

[†] Department of Chemistry, University of Michigan, Ann Arbor, MI 48105.

crjsteph@umich.edu

[‡]Process Design and Development, Lilly Research Laboratories, Eli Lilly and Company, Indianapolis, IN 46285

[§] Department of Chemistry, Lehigh University, Bethlehem, PA 18015

Table of Contents

1.0 General Information 3
2.0 Model Reaction 4
3.0 Procedure for Kinetic Analysis 4
4.0 Method for Graphical Analysis 5
5.0 Supplementary Rate Data 7
6.0 General Procedure for the Synthesis of the Photocatalysts 12
7.0 Synthesis of *fac*-Ir[((*p*-(CH_2C(O)OEt)PhPy)(ppy)$_2$] 5 19
8.0 Assignment for *fac*-Ir(ppy)$_3$ and comparison with 5 21
9.0 Transformation of 6 to to *fac*-Ir[((*p*-C(O)H)Ph)Py)(ppy)$_2$] S4 25
10.0 ^{13}C Coordination-Induced Shift Calculations 28
11.0 Photocatalyst Quenching Studies 30
12.0 Full Page Versions of Manuscript Figures 31
13.0 MS Data 36
14.0 NMR Spectra 48

1.0 General Information

Chemicals were either used as received or purified according to the procedures outlined in *Purification of Common Laboratory Chemicals.* Reactions were monitored by TLC and visualized by a dual short wave/long wave UV lamp and stained with an ethanolic solution of potassium permanganate, ceric ammonium molybdate, or anisaldehyde. Column flash chromatography was performed using 230-400 mesh silica gel or via automated column chromatography. Yields refer to chromatographically and spectroscopically pure compounds, unless otherwise noted. LED lights were purchased from Creative Lighting Solutions (http://www.creativelightings.com).

^{1}H, ^{13}C and ^{19}F NMR spectra were recorded using an internal deuterium lock on Varian Unity Plus 400, Varian 500, or a Varian 700 spectrometers. All signals are reported in ppm with the internal reference of the specified solvent. J couplings are reported in Hz. Data are presented as follows: integration, multiplicity (s = singlet, d = doublet, t = triplet, q = quartet, m = multiplet, br = broad, app = apparent, dd = doublet of doublet, dt = doublet of triplet, etc) and coupling constant (J/Hz).

Photocatalysts were assigned via comparison to those assigned by Watts[1] and by the coordination-induced shift calculation as described by Watts.[1] For *fac*-Ir((*p*-CF_3Ph)(*p*-MePy))$_3$ and related complex's *p*- is defined as the substituent position relative to the carbon-iridium or carbon-nitrogen bond. The numbering scheme for the ligands and the photocatalysts is based on that reported by Watts[1] for 2-phenylpyridine and has remained consistent throughout for ease of comparison.

Infrared spectra were recorded on a Perkin Elmer BX FT-IR fitted with an ATR accessory. Absorptions are given in wavenumbers (cm^{-1}). High resolution mass spectra were obtained on a Waters® Micromass® AutoSpec Ultima™ high resolution mass spectrometer. Photocatalyst quenching was conducted on a Fluoromax-2 fluorimeter and the values represent an average of 3 samples. UPLC analysis was conducted on a Waters® Acuity H-Class UPLC with a Acquity UPLC BEH C18 column 1.7 μm 2.1 x 50mm. GC-MS was performed on a Shimadzu QP2010 SE fitted with a AOC-20i auto injector.

[1] Grace, F. O.; Watts, R. J. *Magn. Reson. Chem.* **1993**, *31*, 529.

2.0 Model Reaction

Ethyl 2-(3-methyl-1H-indol-2-yl)acetate 4

A 10 mL round bottom flask was equipped with a rubber septum and magnetic stir bar and was charged with *fac*-Ir(ppy)$_3$ (5 mg, 1.0 μmol, 0.00375 equiv), $NaHCO_3$ (0.16 g, 2.0 mmol, 1.0 equiv), 3-methyl indole (0.26 g, 2.0 mmol, 1.0 equiv), ethyl bromoacetate (0.66 mL, 6.0 mmol, 3.0 equiv), DMA (10.0 mL). The heterogeneous mixture was sparged with N_2 gas while stirring for 15 minutes. The flask was then irradiated with a 1 W blue LED strip. After the reaction was complete (12 h, as judged by TLC analysis), the solution was washed with 5% aqueous LiCl (50 mL) and extracted with diethyl ether (2 x 25 mL). The combined organic layers were washed with brine, dried (Na_2SO_4), and concentrated. The residue was purified via Combiflash using silica gel and a hexanes-EtOAc gradient to provide **4** (0.37 g, 85%) as colorless oil with spectroscopic properties in accordance with those reported in the literature.[2]

^{1}H NMR ($CDCl_3$, 400 MHz): δ_H 1.29 (3H, t, *J* 7.6), 3.76 (2H, s), 2.56 (3H, s), 4.20 (2H, q, *J* 7.2), 7.09 (1H, t, *J* 8.0), 7.16 (1H, t, *J* 8.0), 7.31 (1H, d, *J* 8.0), 7.50 (1H, d, *J* 8.0) and 8.49 (1H, br s).

3.0 Procedure for Kinetic Analysis

A 25 mL recovery flask equipped with a magnetic stir bar was charged with **1**, **2**, and $NaHCO_3$. 10 mL DMA was added to the flask and it was sealed with a rubber septum. **3** was added via syringe. The slurry was sparged with N_2 for 15 min. The reaction was then illuminated with a 1W blue LED strip and stirred under N_2 atmosphere. 0.05 mL aliquots were sampled via syringe. 10 μL reaction solution was dissolved 990 μL 2:1 MeCN:H_2O containing 10 μL of 5 mg/mL acetophenone in 2:1 MeCN:H_2O. Samples were analyzed via reverse-phase UPLC.

[2] Kapur, A.; Kumar, K.; Singh, L.; Singh, P.; Elango, M.; Subramanian, V.; Gupta, V.; Kanwai, P.; Ishar, M. P. S. *Tetrahedron* **2009**, *65*, 4593.

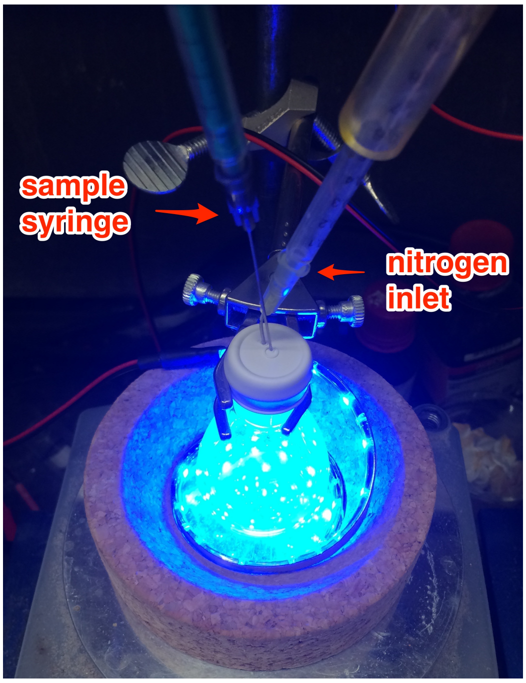

Figure S1. Set up for kinetic analysis

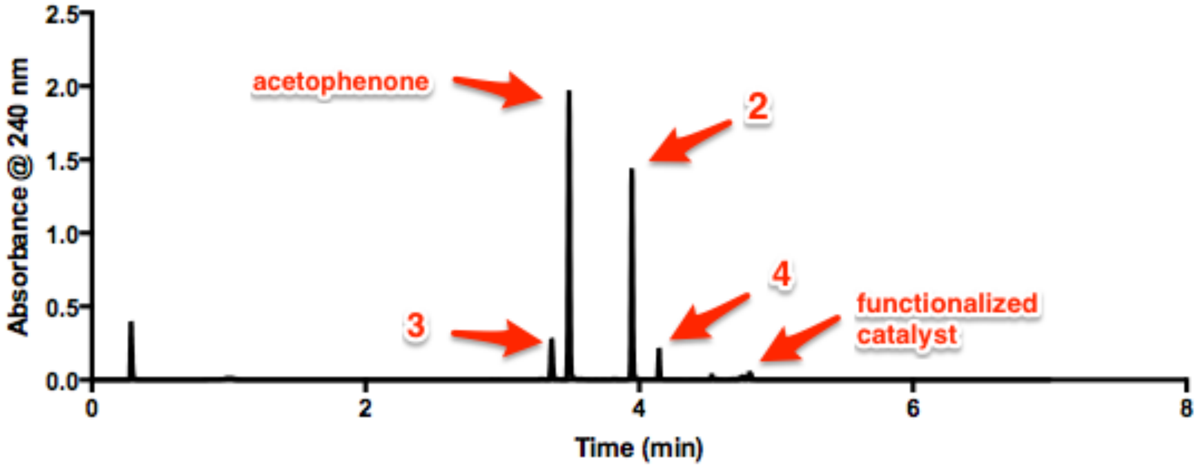

Figure S2. Representative UPLC trace.

4.0 Method for Graphical Analysis

The concentration of the limiting substrate is measured as a function of time and plotted. The rate of reaction is calculated from the slope of this curve. The raw data is approximated using mathematical curve-fitting software to determine an equation that represents the full data set over the time observed. The resulting function, $f(time)$, is used to determine the concentration of substrate for all times measured. The derivative of this function ($f'(time)$) is a mathematical approximation of the rate of reaction for all times measured. Then, $f'(time)$ and $f(time)$ are treated as parametric equations for all times measured over the course of the experiment, where $y = f'(time)$ and $x = f(time)$.

The data are then fit to a first order exponential decay in Prism 6 software using the following equation:

$$f(time) = [\mathbf{2}] = y_0 + Ae^{-k_{obs}(time)}$$

where constants A, k_{obs}, and y_0 are calculated by the software. A R^2 value ≥0.99 was required to determine an accurate approximation of the data. The standard deviation with respect to [**2**] was determined using the following equation and standard deviations determined by the software:

$$\sigma_{[\mathbf{3}]} = Ae^{-k_{obs}(time)}\sqrt{\left(-k_{obs}e^{-k_{obs}(time)}(time)(\sigma_{k_{obs}})\right)^2 + \left(\frac{\sigma_A}{A}\right)^2 + \sigma_{y_0}{}^2}$$

The derivative [**2**] provided the equation for rate of reaction as follows:

$$f'(time) = -\frac{d[\mathbf{2}]}{dt} = \quad k_{obs}Ae^{-k_{obs}(time)}$$

where the uncertainty with respect to rate is defined as

$$\sigma_{\frac{d[\mathbf{2}]}{dt}} = k_{obs}Ae^{-k_{obs}(time)}\sqrt{\left(-k_{obs}e^{-k_{obs}(time)}(time)(\sigma_{k_{obs}})\right)^2 + \left(\frac{\sigma_A}{A}\right)^2 + \left(\frac{\sigma_{k_{obs}}}{k_{obs}}\right)^2}$$

5.0 Supplementary Rate Data

Functionalization of 1

An initial UPLC trace of 1 was obtained (Figure S3, blue). In the absence of **2**, **1** and **3** were irradiated under reaction conditions for 2 h (Figure S3, black). We sampled this mixture and obtained a UPLC trace that displayed no obvious peak corresponding to **1** and a variety of other peaks. We then added the typical **2** (2 mmol) to the reaction mixture and irradiated this system with light. After 2 h, the mixture was sampled and a UPLC trace obtained which displayed 0.030 mmol conversion of **2** (Figure S3, red).

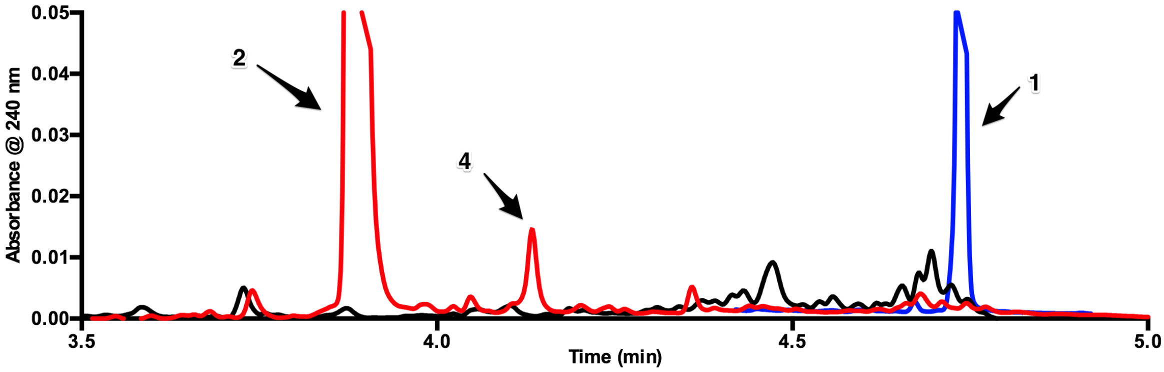

Figure S3. UPLC trace of **1** before irradiation (blue). UPLC trace of **1** after 2 h irradiation (black). UPLC trace of reaction mixture after 2 h of irradiation (red).

Deactivation of 5

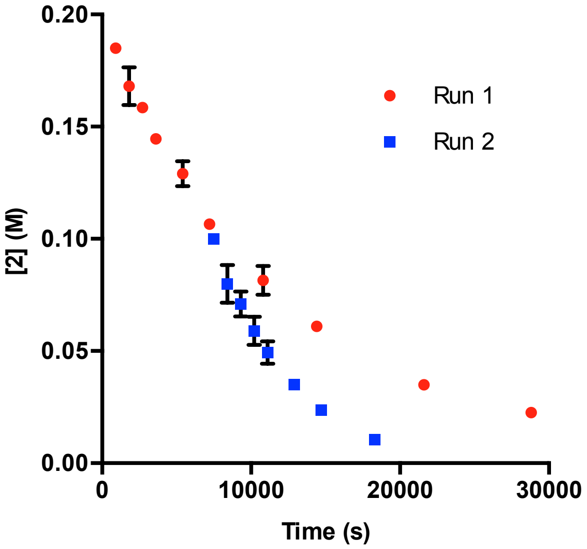

Figure S4. Profile of Run 1 as [**2**] vs time and the time-adjusted profile of Run 2 as [**2**] vs adjusted time for the **5**-catalyzed system. Run 1: **5** = 7.5 μmol. **2** = 2.00 mmol, **3** = 6.00 mmol, $NaHCO_3$ = 4.00 mmol, DMA = 10 mL. Run 2: **5** = 7.5 μmol. **2** = 1.00 mmol, **3** = 5.00 mmol, $NaHCO_3$ = 3.00 mmol, DMA = 10 mL. All points are averaged from three reactions.

Initial consumption of 5

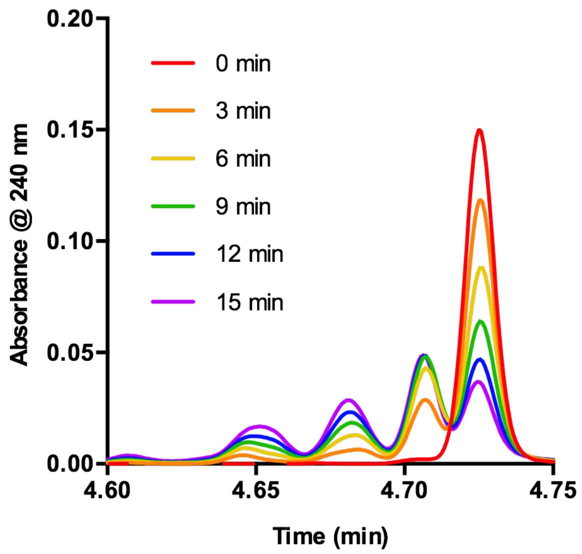

Figure S5. Consumption of **5** during the first 15 min of the reaction displayed as an overlay of UPLC traces. Conditions: **5** = 7.5 μmol. **2** = 2.00 mmol, **3** = 6.00 mmol, $NaHCO_3$ = 4.00 mmol, DMA = 10 mL.

Rate Order of 1

We considered the impact of [**1**] on the rate of reaction. Surprisingly, when the amount of **1** added to the reaction mixture was doubled from 7.50 μmol to 15.00 μmol, no change in rate occurred. Decrease in the amount of **1** to 3.75 μmol resulted in a slight decrease in the rate of reaction. While not visually apparent, these data suggest that when 7.50 μmol of **1** are added to the reaction mixture, not all of the catalyst is in solution. As a result, when additional **1** is added to the system, the [**1**] remains constant, providing no rate enhancement. A decrease in the amount of **1** to 3.75 μmol provided a minimal decrease in reaction rate. Because we know that the system must be near saturation based on the result at higher amounts of **1**, a further decrease in **1** is necessary to accurately observe homogenous concentration effects with respect to **1**. A reaction performed with 1.88 μmol yielded a more significant decrease in the rate of reaction. It is important to note that below this catalyst loading, the reaction does not proceed through three half-lives. The combined kinetic data suggest that the reaction is positive order in catalyst.

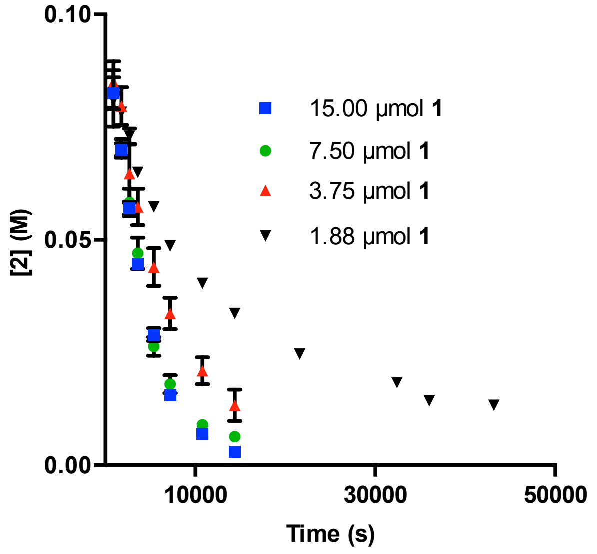

Figure S6. Effect of **1** on rate plotted as [**2**] vs time. General conditions: **2** = 1.00 mmol, **3** = 5.00 mmol, $NaHCO_3$ = 3.00 mmol, DMA = 10 mL. All points are averaged from three reactions.

Three different methods were utilized to determine the rate order of **1**. All three methods utilized **1** in 10 mL DMA.

Method 1: initial rates

Using the initial 6 points of decays determined measured for 3.75 μmol **1** and 1.88 μmol **1**, the slopes of the lines were determined using Prism 6 software and are given as follows:

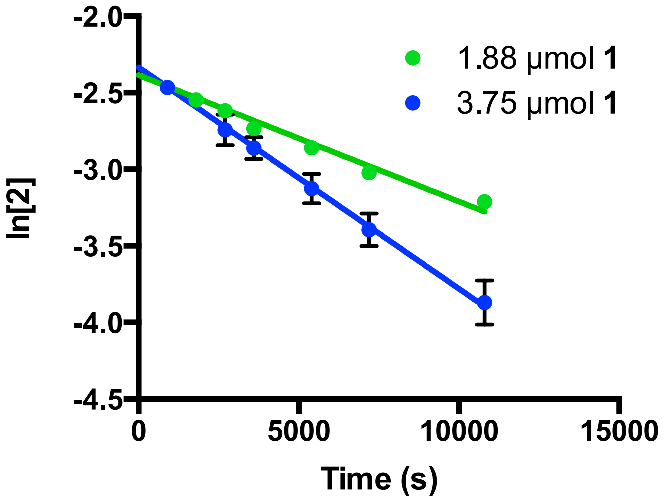

3.75 μmol **1**: R^2 fit = 0.9420; slope = $-1.05 \times 10^{-5} \pm 7 \times 10^{-7}$

1.88 μmol **1**: R^2 fit = 0.9066; slope = $-7.2 \times 10^{-6} \pm 6 \times 10^{-7}$

These data were used to determine the rate order as follows:

$$order = \frac{log\left(\frac{slope_1}{slope_2}\right)}{log\left(\frac{concentration_1}{concentration_2}\right)} = \frac{log\left(\frac{-1.05x10^{-5}}{-7.2x10^{-6}}\right)}{log\left(\frac{0.00075}{0.000375}\right)} = 0.54$$

Method 2: Graphical analysis of normalized rate

Using the typical method, the data were approximated using Prism 6. Then considered according to the rate equation

$$-\frac{d[\mathbf{2}]}{dt} = -k_{obs}[\mathbf{2}][\mathbf{1}]^x$$

The rate data can be directly compared through normalization of the reaction rate by the [**1**]^x

$$-\frac{\frac{d[\mathbf{2}]}{dt}}{[\mathbf{1}]^x} = -k_{obs}[\mathbf{2}]$$

where x is the rate order of **1**. Overlay of the two decays occurs when x = 0.85.

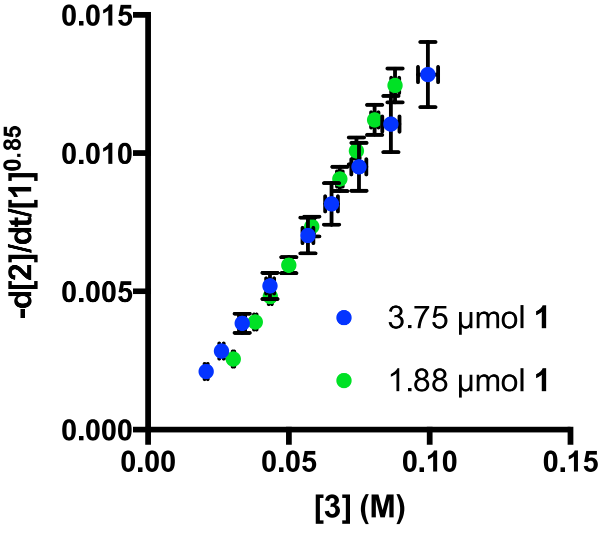

Method 3: ln(k_{obs}) vs ln([1])

Values for k_{obs} can be determined through determination of the slope of ln[**2**] vs time because

$$[\mathbf{2}] = [\mathbf{2}]_0 e^{-k_{obs}(time)}$$

therefore,

$$\ln([\mathbf{2}]) = \ln([\mathbf{2}]_0) - k_{obs}(time)$$

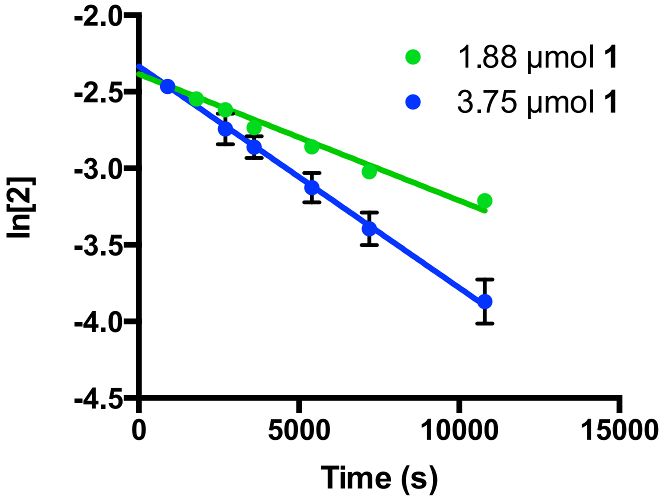

3.75 µmol **1**: R^2 = 0.9791; slope = -1.45 x 10^{-4} ± 5 x 10^{-6}
1.88 µmol **1**: R^2 = 0.9688; slope = -8.3 x 10^{-5} ± 3 x 10^{-6}

A rate order of 1.3 was determined through the following equation:

$$order = \frac{\ln(k_{obs1}) - \ln(k_{obs2})}{\ln(concentration_1) - \ln(concentration_2)} = \frac{ln(1.45x10^{-4}) - \ln(8.3x10^{-5})}{ln(0.00075) - ln(0.000375)} = 1.3$$

On/Off Experiment

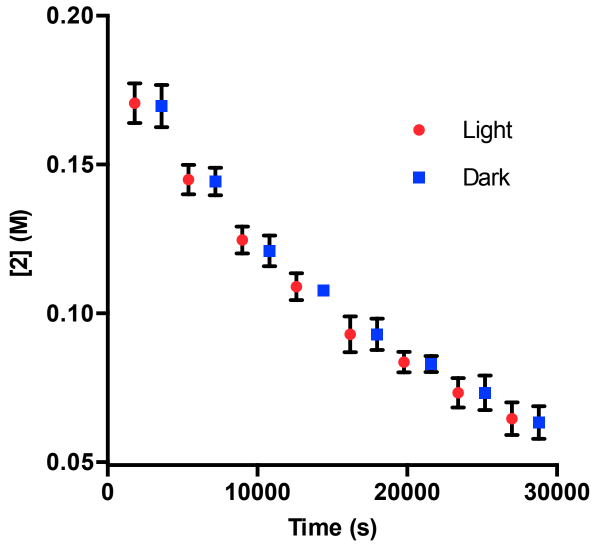

Figure S7. Analysis of the role of light plotted as [**3**] vs time. Concentrations designated as Light (red) were sampled after 30 min of irradiation. Concentrations designated as Dark (blue) were sampled after 30 min of darkness. Conditions: **1** = 7.5 µmol. **2** = 2.00 mmol, **3** = 6.00 mmol, $NaHCO_3$ = 4.00 mmol, DMA = 10 mL. Data consistent with propagation not being the dominant mechanism.

Degree of Deactivation of 6

Figure S8 displays a direct comparison of the reaction profiles for the **1**- and **6**-catalyzed systems. Figure S8a compares the Run 1 conditions for both systems while Figure S8b compares Run 2 conditions. Examination of Run 1 shows that **6** provides a

higher overall rate than **1**. However, under Run 2 conditions, **1** and **6** provide statistically similar rates. The difference in Figure S8a is consistent with a decrease in the degree of deactivation of the complex.

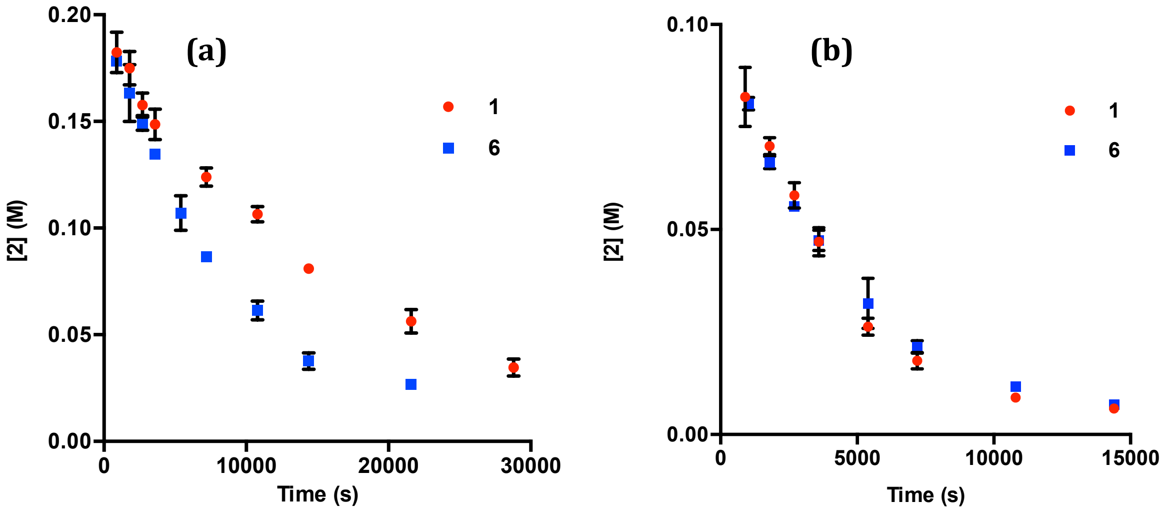

Figure S8. (a) Profile of Run 1 as [**2**] vs time for the **1-** and **6**-catalyzed systems Run 1: catalyst = 7.5 μmol. **2** = 2.00 mmol, **3** = 6.00 mmol, $NaHCO_3$ = 4.00 mmol, DMA = 10 mL. (b): Profile of Run 2 as [**2**] vs time for the **1-** and **6**-catalyzed systems Run 2: catalyst = 7.5 μmol. **2** = 1.00 mmol, **3** = 5.00 mmol, $NaHCO_3$ = 3.00 mmol, DMA = 10 mL. All points are averaged from three reactions.

6.0 General Procedure for the Synthesis of the Photocatalysts

$IrCl_3$ hydrate (1 equiv), ligand (9 equiv), KI (9 equiv) and ethylene glycol (39 mM) were combined in a microwave vial equipped with a magnetic stir bar and microwaved at 200 ^oC for 50 min. The crude mixture was diluted with H_2O. The slurry was vacuum filtered through a medium fritted funnel to isolate a solid. The solid was washed with H_2O and then dissolved with CH_2Cl_2 and filtered. The frit was washed with CH_2Cl_2 until the filtrate was colorless. The filtrate was dried with sodium sulfate and concentrated via rotary evaporation to give a solid.

The solid was combined with ligand (5 equiv relative to initial $IrCl_3$ hydrate) in H_2O (0.12 M) in an acid digestion bomb equipped with a magnetic stir bar. The bomb was then heated in a sand bath to 250 ^oC for 12 h. Upon cooling to ambient temperature, the crude mixture was diluted with H_2O. The slurry was vacuum filtered through a medium fritted funnel to isolate a solid. The solid was washed with H_2O and then dissolved with CH_2Cl_2 and filtered. The frit was washed with CH_2Cl_2 until the filtrate was colorless. The filtrate was dried with sodium sulfate and concentrated via rotary evaporation to give a solid. This crude solid was purified via Combiflash using silica gel and a hexanes-CH_2Cl_2 gradient.

***fac*-Ir(Ph(*p*-MePy))$_3$ 6**

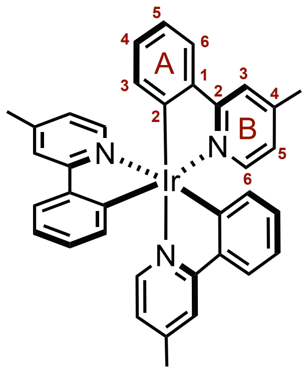

Prepared via the general procedure (110 mg, 28% yield) with spectroscopic properties in accordance with those reported in the literature.[3]

^{1}H NMR (700 MHz, CD_2Cl_2) δ_H 2.41 (9H, s, Py-C*H*$_3$), 6.71 (3H, d, *J* 7.3, *H*-A3), 6.74-6.78 (6H, m, *H*-B5 and *H*-A4), 6.85 (3H, t, *J* 7.1, *H*-A5), 7.42 (3H, d, *J* 5.6, *H*-B6), 7.64 (3H, d, *J* 7.9, *H*-A6) and 7.72 (3H, s, *H*-B3);

^{13}C NMR (176 MHz, CD_2Cl_2) δ_c 21.4 (Py-*C*H$_3$), 119.8 (*C*-B3), 119.9 (*C*-A5), 123.5 (*C*-B5), 124.1 (*C*-A6), 129.7 (*C*-A4), 137.1 (*C*-A3), 144.3 (*C*-A2), 146.9 (*C*-B6), 148.1 (*C*-B4), 161.8 (*C*-A1) and 166.2 (*C*-B2).

[3] Yang, C.-H., Fang, K.-H.; Chen, C.-H.; Sun, I.-W. *Chem. Comm.* **2004**, 2232.

fac-Ir((*p*-MePh)(*p*-MePy))$_3$ 7

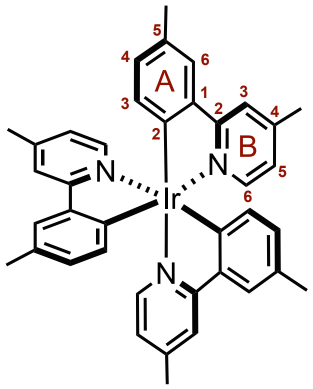

Prepared via the general procedure (70 mg, 17% yield) with spectroscopic properties in accordance with those reported in the literature.[4]

^{1}H NMR (700 MHz, CD_2Cl_2) δ_H 2.26 (9H, s, Ph-C*H*$_3$), 2.40 (9H, s, Py-C*H*$_3$), 6.57 (3H, d, *J* 7.6, *H*-A3), 6.62 (3H, dd, *J* 7.6, 1.4, *H*-A4), 6.70 (3H, dd, *J* 5.6, 1.1, *H*-B5), 7.37 (3H, d, *J* 5.6, *H*-B6), 7.47 (3H, s, *H*-A6) and 7.70 (3H, s, *H*-B3);

^{13}C NMR (176 MHz, CD_2Cl_2) δ_c 21.2 (Ph-*C*H$_3$), 21.4 (Py-*C*H$_3$), 119.7 (*C*-B3), 123.4 (*C*-B5), 124.7 (*C*-A6), 128.8 (*C*-A5), 130.9 (*C*-A4), 137.0 (*C*-A3), 144.4 (*C*-A2), 146.9 (*C*-B6), 147.8 (*C*-B4), 157.4 (*C*-A1) and 166.3 (*C*-B2).

4-methyl-2-(3-(trifluoromethyl)phenyl)pyridine S1

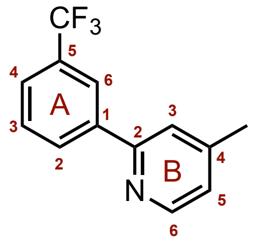

To two identical 20 mL microwave vials was added (3-(trifluoromethyl)phenyl)boronic acid (360 mg, 1.88 mmol, 1.2 equiv), K_2CO_3 (430 mg, 3.14 mmol, 2.0 equiv), Bis(triphenylphosphine)palladium(II) dichloride (11.0 mg, 0.02 mmol, 0.01 equiv), 1,4-dioxane (10.5 mL), water (4.50 mL) and 2-bromo-4-methylpyridine (170 µL, 1.57 mmol, 1.0 equiv) and the heterogeneous solution sparged with nitrogen for 5 mins. The vials were sealed and heated in a microwave at 150 °C for 50 mins before cooling to rt,

[4] Jung, S.; Kang, Y.; Kim, H.-S.; Kim, Y.-H.; Lee, C.-L.; Kim, J.-J.; Lee, S.-K.; Kwon, S.-K. *Eur. J. Inorg. Chem.* **2004**, 3415.

combining and diluting with Et_2O (50 mL). The black heterogeneous solution was filtered through celite to give a light orange solution that was washed twice with water (50 mL), dried (Na_2SO_4), filtered and concentrated *in vacuo* to give an orange oil. This crude material was purified via automated column chromatography (12 g silica column, eluting with 5% EtOAc:hexanes) to give 4-methyl-2-(3-(trifluoromethyl)phenyl)pyridine **S1** (370 mg, 76%) as a colorless crystalline solid.

^{1}H NMR (700 MHz, $CDCl_3$) δ_H 2.45 (3H, s, Py-C*H*$_3$), 7.12 (1H, dd, *J* 5.0, 0.7, *H*-B5), 7.57-7.60 (2H, m, *H*-B3, H-A3), 7.66-7.67 (1H, m, *H*-A4), 8.18 (1H, d, *J* 7.8, *H*-A2), 8.26 (1H, s, *H*-A6) and 8.58 (1H, dd, *J* 5.0, 0.4, *H*-B6);

^{13}C NMR (176 MHz, $CDCl_3$) δ_c 21.3 (Py-*C*H$_3$), 121.6 (*C*-B3), 123.9 (q, *J* 3.9, *C*-A6), 123.9 (*C*-B5), 124.3 (q, *J* 272, *C*F$_3$), 125.5 (q, *J* 3.7, *C*-A4), 129.2 (*C*-A3), 130.2 (*C*-A2), 131.2 (q, *J* 32.3, *C*-A5), 140.4 (*C*-A1), 148.2 (*C*-B4), 149.7 (*C*-B6) and 155.8 (*C*-B2);

^{19}F NMR (376 MHz, $CDCl_3$) δ_F −62.3;

ν_{max} (ATR)/cm^{-1} 2921, 2182, 1606, 1429, 1335, 1285, 1153, 1102, 1091, 1077, 910, 804, 700 and 670;

m/z: (ESI+) HRMS [M+H] $C_{13}H_{10}F_3N^+$: found 238.0848; calcd 238.0838.

fac-Ir((*p*-CF$_3$Ph)(*p*-MePy))$_3$ 8

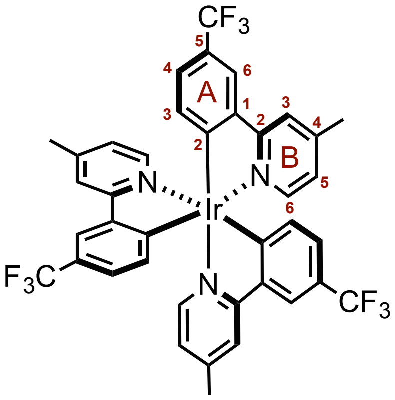

Prepared via the general procedure (270 mg, 53% yield).

^{1}H NMR (700 MHz, CDCl$_3$) δ_H 2.47 (9H, s, Py-C*H*$_3$), 6.81 (3H, d, *J* 5.4, *H*-B5), 6.85 (3H, d, *J* 7.8, *H*-A3), 7.01 (3H, d, *J* 8.1, *H*-A4), 7.37 (3H, d, *J* 5.6, *H*-B6), 7.76 (3H, s, *H*-A6) and 7.82 (3H, s, *H*-B3);

^{13}C NMR (176 MHz, CDCl$_3$) δ_c 21.6 (Py-*C*H$_3$), 120.19 (*C*-B3) overlapping 120.20 (app s, *C*-A6), 122.4 (q, *J* 32, *C*-A5), 124.2 (*C*-B5), 125.7 (q, *J* 271, Ph-*C*F$_3$), 126.0 (q, *J* 3.3, *C*-A4), 137.1 (*C*-A3), 144.2 (*C*-A2), 146.7 (*C*-B6), 148.5 (*C*-B4), 164.9 (*C*-B2) and 166.2 (*C*-A1);

^{19}F NMR (376 MHz, CDCl$_3$) δ_F −62.6;

ν_{max} (ATR)/cm^{-1} 2158, 1615, 1599, 1328, 1269, 1103, 1075, 1029, 820 and 683;

m/z: (ESI+) HRMS [M+H] $C_{39}H_{27}F_9IrN_3^+$: found 902.1741; calcd 902.1763.

4-(tert-butyl)-2-(3-(trifluoromethyl)phenyl)pyridine S2

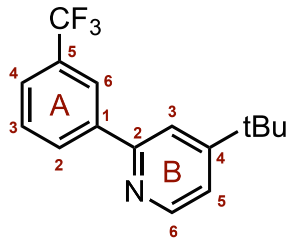

To a 20 mL microwave vial was added (3-(trifluoromethyl)phenyl)boronic acid (4.20 g, 22.1 mmol, 1.5 equiv), K_2CO_3 (6.11 g, 44.2 mmol, 3.0 equiv), Tetrakis(triphenylphosphine)palladium(0) (510 mg, 0.442 mmol, 0.03 equiv), THF (9.0 mL), water (3.0 mL) and 2-chloro-4-tertbutylpyridine[5] (2.5 g, 14.7 mmol, 1.0 equiv) and the heterogeneous solution sparged with nitrogen for 5 mins. The vials were sealed and heated in a microwave at 150 °C for 50 mins before cooling to rt, combining and diluting with Et_2O (50 mL). The black heterogeneous solution was filtered through celite to give a light orange solution that was washed twice with water (50 mL) once with Na_2CO_3, dried (Na_2SO_4), filtered and concentrated *in vacuo*. This crude material was purified via automated column chromatography (40 g silica column, eluting with 0% to 100% EtOAc:hexanes) to give 4-(tert-butyl)-2-(3-(trifluoromethyl)phenyl)pyridine **S2** (4.12 g, 62%) as a pale yellow oil.

^{1}H NMR (700 MHz, $CDCl_3$) δ_H 1.37 (9H, s, Py-C(C*H*$_3$)$_3$), 7.28 (1H, dd, *J* 5.2, 1.8, *H*-B5), 7.58 (1H, t, *J* 7.7, *H*-A3), 7.65 (1H, d, *J* 7.8, *H*-A4), 7.71 (1H, app t, *J* 0.9, *H*-B3), 8.15(1H, d, *J* 7.8, *H*-A2), 8.23 (1H, s, *H*-A6) and 8.61-8.62 (1H, m, *H*-B6);

^{13}C NMR (176 MHz, $CDCl_3$) δ_c 30.5 (Py-C(*C*H$_3$)$_3$), 34.9 (Py-*C*(CH$_3$)$_3$), 117.8 (*C*-B3), 120.1 (*C*-B5), 123.9 (d, *J* 3.7, *C*-A6), 124.2 (q, *J* 272, Ph-*C*F$_3$), 125.3 (d, *J* 3.6, *C*-A4), 129.1 (*C*-A3), 130.2 (*C*-A2), 131.1 (q, *J* 32, *C*-A5), 140.7 (*C*-A1), 149.7 (*C*-B6), 155.9 (*C*-B2), and 161.2 (*C*-B4);

^{19}F NMR (376 MHz, $CDCl_3$) δ_F −62.6;

ν_{max} (ATR)/cm^{-1} 2965, 2243, 1600, 132, 1162, 1120, 1063, 801, 697 and 663;
m/z: (ESI+) HRMS [M+H] $C_{16}H_{16}F_3N^+$: found 280.1318; calcd 280.1308.

[5] Fu, R.; Bercaw, J. E.; Labinger, J. A. *Organometallics* **2011**, *30*, 6751.

***fac*-Ir((*p*-CF$_3$Ph)(*p*-^{t}BuPy))$_3$ 9**

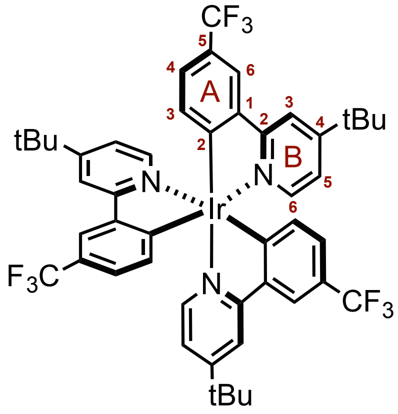

Prepared via the general procedure (20 mg, 11% yield).

^{1}H NMR (700 MHz, CDCl$_3$) δ_H 1.35 (9H, s, Py-C(C*H*$_3$)$_3$, 6.87 (3H, Br S, *H*-A3), 7.00 (3H, dd, *J* 5.9, 1.6, *H*-B5), 7.02 (3H, d, *J* 7.6, *H*-A4), 7.37 (3H, d, *J* 5.9, *H*-B6), 7.83 (3H, s, *H*-A6) and 7.89 (3H, d, *J* 1.6, *H*-B3);

^{13}C NMR (176 MHz, CDCl$_3$) δ_c 30.5 (Py-C(*C*H$_3$)$_3$), 35.1 (Py-*C*(CH$_3$)$_3$), 116.0 (*C*-B3), 119.9 (app d, *J* 3.0, *C*-A6), 120.5 (*C*-B5), 122.2 (q, *J* 33.0, *C*-A5), 125.5 (q, *J* 271, Ph-*C*F$_3$), 125.7 (app d, *J* 2.5, *C*-A4), 137.1 (*C*-A3), 144.4 (*C*-A2), 146.6 (*C*-B6), 161.0 (*C*-B4), 164.6 (*C*-B2) and 166.1 (*C*-A1);

^{19}F NMR (376 MHz, CDCl$_3$) δ_F −61.5;

ν_{max} (ATR)/cm^{-1} 2360, 1327, 1276, 1117, 1072, 1060, 1031, 835 and 606;

m/z: (ESI+) HRMS [M+H] C$_{48}$H$_{45}$F$_9$IrN$_3^+$: found 1027.3088; calcd 1027.3094.

7.0 Synthesis of *fac*-Ir[((*p*-(CH$_2$C(O)OEt)PhPy)(ppy)$_2$] 5

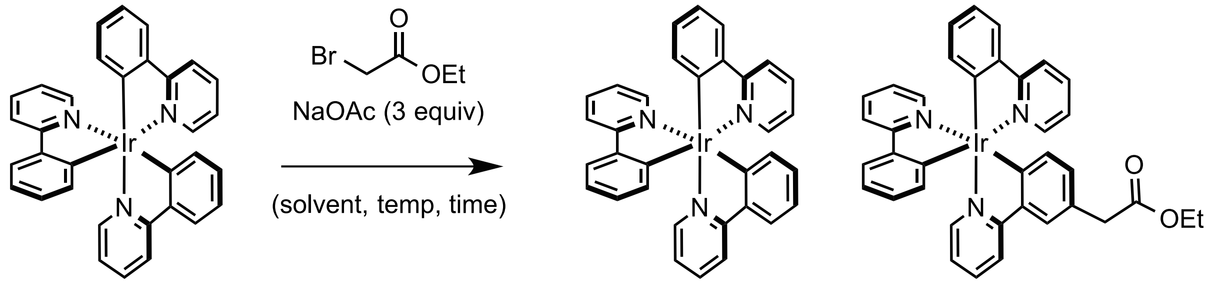

1 **1** **5**

Table S1: Synthesis of 5; optimization

Equiv of ethyl-bromoacetate[a]	Scale/mg	Solvent	Temp/°C	Time/h	Yield of Ir(ppy)$_3$	Yield of 5
10	20	DMA	22	4	-	18
2	25	DMA	10	5	52	4
3	25	DCM	10	5	48	14
3	50	DCM	5	13	30	27
3	200	DCM	5	13.5	13	24
3	***200***	***DCM***	***5***	***9.5***	***35***	***29***
3[b]	20	DCM	5	9.5	>95	

[a] The use of higher equivalents of ethylboroacetate representative of the reaction conditions led to an intractable mixture of products within 1h. [b] Reaction performed in the dark.

A heterogeneous solution of *fac*-Ir(ppy)$_3$ (200 mg, 0.305 mmol, 1.0 equiv), sodium acetate (75.1 mg, 0.916 mmol, 3.0 equiv) and ethylbromoacetate (101 μL, 0.916 mmol, 3.0 equiv) in CH$_2$Cl$_2$ (40 mL) in a 100 mL RBF was cooled to 5 °C in a 100 mL jacketed beaker connected to a circulating chiller with *i*-PrOH as the internal beaker coolant (see picture for details). The heterogeneous solution was sparged with N$_2$ for 15 minutes resulting in minimal solvent loss and a color change from yellow to bright luminous green consistent with *fac*-Ir(ppy)$_3$. Two 1W LED strips were turned on and the heterogeneous solution stirred at 5 °C for 9.5 h. The solution was then diluted with CH$_2$Cl$_2$ (25 mL) and washed with water (25 mL), the organic portion was dried (Na$_2$SO$_4$), filtered and concentrated *in vacuo* to give a bright yellow crude material that was purified via automated column chromatography (40 g silica column, eluting with 30% hexanes:CH$_2$Cl$_2$ to CH$_2$Cl$_2$ to 1% MeOH:CH$_2$Cl$_2$) to give *fac*-Ir(ppy)$_3$ (70 mg, 35% yield), **5**

(66 mg, 29% yield) and a second mono alkylated *fac*-Ir(ppy)$_3$ complex **S3** (12 mg, 5% yield).

Data for *fac*-Ir[((*p*-(CH$_2$C(O)OEt)PhPy)(ppy)$_2$] 5

^{1}H NMR (700 MHz, DMSO) δ_H 1.16 (3H, t, *J* 7.1, CH$_2$C*H*$_3$), 3.49 (2H, s, ArC*H*$_2$C(O)) 4.05 (2H, q, *J* 7.1, C*H*$_2$CH$_3$), 6.58-6.61 (2H, m, *H*-C3 and *H*-C4), 6.65 (1H, dd, *J* 3.2, 1.2, *H*-A3), 6.67-6.70 (2H, m, *H*-A4), 6.79-6.81 (2H, m, *H*-A5), 7.11-7.14 (3H, m, *H*-B5, *H*-D5), 7.47-7.49 (3H, m, *H*-B6, *H*-D6), 7.64, (1H, d, *J* 1.2, *H*-C6), 7.75(2H, d, *J* 7.8, *H*-A6), 7.77-7.81 (3H, m, *H*-B4, *H*-D4), 8.09(1H, d, *J* 8.3, *H*-D3) and 8.12-8.13 (2H, m, *H*-B3);

^{13}C NMR (176 MHz, DMSO) δ_c 14.1 (CH$_2$*C*H$_3$), 40.3 (Ar*C*H$_2$C(O)), 60.0 (*C*H$_2$CH$_3$), 119.0 (*C*-D3), 119.05 and 119.07 (2 × *C*-B3), 119.57 and 119.58 (2 × *C*-A5), 122.85 and 122.87 (2 × *C*-B5 and *C*-D5), 124.1 (2 × *C*-A6), 125.09 and 125.11 (*C*-C5, *C*-C6) 129.08 and 129.09 (2 × *C*-A4), 130.3 (*C*-C4), 136.23 and 136.24 and 136.27 (2 × *C*-A3, *C*-C3), 136.87 (*C*-D4), 136.91 (*C*-B4), 143.77 and 143.78 (2 × *C*-A2), 143.9 (*C*-C2), 146.76 and 146.78 (2 × *C*-B6), 146.9 (*C*-D6), 158.6 (*C*-C1), 160.56 and 160.63 (2 × *C*-A1), 165.3 (*C*-D2), 165.52 and 165.56 (2 × *C*-B2) and 171.5 (*C*=O);

ν_{max} (ATR)/cm^{-1} 2359, 2336, 1733, 1652, 1558, 1471, 1261, 1030 and 750;

m/z: (EI+) HRMS [M+] $C_{37}H_{30}IrN_3O_2^+$: found 741.1973; calcd 741.1970.

Data for mono alkylated *fac*-Ir(ppy)$_3$ complex S3

The complex **S3** could not be assigned beyond reasonable doubt via NMR spectroscopy but is highly consistent with alkylation at the 3-postion of the phenyl ring. Further to this, attempts to perform a hydrolysis/decarboxylation/oxidation sequence to give the formyl complex failed. Under both the conditions used for **5** (*vide Infra*) and more forcing conditions (Dioxane/NaOH, 80 °C) the starting material was recovered.

^{1}H NMR (700 MHz, DMSO) δ_H 0.99 (3H, t, *J* 7.1, CH$_2$C*H*$_3$), 3.13 (1H, d, *J* 16.8, ArC*H$_A$*H$_B$C(O)) 3.27 (1H, d, *J* 16.8, ArCH$_A$*H$_B$*C(O)), 3.65-3.74 (2H, m, C*H*$_2$CH$_3$), 6.54 (1H, dd, *J* 7.6, 1.1), 6.56-6.58 (1H, m), 6.60-6.61 (1H, m), 6.69-6.72 (2H, m), 6.82-6.84 (2H,

m), 6.88 (1h, t, *J* 7.6), 7.04-7.09 (3H, m), 7.21-7.22 (2H, m), 7.25-7.26 (1H, m), 7.69-7.70 (1H, m), 7.76-7.82 (5H, m), 8.11 (2H, dd, *J* 13.4, 8.3) and 8.20 (1H, d, *J* 8.3);

^{13}C NMR (176 MHz, DMSO) δ_c 13.9 ($CH_2\mathit{C}H_3$), 44.5 (Ar$\mathit{C}H_2C(O)$), 59.1 ($\mathit{C}H_2CH_3$), 119.0, 119.2, 119.7, 119.7, 120.7, 122.3, 122.6, 122.9, 123.0, 124.1, 124.4, 128.5, 129.2, 129.9, 136.3, 136.7, 136.9, 137.1, 137.4, 142.5, 143.5, 144.1, 145.3, 146.2, 146.2, 159.4, 159.4, 165.5, 165.6, 166.3 and 172.6;

ν_{max} (ATR)/cm^{-1} 2360, 2335, 1733, 1599, 1471, 1161 and 749;

m/z: (EI+) HRMS [M+] $C_{37}H_{30}IrN_3O_2^+$: found 741.1956; calcd 741.1970.

8.0 Assignment for *fac*-Ir(ppy)$_3$ and comparison with 5

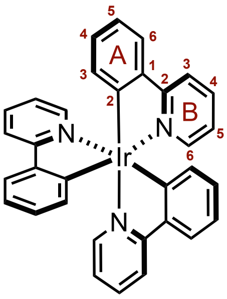

^1H NMR (700 MHz, DMSO) δ_H 6.62 (3H, d, *J* 6.8, *H*-A3), 6.64-6.66 (3H, m, *H*-A4), 6.76-6.78(3H, m, *H*-A5), 7.09-7.10 (3H, m, *H*-B5), 7.45 (3H, d, *J* 5.0, *H*-B6) 7.72 (3H, d, *J* 7.5, *H*-A6), 7.76 (3H, t, *J* 8.5, *H*-B4) and 8.10 (3H, d, *J* 8.2, *H*-B3);

^{13}C NMR (176 MHz, DMSO) δ_c 119.0 (*C*-B3), 119.6 (*C*-A5), 122.8 (*C*-B5), 124.1 (*C*-A6), 129.1 (*C*-A4), 136.3 (*C*-A3), 136.9 (*C*-B4), 143.8 (*C*-A2), 146.8 (*C*-B6), 160.7 (*C*-A1) and 165.6 (*C*-B2).

^1H NMR (700 MHz, CD_2Cl_2) δ_H 6.75 (3H, dd, *J* 7.8, 1.1, *H*-A3), 6.79 (3H, td, *J* 7.3, 1.4, *H*-A4), 6.89 (3H, ddd, *J* 7.8, 7.1, 1.4, *H*-A5), 6.93 (3H, ddd, *J* 7.2, 5.6, 1.4, *H*-B5), 7.57 (3H, ddd, *J* 5.6, 1.6, 0.80, *H*-B6) 7.65-7.68 (3H, m, *H*-A6 and *H*-B4) and 7.93 (3H, d, *J* 8.2, *H*-B3);

^{13}C NMR (176 MHz, CD_2Cl_2) δ_C 118.8 (*C*-B3), 119.8 (*C*-A5), 122.1 (*C*-B5), 124.0 (*C*-A6), 129.6 (*C*-A4), 136.2 (*C*-B4), 136.7 (*C*-A3), 143.8 (*C*-A2), 147.1 (*C*-B6), 160.9 (*C*-A1) and 166.4 (*C*-B2)

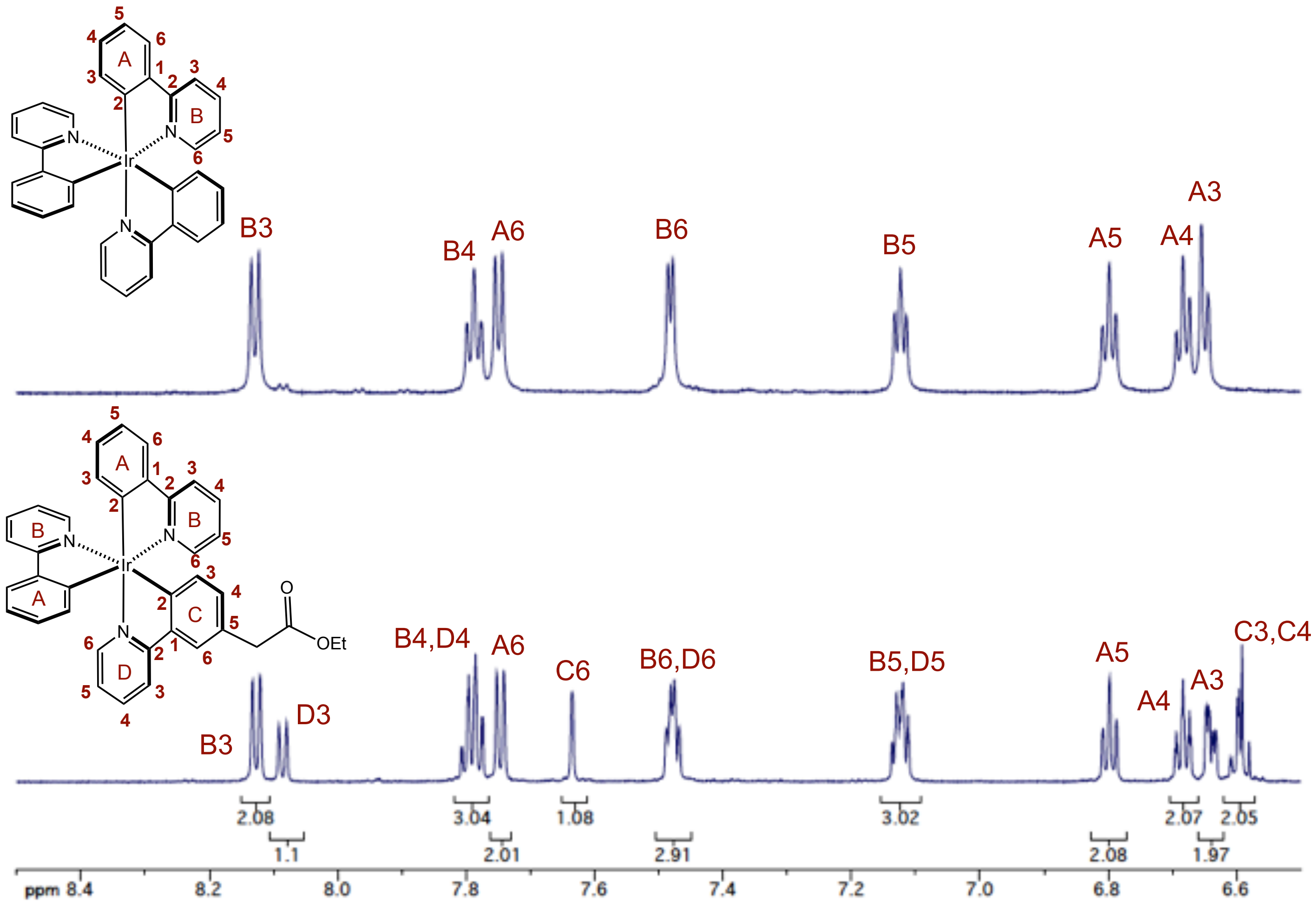

Fig. S7:Comparison of ^{1}H NMR of 1 and 5 in *D_6*-DMSO

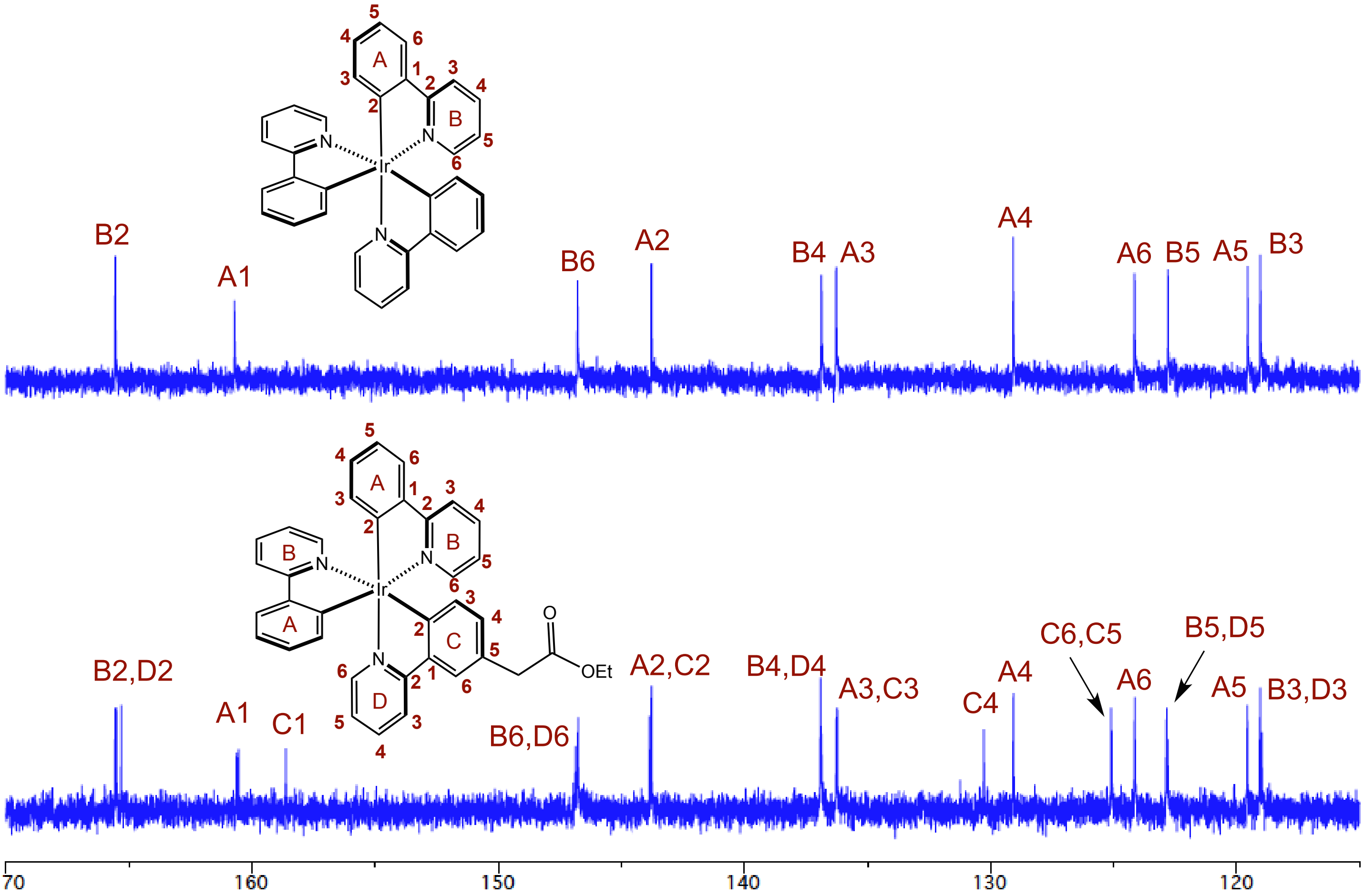

Figure S9. Comparison of ^{13}C NMR of 1 and 5 in D_6-DMSO

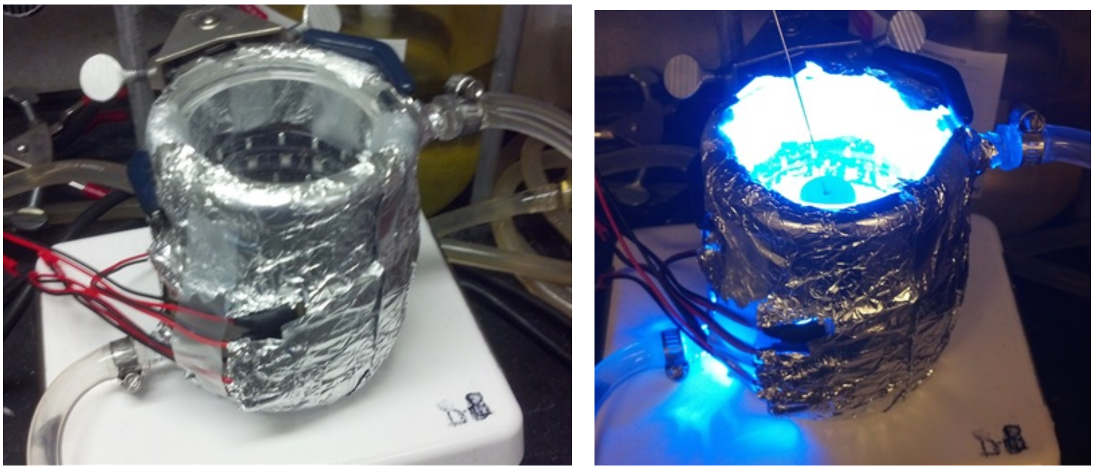

Figure S10. Reaction set up for the synthesis of 5

9.0 Transformation of 6 to to *fac*-Ir[((*p*-C(O)H)Ph)Py)(ppy)$_2$] S4

To further confirm the structural assignment of **5** it was transformed to the known aldehyde **S4** via a hydrolysis/decarboxylation/oxidation sequence.

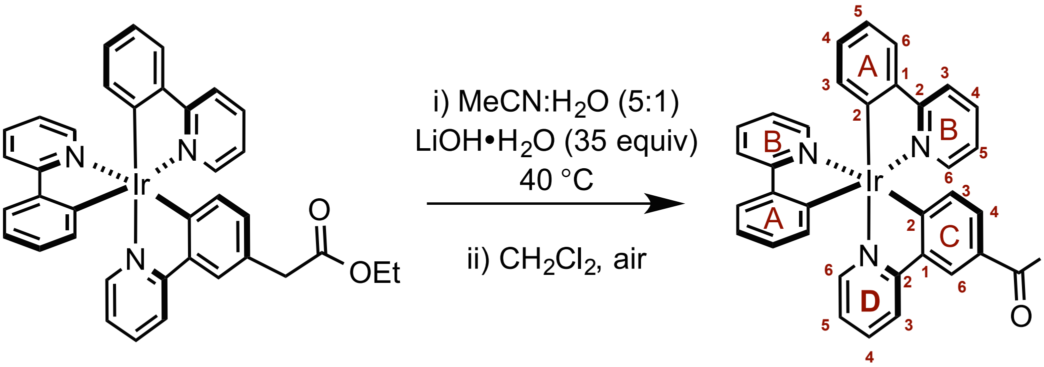

5 **S4**

To a screw cap vial was added **5** (10.0 mg, 0.013 mmol, 1.0 equiv), MeCN (2.5 ml), water (0.5 ml) and LiOH.H_2O (20.0 mg, 0.477 mmol, 35 equiv) and the solution was stirred at 40 °C for 12 h forming a bright yellow precipitate. The solvent was pipetted away from the solids and water (2 ml) was added. The slurry was transferred to a separatory funnel containing CH_2Cl_2 (10 ml). 1N HCl (10 ml) was added, the layers separated and

the aqueous solution was extracted with CH_2Cl_2 (10 ml). The combined organic portions were left to stand open to the air for 2 h. The solution was dried (Na_2SO_4), filtered and concentrated *in vacuo* to give a bright yellow crude material that was purified *via* flash column chromatography (CH_2Cl_2 to 1% MeOH: CH_2Cl_2) to give **S4** (3 mg, 34% yield). The data was inconsistent with that reported in the patent literature,[6] which appears poorly resolved e.g 6.65-6.92 (12H, m).[12a] However, the data was consistent with the major product (13% yield) isolated from the formylation of *fac*-Ir(ppy)$_3$ with $POCl_3$/DMF following the specified literature procedure[12b] (see comparison spectrum).

^{1}H NMR (700 MHz, DMSO) δ_H 6.53 (1H, d, *J* 7.5, *H*-A3), 6.65 (1*H*, d, *J* 7.1, *H*-A3), 6.70 (1H, t, *J* 7.1, *H*-A4), 6.73 (1H, td, *J* 7.4, 1.0, *H*-A4), 6.81-6.86 (2H, m, *H*-A5), 6.94 (1H, d, *J* 7.8, *H*-C3), 7.14-7.18 (3H, m, *H*-C4, *H*-B5), 7.24 (1H, t, *J* 6.5, *H*-D5), 7.46 (1H, d, *J* 5.4, *H*-B6), 7.48 (1H, d, *J* 5.5, *H*-B6), 7.55 (1H, d, *J* 5.3, *H*-D6), 7.79 (2H, t, *J* 6.5, *H*-A6), 7.83 (2H, td, *J* 7.7, 0.6, *H*-B4), 7.89 (1H, td, *J* 7.8, 1.4, *H*-D4), 8.17 (2H, d, *J* 8.2, *H*-B3), 8.27 (1H, d, J 1.5, *H*-C6), 8.27 (1H, d, *J* 8.1, *H*-D3) and 9.78 (1H, s, C(O)H);

^{13}C NMR (176 MHz, DMSO) δ_c 119.7 (*C*-B3), 119.8 (*C*-B3), 120.1 (*C*-D3), 120.5 (*C*-A5), 120.6 (*C*-A5), 123.4 and 123.5 (2 × *C*-B5 and *C*-D5), 124.3 (2 × *C*-A6), 124.8 (*C*-C6 and *C*-C5), 129.79 and 129.83 and 129.89 (2 × *C*-A4 and *C*-C4), 136.4 (*C*- A3), 136.6 (*C*-A3), 137.3 (*C*-C3), 137.7 and 137.85, and 137.90 (2 × *C*-B4 and *C*-D4) 144.1 (*C*-A2), 144.3 (*C*-A2), 145.8 (*C*-C2), 147.1 and 147.4 and 147.5 (2 × *C*-B6 and *C*-D6) 159.6 (*C*-A1), 159.9 (*C*-A1), 164.5 (*C*-C1), 165.5 (*C*-B2), 165.8 (*C*-B2), 177.2(*C*-D2) and 192.3 (*C*(O)H);

ν_{max} (ATR)/cm^{-1} 2160, 1675, 1598, 1576, 1471, 1413, 1261, 1186, 1059, 749 and 732;

m/z: (EI+) HRMS [M+] $C_{34}H_{24}IrN_3O^+$: found 684.1602; calcd 684.1621.

---

6 (a) Evans, C. E. B.; Crutchley, R. J.; Derosa, M. C.; Mosher, P. J. From PCT Int. Appl. (2004), WO 2004031321 A1 20040415. (b) Stoessel, P.; Spreitzer, H.; Becker, H. From PCT Int. Appl. (2003), WO 2003040160 A1 20030515.

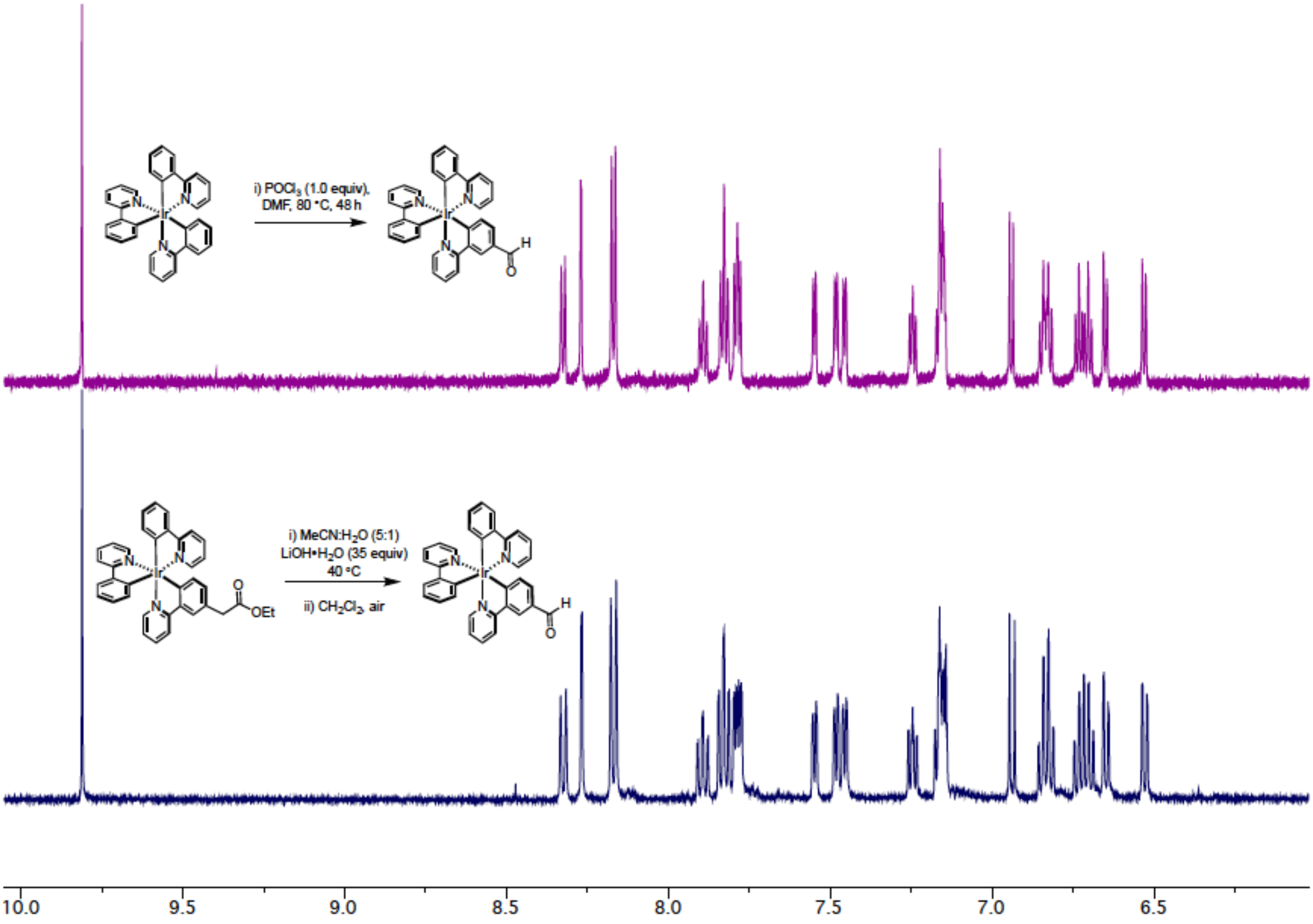

Figure S11. Comparison of ^{1}H NMR of S4 formed via formylation and via hydrolysis/decarboxylation/oxidation

10.0 ^{13}C Coordination-Induced Shift Calculations

Following the precedent of Watts[1] shifts are calculated as [CIS, δ(complex) − δ(free ligand)] in ppm and serve as an effective tool to aid and verify assignments. Calculated shifts (table S2 and Fig.S11) are similar to those reported for the heteroleptic complexes [Ir(ppy-*R*)$_2$(bpy)].

Table S2: ^{13}C Coordination-induced shifts [CIS, δ(complex) − δ(free ligand)] in ppm

Position	*fac*-Ir(ppy)$_3$ 1	*fac*-Ir(Ph(*p*-MePy))$_3$ 6	*fac*-Ir((*p*-MePh)(*p*-MePy))$_4$ 7	*fac*-Ir((*p*-CF$_3$Ph)(*p*-MePy))$_3$ 8	*fac*-Ir((*p*-CF$_3$Ph)(*p*-^{t}BuPy))$_3$ 9
	CD_2Cl_2	CD_2Cl_2	CD_2Cl_2	$CDCl_3$	$CDCl_3$
**A1**	21.3	22.4	17.7	25.8	25.4
**A2**	16.7	17.6	20.2	14.0	14.2
**A3**	7.7	8.5	8.2	7.9	8.0
**A4**	0.4	1.0	1.1	0.5	0.4
**A5**	-9.2	-8.7	-9.9	-8.8	-8.9
**A6**	-3.2	-2.6	-3.2	-1.5	-4.0
**B2**	9.0	9.4	8.9	9.1	8.7
**B3**	-1.8	-1.4	-1.9	-1.4	-1.8
**B4**	-0.9	0.2	-0.4	0.3	-0.2
**B5**	-0.4	0.4	-0.1	0.3	0.4
**B6**	-2.8	-2.3	-2.7	-3.0	-3.1

Table S2 continued: ^{13}C Coordination-induced shifts [CIS, δ(complex) − δ(free ligand)] in ppm

	fac-Ir(ppy)$_3$ 1	*fac*-Ir[((p-C(O)H)Ph)Py)(ppy)$_2$] S4
**Position**	DMSO	DMSO
**A1**	22.1	21.0
**A2**	17.3	19.1
**A3**	7.5	7.8
**A4**	0.1	0.8
**A5**	-9.2	-8.1
**A6**	-2.4	-2.2
**B2**	9.6	9.7
**B3**	-1.2	-0.4
**B4**	-0.3	0.7
**B5**	0.2	0.8
**B6**	-2.7	-2.0

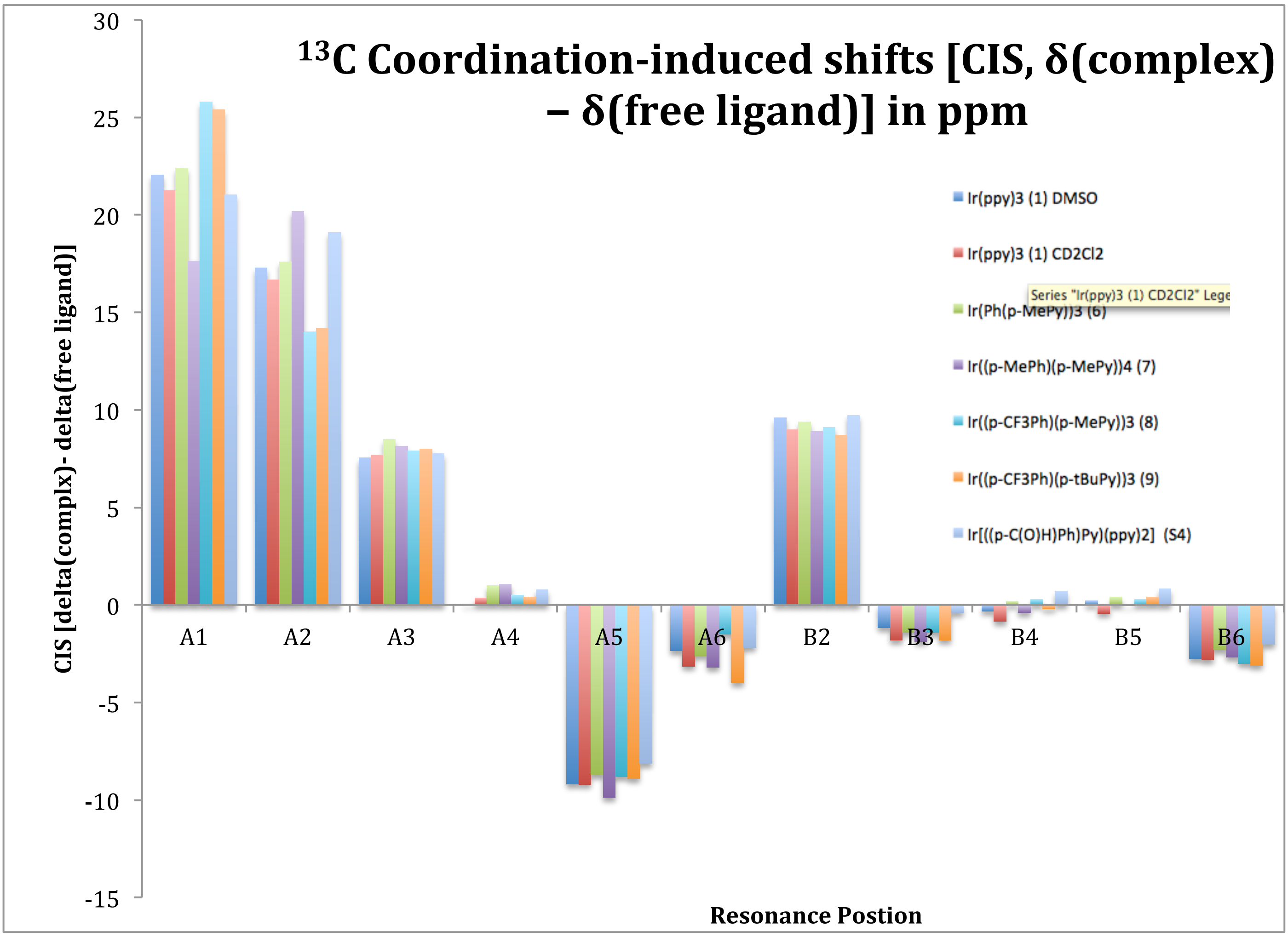

Figure S12. **^{13}C Coordination-induced shifts [CIS, δ(complex) – δ(free ligand)] in ppm**

11.0 Photocatalyst Quenching Studies

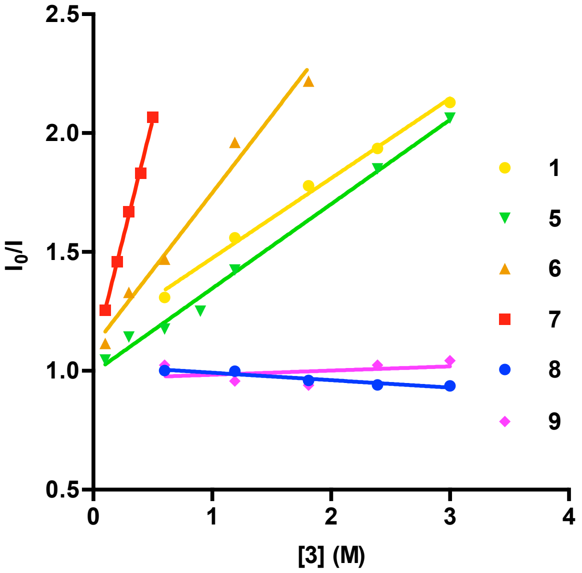

Figure S13.

All quenching data was recorded in a quartz cuvette with a stir bar at 25 °C with DMA as the solvent. Excitation was at 450 nm with emission measured at the maximum as stated. All values are the average of 3 measurements.

**Photocatalyst**		**Concentration/ M**	**Emission Maximum/nm**
Ir(ppy)$_3$	**1**	2.85E-06	520
Ir[*p*-(CH$_2$C(O)OEt)PhPy)(ppy)$_2$]	**5**	3.24E-06	516
Ir(Ph(*p*-MePy))$_3$	**6**	3.25E-06	517
Ir((*p*-MePh)(*p*-MePy))$_3$	**7**	3.61E-06	520
Ir((*p*-CF$_3$Ph)(*p*-MePy))$_3$	**8**	2.96E-06	516
Ir((*p*-CF$_3$Ph)(*p*-^{t}BuPy))$_3$	**9**	1.82E-06	490

Table S3: Photocatalyst Quenching Data

12.0 Full Page Versions of Manuscript Figures

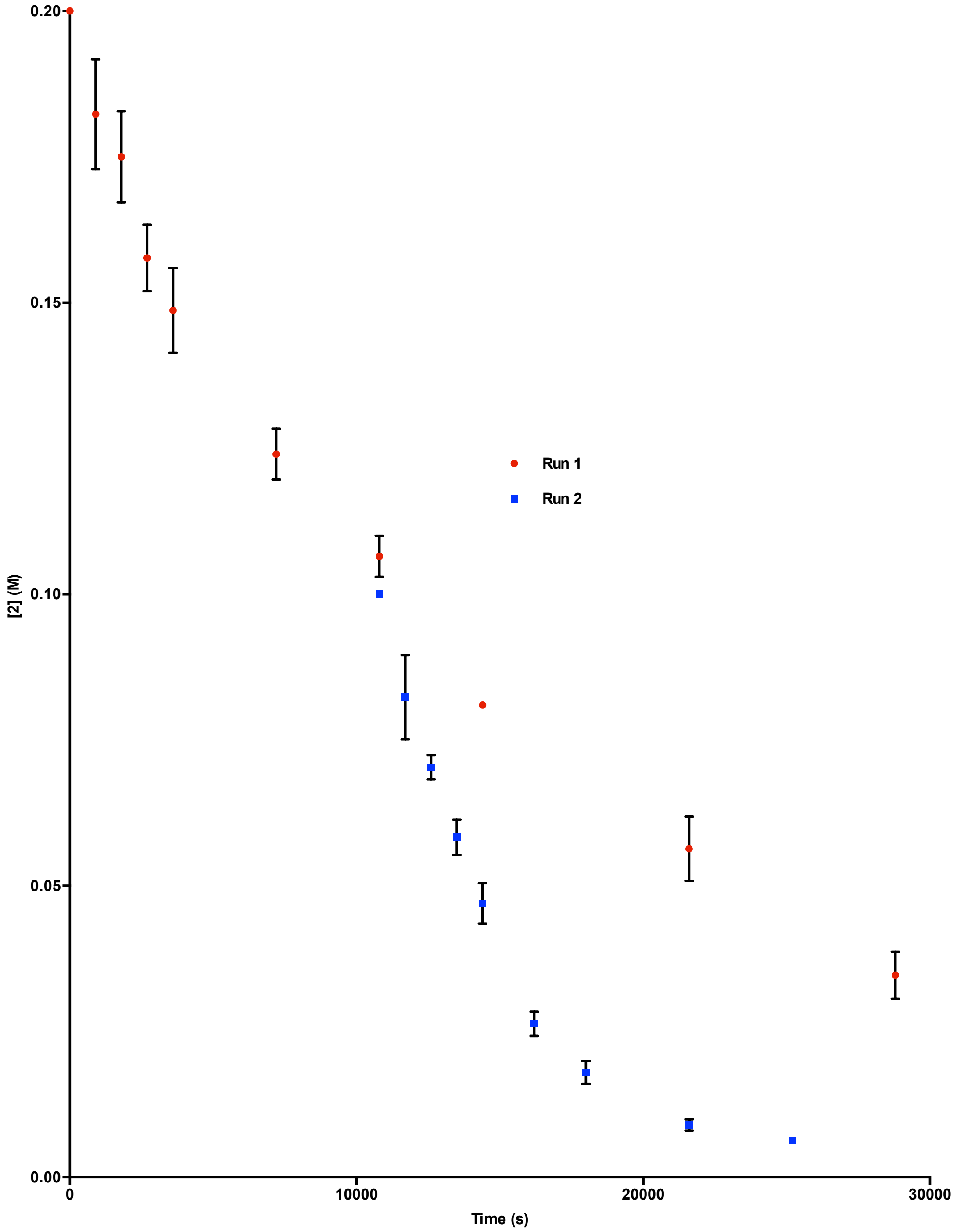

Figure S14. Profile of Run 1 as [**2**] vs time and the time-adjusted profile of Run 2 as [**2**] vs adjusted time. Run 1: **1** = 7.5 μmol. **2** = 2.00 mmol, **3** = 6.00 mmol, $NaHCO_3$ = 4.00 mmol, DMA = 10 mL. Run 2: **1** = 7.5 μmol. **2** = 1.00 mmol, **3** = 5.00 mmol, $NaHCO_3$ = 3.00 mmol, DMA = 10 mL.

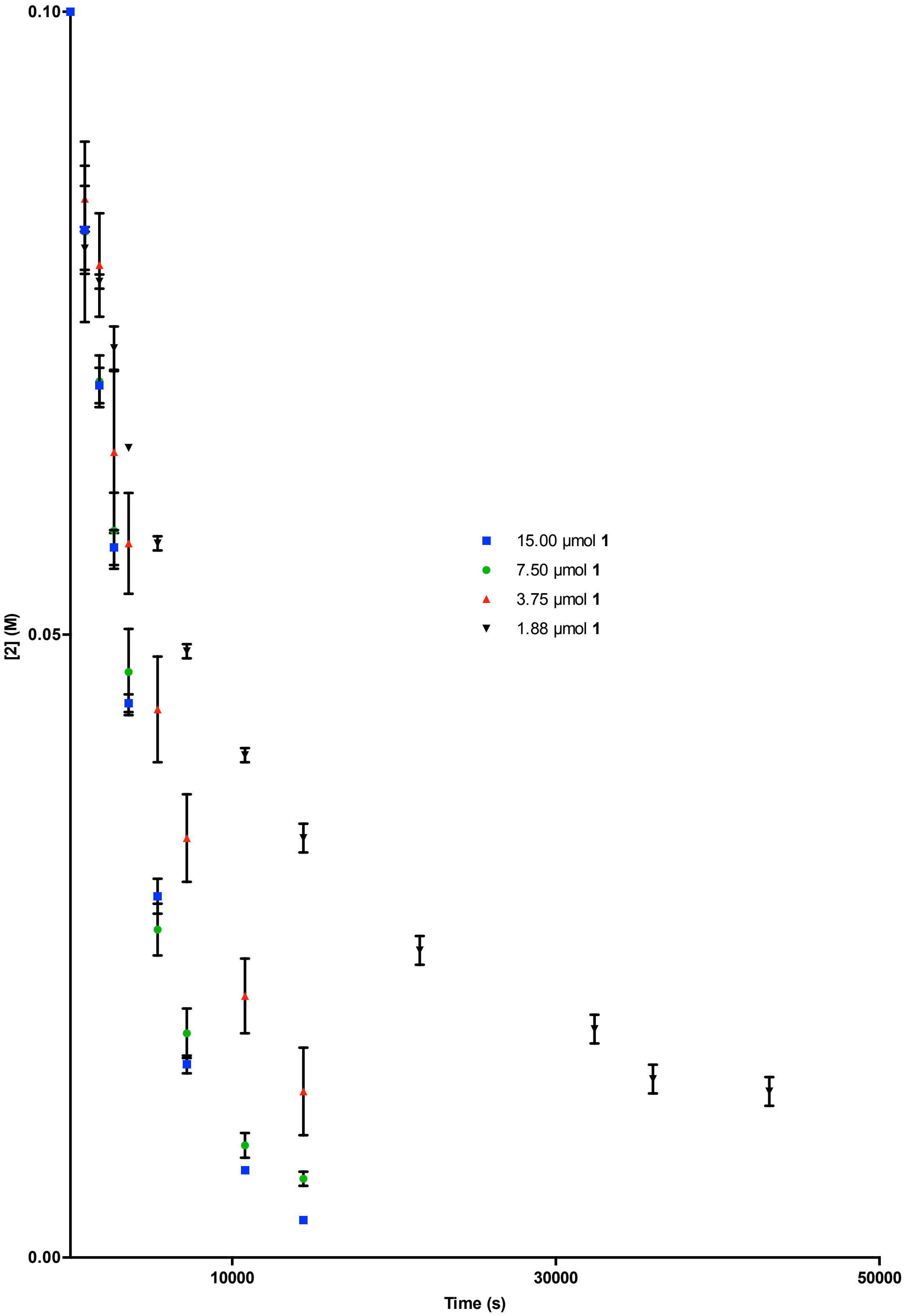

Figure S15. Effect of **1** on rate plotted as [**2**] vs time. General conditions: **2** = 1.00 mmol, **3** = 5.00 mmol, $NaHCO_3$ = 3.00 mmol, DMA = 10 mL.

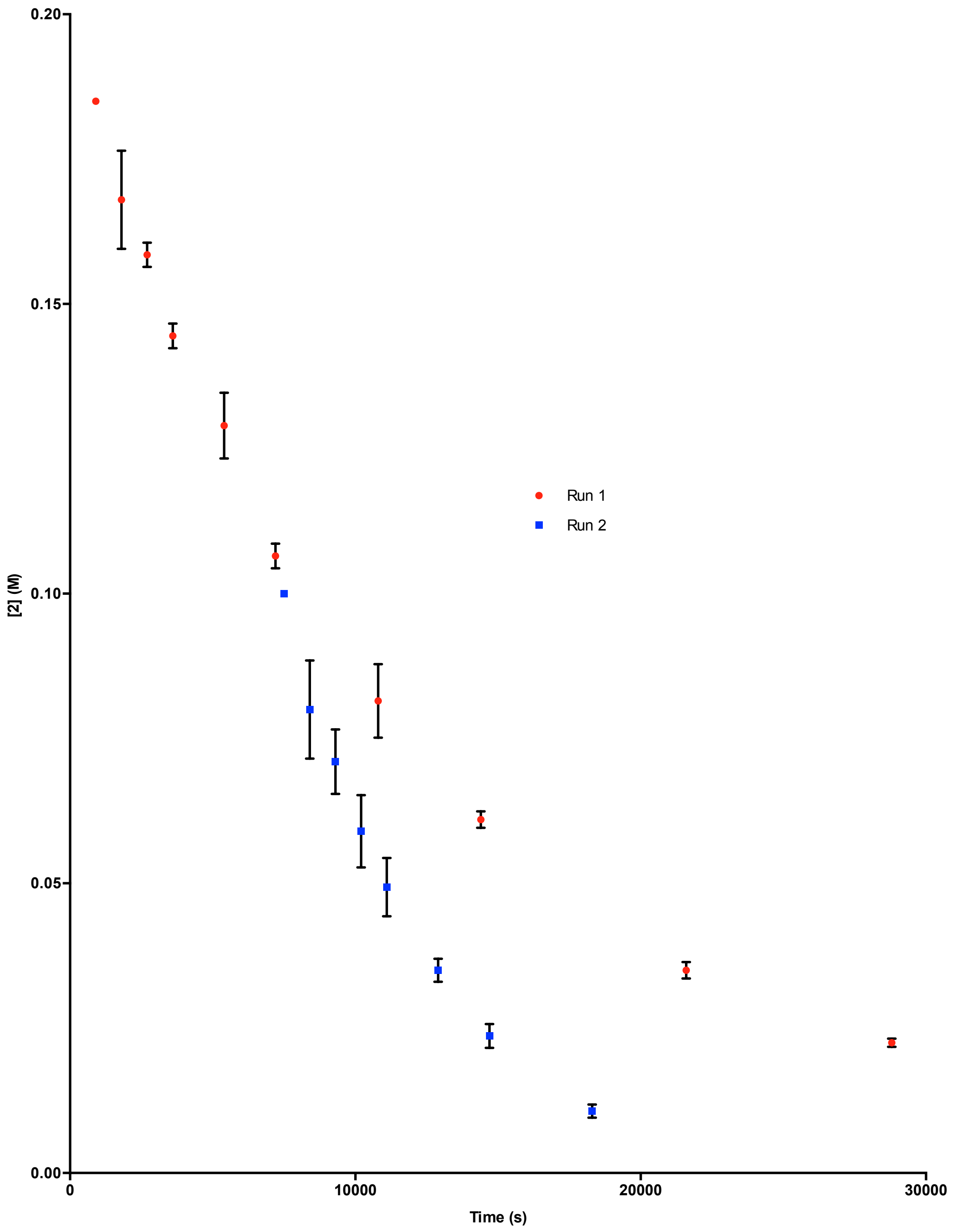

Figure S16. Profile of Run 1 as [**2**] vs time and the time-adjusted profile of Run 2 as [**2**] vs adjusted time for the **5**-catalyzed system. Run 1: **5** = 7.5 μmol. **2** = 2.00 mmol, **3** = 6.00 mmol, $NaHCO_3$ = 4.00 mmol, DMA = 10 mL. Run 2: **5** = 7.5 μmol. **2** = 1.00 mmol, **3** = 5.00 mmol, $NaHCO_3$ = 3.00 mmol, DMA = 10 mL.

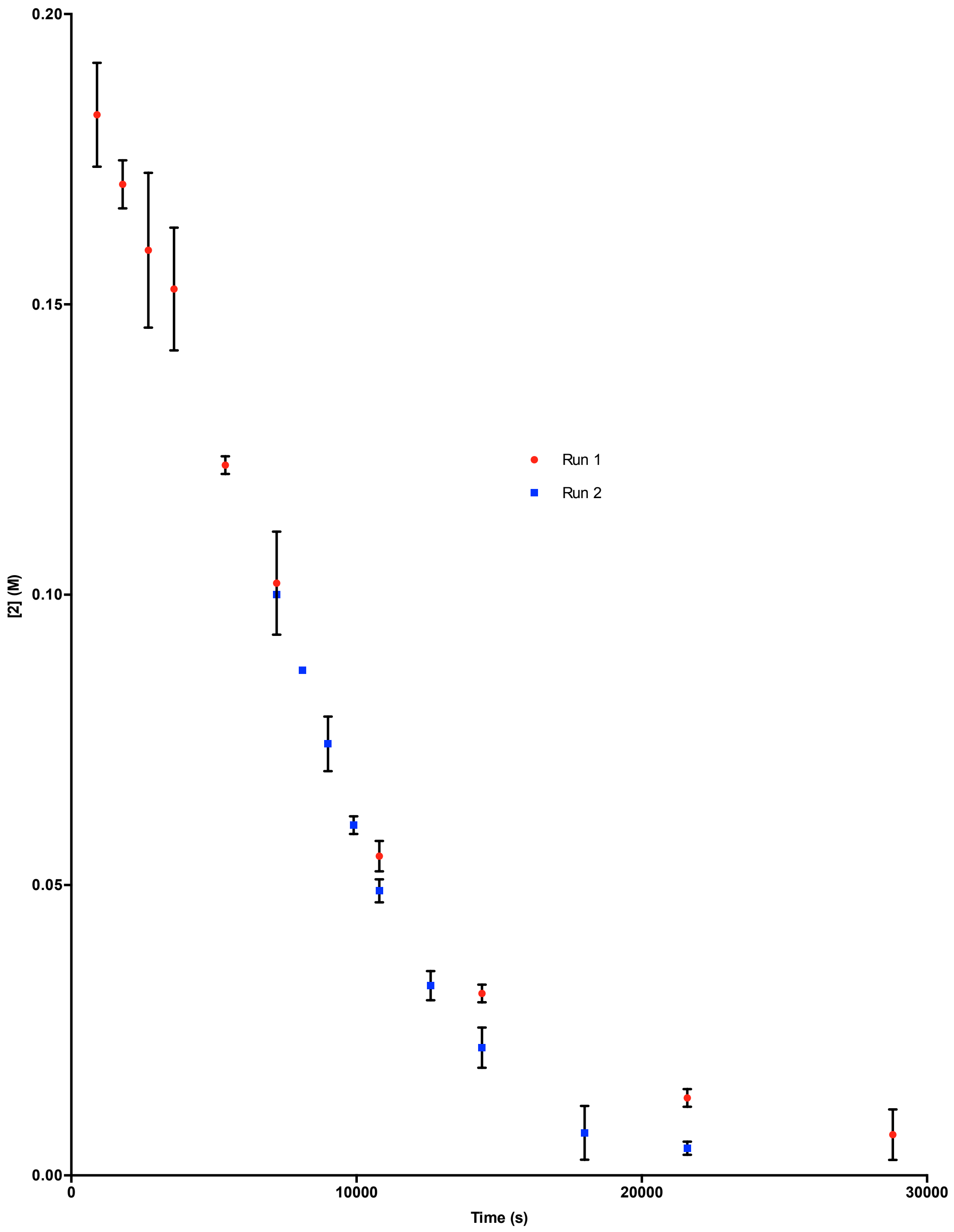

Figure S17. Figure 4. Profile of Run 1 as [**2**] vs time and the time-adjusted profile of Run 2 as [**2**] vs adjusted time for the **2**-catalyzed reaction at 2 mol% catalyst. Run 1: **1** = 40.0 μmol, **2** = 2.00 mmol, **3** = 6.00 mmol, $NaHCO_3$ = 4.00 mmol, DMA = 10 mL. Run 2: **1** = 40.0 μmol. **2** = 1.00 mmol, **3** = 5.00 mmol, $NaHCO_3$ = 3.00 mmol, DMA = 10 mL.

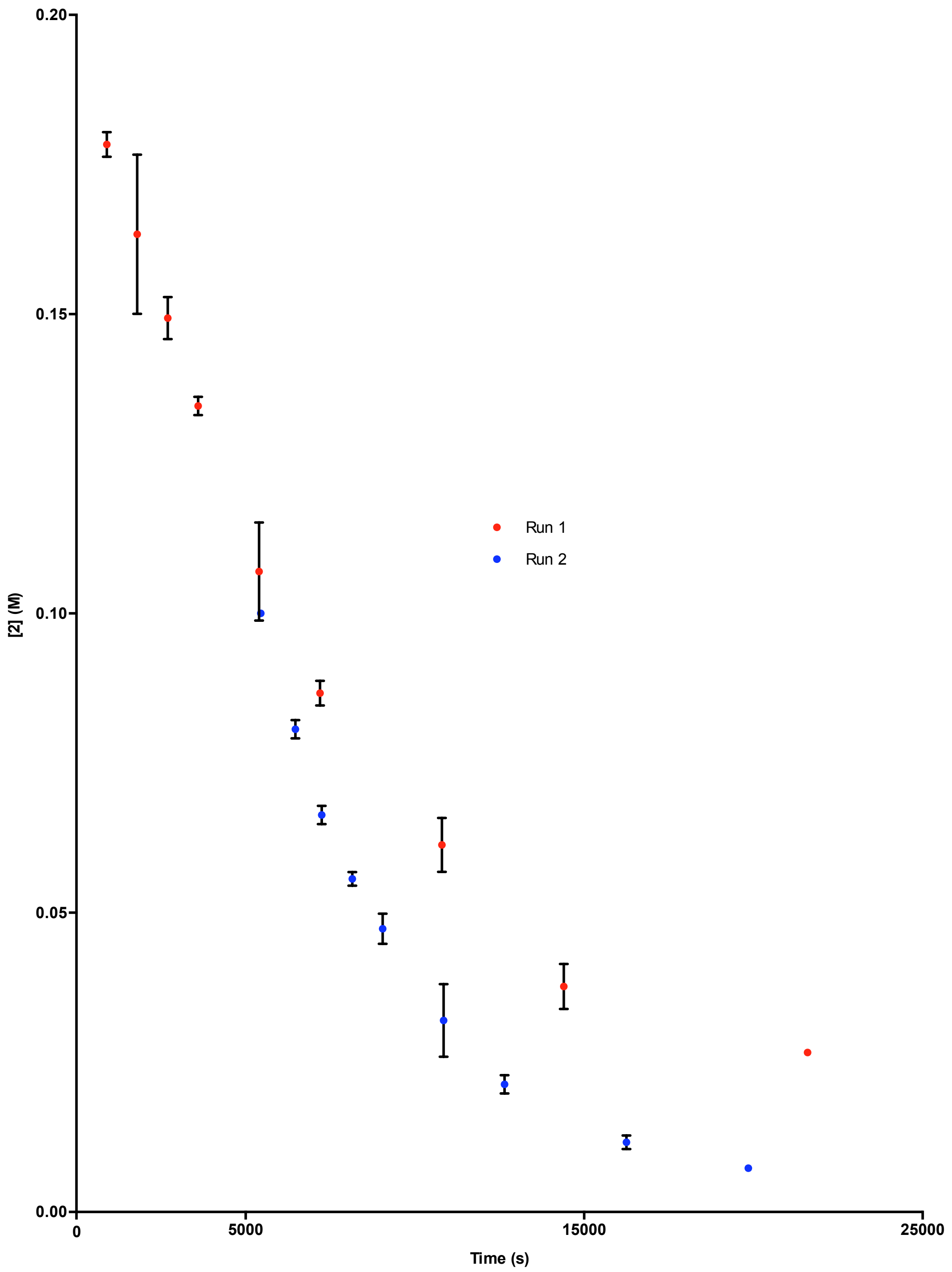

Figure S18. Profile of Run 1 as [**2**] vs time and the time-adjusted profile of Run 2 as [**2**] vs adjusted time for the **6**-catalyzed system. Run 1: **6** = 7.5 μmol. **2** = 2.00 mmol, **3** = 6.00 mmol, $NaHCO_3$ = 4.00 mmol, DMA = 10 mL. Run 2: **6** = 7.5 μmol. **2** = 1.00 mmol, **3** = 5.00 mmol, $NaHCO_3$ = 3.00 mmol, DMA = 10 mL.

13.0 MS Data

Intractable mixture resulting from irradiation of **1** and **3** by positive ion electrospray:

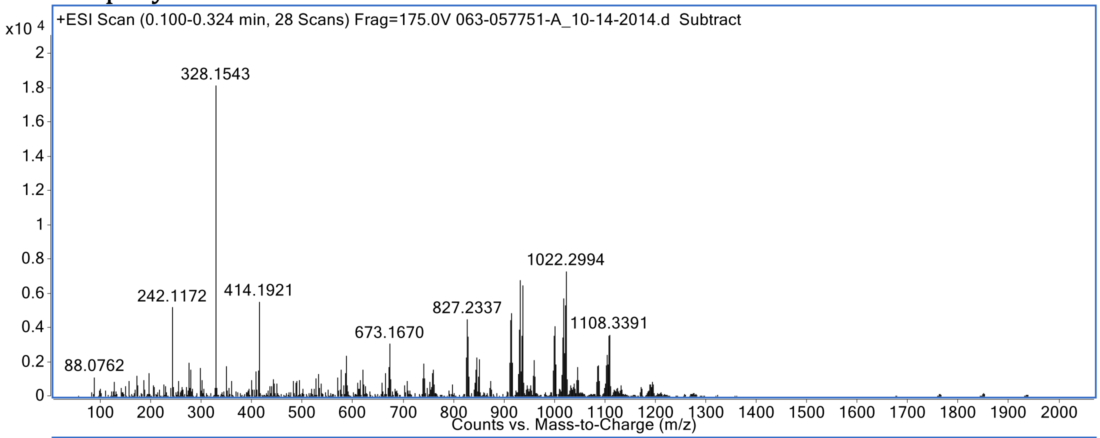

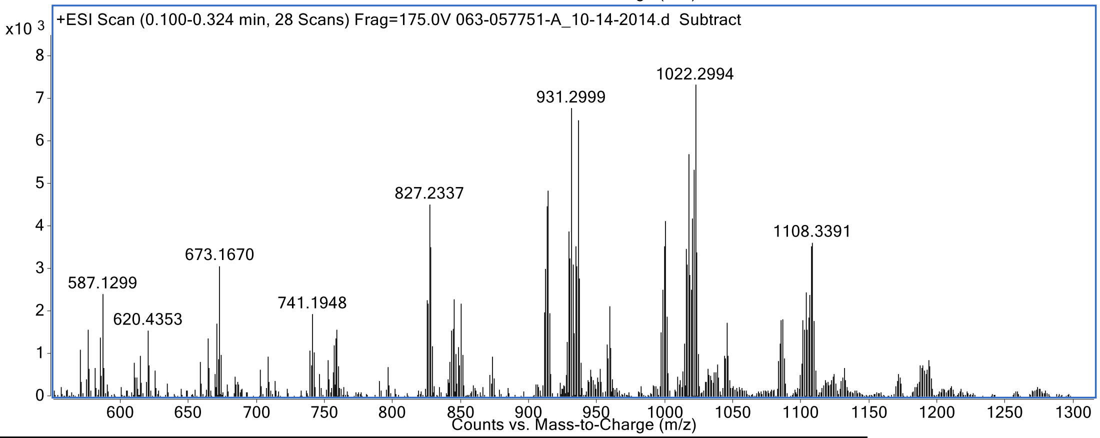

m/z	Abund	Abund %
81.9379	260.71	1.44
88.0762	1139.03	6.27
97.9682	343.78	1.89
98.9624	477.47	2.63
99.971	295.1	1.63
110.0575	355.81	1.96
122.9638	319.29	1.76
124.9642	294.4	1.62
128.0702	837.96	4.62
130.0855	295.41	1.63
141.9585	524.42	2.89
143.0318	259.73	1.43
144.0098	232.93	1.28
149.023	630.51	3.47
156.0792	931.13	5.13
168.0805	420	2.31
172.0276	1223.91	6.74
174.1117	663.41	3.65
186.2213	955.67	5.26
187.9993	427.43	2.35
196.0938	1363.81	7.51

204.1221	651.37	3.59
207.0885	569.87	3.14
214.0862	266.86	1.47
216.1951	386.51	2.13
226.0964	683.86	3.77
226.1788	465.08	2.56
228.1958	626.79	3.45
229.0749	322.92	1.78
230.2467	259.74	1.43
240.0153	489.49	2.7
242.1172	5193.39	28.61
242.2835	414.47	2.28
243.1206	988.39	5.44
244.19	562.61	3.1
250.177	241.54	1.33
252.9729	295.93	1.63
254.1145	886.89	4.88
258.2775	231.69	1.28
259.0998	355.97	1.96
261.1302	580.65	3.2
264.0989	381.14	2.1
270.096	544.51	3
274.2738	362.19	1.99
276.1438	1967.96	10.84
277.1216	489.74	2.7
279.0933	1551.19	8.54
279.1563	434.44	2.39
280.1141	208.67	1.15
280.1264	298.91	1.65
282.1128	456.16	2.51
283.1595	451.67	2.49
290.1584	221.02	1.22
298.1258	1012.78	5.58
298.3463	1683.77	9.27
299.3496	348.92	1.92
301.0744	365.93	2.02
301.1404	955.21	5.26
302.144	226.19	1.25
305.1574	478.13	2.63
309.139	214.92	1.18
318.2994	203.31	1.12
327.0842	526.72	2.9
327.1764	396.17	2.18
328.1543	18155.5	100
329.1575	3697.89	20.37
330.1603	488.85	2.69
340.1462	257.44	1.42

344.1488	299.84	1.65
344.2254	218.5	1.2
349.1806	359.38	1.98
350.1365	1773.99	9.77
350.3248	240.43	1.32
351.1399	356.82	1.97
355.2804	514.29	2.83
359.2034	925.76	5.1
368.15	286.01	1.58
371.3126	246.45	1.36
388.2521	258.41	1.42
391.2818	267.04	1.47
393.2112	260.29	1.43
393.2949	374.22	2.06
394.3501	470.01	2.59
399.3074	946.41	5.21
400.3091	394.64	2.17
405.2604	421.8	2.32
408.3089	1491.26	8.21
409.3117	383.36	2.11
413.2109	1508.39	8.31
414.1921	5536.02	30.49
415.1945	1435.11	7.9
416.1977	209.82	1.16
429.3178	219.07	1.21
431.3824	365.36	2.01
432.2802	219.92	1.21
436.1739	628.68	3.46
438.3788	618.17	3.4
443.3331	1008.99	5.56
444.3328	769.19	4.24
449.2869	748.98	4.13
468.3875	206.35	1.14
473.3509	227.93	1.26
476.3074	237.07	1.31
482.4033	889.84	4.9
483.4057	221.3	1.22
487.3601	803.47	4.43
488.3584	896.73	4.94
489.3608	275.85	1.52
493.3126	948.88	5.23
494.3176	253.55	1.4
500.2303	452	2.49
501.0918	233.96	1.29
512.4159	222.35	1.22
517.3635	209.76	1.16
518.3684	461.44	2.54

523.3224	458.1	2.52
526.43	1060.52	5.84
527.4316	325.59	1.79
531.3846	555.51	3.06
532.3834	1341.07	7.39
533.3863	487.08	2.68
537.3389	748.49	4.12
546.3974	276.46	1.52
550.6273	391.67	2.16
556.4426	223.34	1.23
570.4562	1093.6	6.02
571.4579	347.15	1.91
575.41	415.75	2.29
576.41	1568.51	8.64
577.4125	643.56	3.54
581.3649	668.5	3.68
582.3695	213.96	1.18
585.1268	1383.23	7.62
586.1321	491.61	2.71
587.1299	2402.99	13.24
588.1323	678.5	3.74
590.4214	285.32	1.57
600.4677	233.25	1.28
610.1831	802.91	4.42
611.1833	457.31	2.52
612.1776	455.21	2.51
614.4822	963.35	5.31
615.4847	324.94	1.79
619.4373	348.03	1.92
620.4353	1546.25	8.52
621.4374	725.13	3.99
625.3909	609.62	3.36
634.447	306.55	1.69
658.5083	815.92	4.49
659.5114	311.71	1.72
664.4613	1377.32	7.59
665.4632	681.21	3.75
669.4178	527.01	2.9
670.1778	224.11	1.23
671.1642	1714.44	9.44
672.1737	885.66	4.88
673.167	3069.53	16.91
674.1697	988.86	5.45
678.4729	283.27	1.56
684.2004	463.71	2.55
685.2008	279.27	1.54
686.1921	346.88	1.91

687.1817	284.79	1.57
702.5343	642.54	3.54
703.5375	241.45	1.33
708.4931	935.02	5.15
709.4955	352.22	1.94
713.4446	367.78	2.03
739.1911	1078.8	5.94
740.1942	740.81	4.08
741.1948	1933.44	10.65
742.1979	1047.31	5.77
743.2004	226.87	1.25
746.5596	525.54	2.89
747.5629	207.72	1.14
752.5137	864.88	4.76
753.5178	412.33	2.27
755.1886	213.58	1.18
756.2099	579.63	3.19
757.2001	1198.74	6.6
757.4688	281.51	1.55
758.2144	1362.03	7.5
759.2074	1564.76	8.62
760.209	714.9	3.94
761.208	207.74	1.14
764.1851	225.3	1.24
790.5848	361.79	1.99
796.5407	691.78	3.81
797.5442	271.65	1.5
825.23	2273.67	12.52
826.233	2191.27	12.07
827.2337	4513.13	24.86
828.2371	3516.57	19.37
829.241	1174.84	6.47
830.2424	238.68	1.31
834.6137	217.1	1.2
840.5661	398.1	2.19
841.2314	333.04	1.83
842.2387	814.8	4.49
843.2543	1545.1	8.51
844.25	1593.15	8.78
845.2614	2280.01	12.56
846.2656	1003.24	5.53
847.2638	304.96	1.68
848.2222	1172.16	6.46
849.2262	736.24	4.06
850.2231	2175.57	11.98
851.2256	986.97	5.44
852.2295	258.45	1.42

859.2613	273.97	1.51
860.2586	204.56	1.13
866.2107	226.15	1.25
871.2923	510.22	2.81
872.2945	306.69	1.69
873.2946	934.79	5.15
874.2943	421.46	2.32
884.5931	206.31	1.14
905.1494	286.2	1.58
906.1504	284.18	1.57
907.1492	229.45	1.26
910.264	268.6	1.48
911.2733	1976.6	10.89
912.2748	3009.64	16.58
913.2769	4476.42	24.66
914.2773	4842.66	26.67
915.2796	1958.59	10.79
916.28	524.94	2.89
923.1669	333.34	1.84
925.2552	271.66	1.5
926.2608	236.7	1.3
927.2741	512.24	2.82
928.1294	304.32	1.68
928.2735	1291.66	7.11
929.2956	3881.25	21.38
930.13	216.94	1.19
930.2904	3252.03	17.91
931.2999	6774.99	37.32
932.3039	3105.36	17.1
933.2895	1482.09	8.16
934.2588	3529.2	19.44
935.2657	3057.77	16.84
936.2597	6486.3	35.73
937.2611	2785.67	15.34
938.263	798.18	4.4
943.2973	388.83	2.14
944.2899	348.17	1.92
945.3068	637.02	3.51
946.2949	477.55	2.63
947.2892	382.87	2.11
948.2825	287.91	1.59
950.2498	443.01	2.44
951.2454	355.35	1.96
952.2497	661.5	3.64
953.2465	356.41	1.96
957.3281	1217.02	6.7
958.3346	907.58	5

959.3319	2112.94	11.64
960.3328	1142.95	6.3
961.3231	406.36	2.24
962.2661	205.83	1.13
964.2542	212.29	1.17
982.293	250.52	1.38
991.1998	268.68	1.48
992.2013	240.43	1.32
993.2011	239.33	1.32
996.3185	245.75	1.35
997.3203	1501.08	8.27
998.3157	2519.35	13.88
999.3216	3539.64	19.5
1000.3175	4127.04	22.73
1001.318	1868.39	10.29
1002.32	556.57	3.07
1009.2096	471.25	2.6
1010.2129	277.14	1.53
1011.2094	391.26	2.16
1011.3009	215.67	1.19
1012.2065	226.69	1.25
1013.1833	306.24	1.69
1013.3132	588.25	3.24
1014.1737	338.38	1.86
1014.3107	1244.84	6.86
1015.1803	270.33	1.49
1015.3315	3466.26	19.09
1016.3264	3098.96	17.07
1017.3358	5698.03	31.38
1018.3378	2860.51	15.76
1019.318	2519.5	13.88
1020.3001	4174.88	23
1021.3065	5336.39	29.39
1022.2994	7316.81	40.3
1023.3007	3383.45	18.64
1024.3009	994.85	5.48
1025.303	252.26	1.39
1029.3307	349.06	1.92
1030.3264	348.21	1.92
1031.3399	647.21	3.56
1032.3297	518.84	2.86
1033.3296	483.34	2.66
1034.3194	382.74	2.11
1035.3086	325.64	1.79
1036.2928	563.57	3.1
1037.2911	562.15	3.1
1038.2879	760.97	4.19

1039.2873	448.12	2.47
1042.3728	210.19	1.16
1043.3656	961.56	5.3
1044.3742	905.78	4.99
1045.3684	1740.37	9.59
1046.3699	965.13	5.32
1047.3529	379.5	2.09
1048.3085	210.23	1.16
1050.2934	239.78	1.32
1052.2895	211.74	1.17
1054.2911	231.23	1.27
1056.2997	210.38	1.16
1083.361	844.45	4.65
1084.3581	1237.78	6.82
1085.3624	1793.46	9.88
1086.3567	1818.59	10.02
1087.3597	891.04	4.91
1088.3589	303.6	1.67
1097.3334	212.05	1.17
1099.2209	286.72	1.58
1099.3539	501.77	2.76
1100.3528	768.29	4.23
1101.3663	1799.4	9.91
1102.3648	1569.19	8.64
1103.3722	2443.62	13.46
1104.3735	1567.28	8.63
1105.3529	1858.94	10.24
1106.3431	2392.49	13.18
1107.3458	3525.34	19.42
1108.3391	3616.4	19.92
1109.3412	1776.11	9.78
1110.344	615.26	3.39
1115.3614	205.51	1.13
1116.3654	261.78	1.44
1117.3679	381.7	2.1
1118.3656	336.24	1.85
1119.3703	341.21	1.88
1120.356	270.08	1.49
1121.3464	293.64	1.62
1122.3346	391.22	2.15
1123.3376	473.05	2.61
1124.3315	523.42	2.88
1125.334	298.52	1.64
1129.4027	372.52	2.05
1130.4118	451.53	2.49
1131.4053	665.48	3.67
1132.4031	396.52	2.18

1133.3742	220.21	1.21
1169.3958	243.61	1.34
1170.4008	357.58	1.97
1171.4011	539.48	2.97
1172.3918	440.75	2.43
1173.4007	297.7	1.64
1183.3944	219.57	1.21
1184.3969	224.43	1.24
1185.3943	300.25	1.65
1186.3978	352.04	1.94
1187.4017	724.85	3.99
1188.4061	668.22	3.68
1189.4107	726.79	4
1190.4099	606.94	3.34
1191.3934	533.89	2.94
1192.388	624.8	3.44
1193.3856	854.8	4.71
1194.3831	718.09	3.96
1195.3858	432.45	2.38
1209.3841	207.22	1.14
1210.3743	243.24	1.34
1273.4415	226.98	1.25

Intractable mixture resulting from irradiation of **5** and **3** by positive ion electrospray:

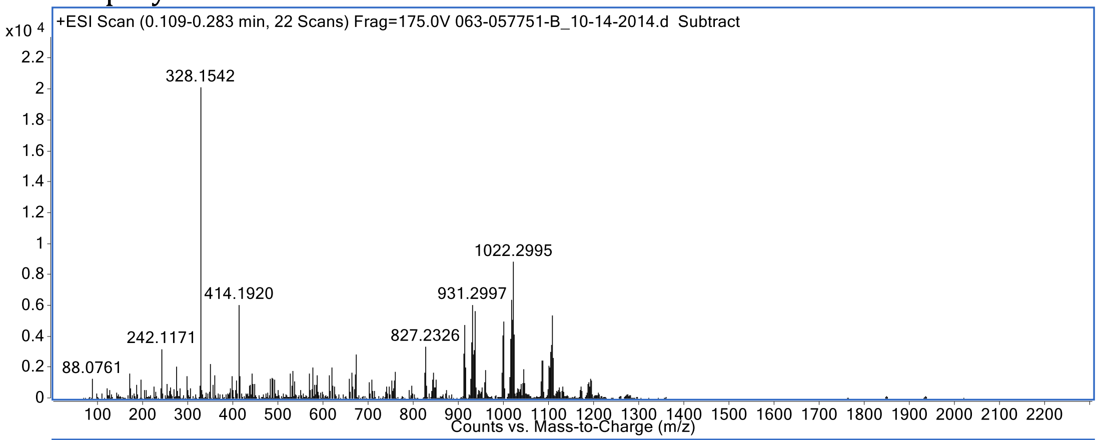

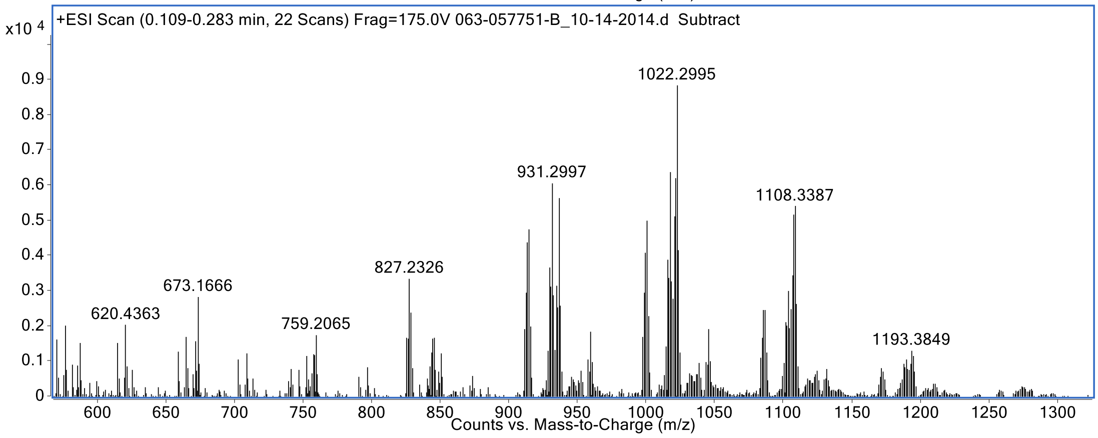

m/z	Abund	Abund %	
88.0761	1265.35	6.28	
172.0276	1632.9	8.11	
196.0937	1240.17	6.16	
242.1171	3206.42	15.92	
276.144	2046.28	10.16	
298.1257	1433.42	7.12	
298.3461	1240.92	6.16	
328.1542	20138.53	100	
329.1575	4013.59	19.93	
350.1364	2217.66	11.01	
359.2033	1486.74	7.38	
399.3074	1467.95	7.29	
408.3082	1164.16	5.78	
413.2116	2286.92	11.36	
414.192	6061.92	30.1	
415.195	1479.46	7.35	
443.3336	1600.43	7.95	
482.4045	1273.16	6.32	
487.3591	1369.45	6.8	
488.3584	1284.62	6.38	
493.3133	1250.2	6.21	
526.4302	1627.63	8.08	
532.3839	1774.23	8.81	
537.3396	1111.9	5.52	
570.4568	1628.04	8.08	
576.4101	2005.33	9.96	
587.1303	1531.78	7.61	
614.483	1516.11	7.53	
620.4363	2033.1	10.1	
658.5078	1272.64	6.32	
664.4613	1709.26	8.49	
671.1641	1571.58	7.8	
673.1666	2831.12	14.06	
702.5339	1050.49	5.22	
708.4915	1239.94	6.16	
752.5144	1156.09	5.74	
757.1998	1200.45	5.96	
758.2132	1182.93	5.87	
759.2065	1750.42	8.69	
825.2298	1673.49	8.31	
826.2328	1643.11	8.16	
827.2326	3349.08	16.63	
828.2369	2389.39	11.86	
843.2495	1245.4	6.18	
844.248	1651.64	8.2	
845.2584	1671.01	8.3	

850.2217	1225.84	6.09	
911.271	1923.82	9.55	
912.2731	2941.74	14.61	
913.2761	4373.18	21.72	
914.2764	4747.84	23.58	
915.2791	1998.64	9.92	
928.2716	1293.19	6.42	
929.2943	3667.24	18.21	
930.2885	3129.54	15.54	
931.2997	6062.93	30.11	
932.3024	2871.08	14.26	
933.2905	1324.69	6.58	
934.2582	3139.23	15.59	
935.2654	2527.22	12.55	
936.2596	5640.16	28.01	
937.2609	2571.95	12.77	
957.3283	1066.96	5.3	
959.3307	1834.59	9.11	
997.3202	1703.98	8.46	
998.315	2945.01	14.62	
999.3208	4094.69	20.33	
1000.317	5005.06	24.85	
1001.3182	2280.63	11.32	
1014.3092	1426.08	7.08	
1015.3304	3893.45	19.33	
1016.3254	3373.99	16.75	
1017.335	6383.63	31.7	
1018.3385	3280.94	16.29	
1019.3175	2782.13	13.81	
1020.2996	5119.06	25.42	
1021.306	6209.89	30.84	
1022.2995	8841.23	43.9	
1023.2998	4150.86	20.61	
1024.3019	1253.48	6.22	
1043.3661	988.59	4.91	
1045.3688	1927.27	9.57	
1046.3668	1019.97	5.06	
1083.3604	1099.41	5.46	
1084.3569	1674.52	8.32	
1085.3617	2465.35	12.24	
1086.3569	2467.42	12.25	
1087.3597	1265.67	6.28	
1101.3669	2123.41	10.54	
1102.3638	2010.16	9.98	
1103.3713	2999.05	14.89	
1104.3736	1943.35	9.65	
1105.3527	2490.67	12.37	

1106.342	3452.06	17.14	
1107.3456	5171.77	25.68	
1108.3387	5401.08	26.82	
1109.3403	2635.81	13.09	
1189.41	1067.88	5.3	
1193.3849	1302.8	6.47	
1194.3803	1151.42	5.72	

14.0 NMR Spectra

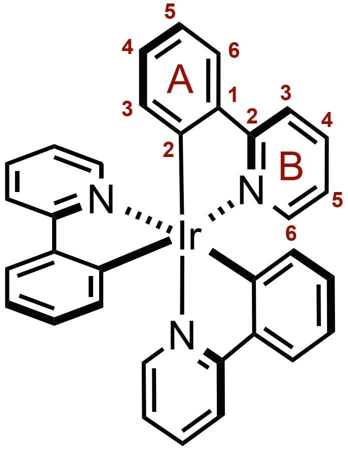

1, ^{1}H, 700 MHz, CD_2Cl_2

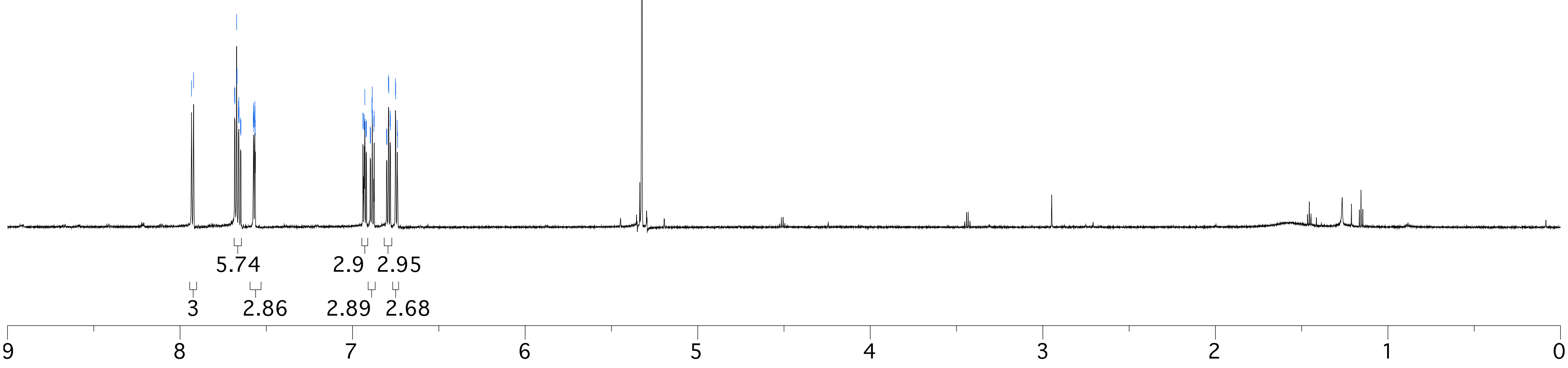

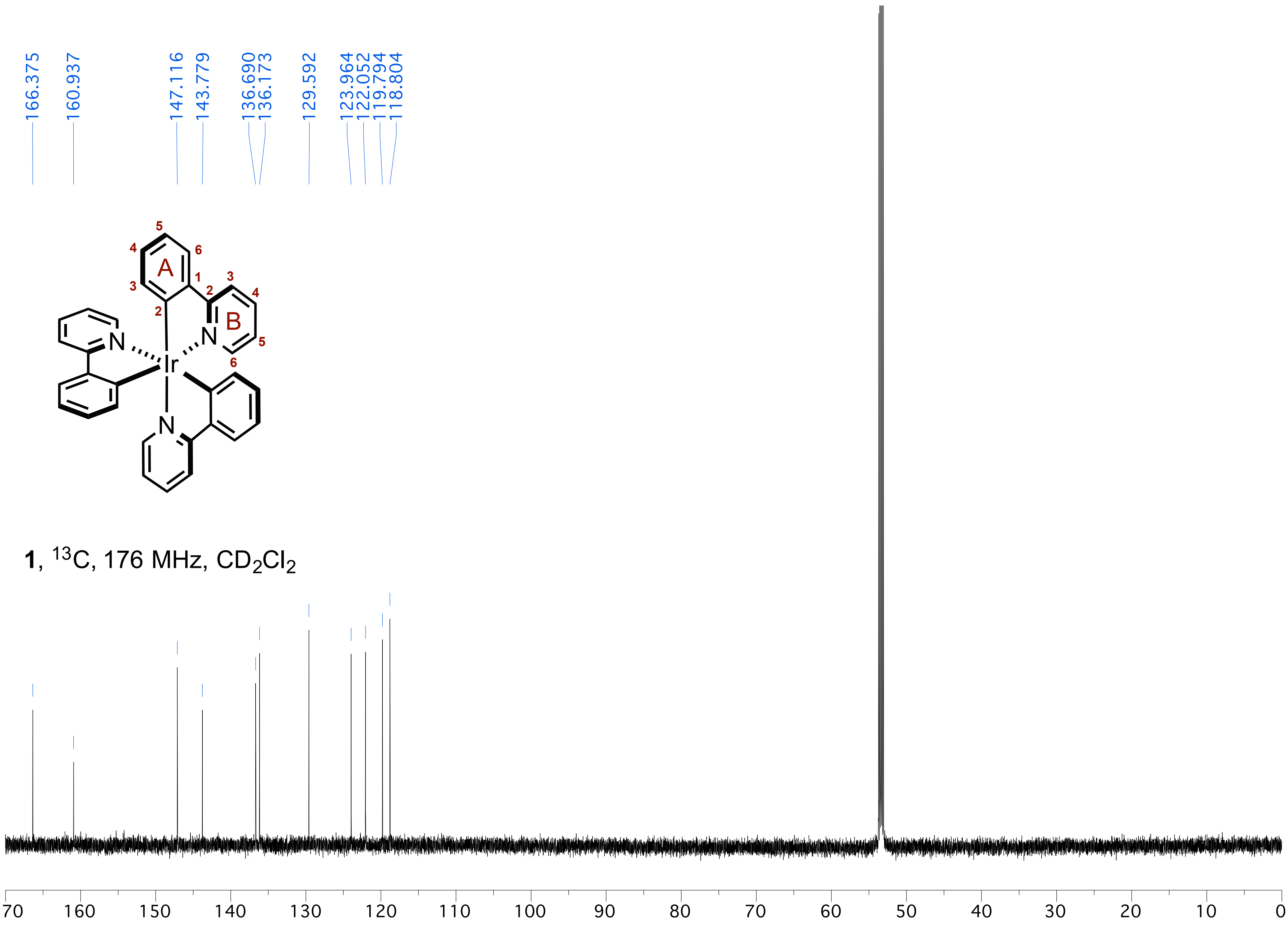

1, ^{13}C, 176 MHz, CD_2Cl_2

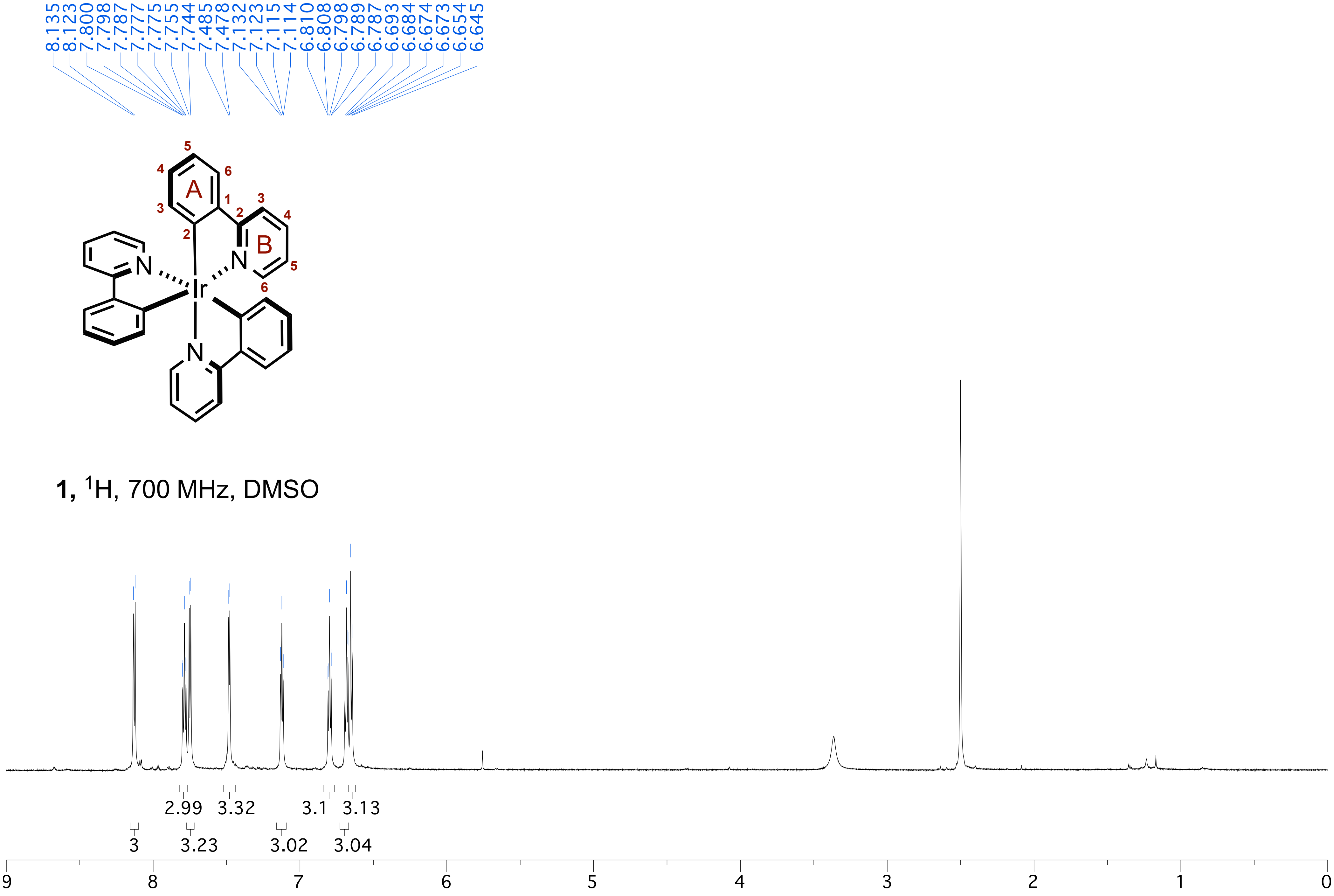

1, ^{1}H, 700 MHz, DMSO

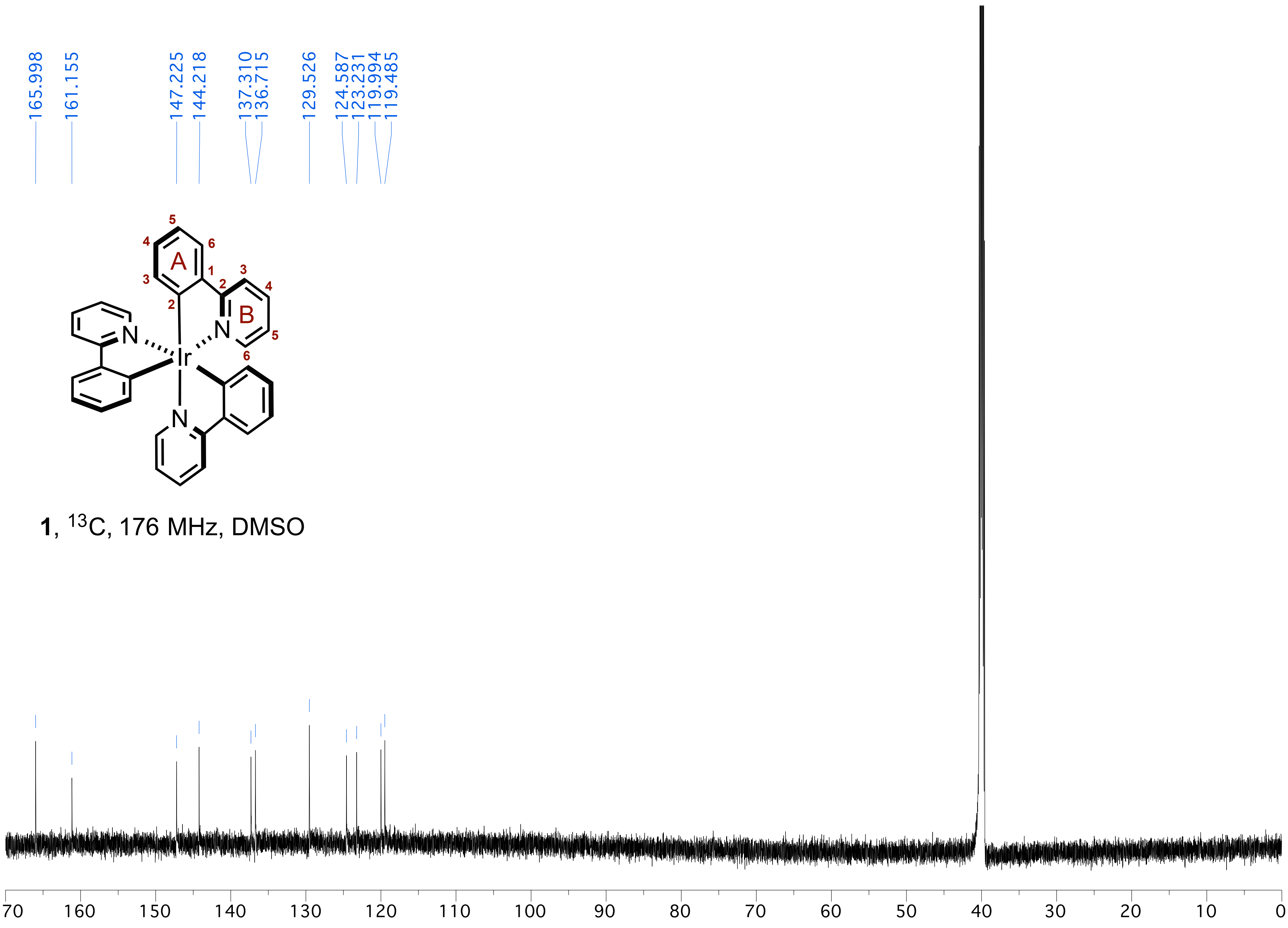
165.998
161.155
147.225
144.218
137.310
136.715
129.526
124.587
123.231
119.994
119.485
A
B
N
Ir
1, ^{13}C, 176 MHz, DMSO
170 160 150 140 130 120 110 100 90 80 70 60 50 40 30 20 10 0

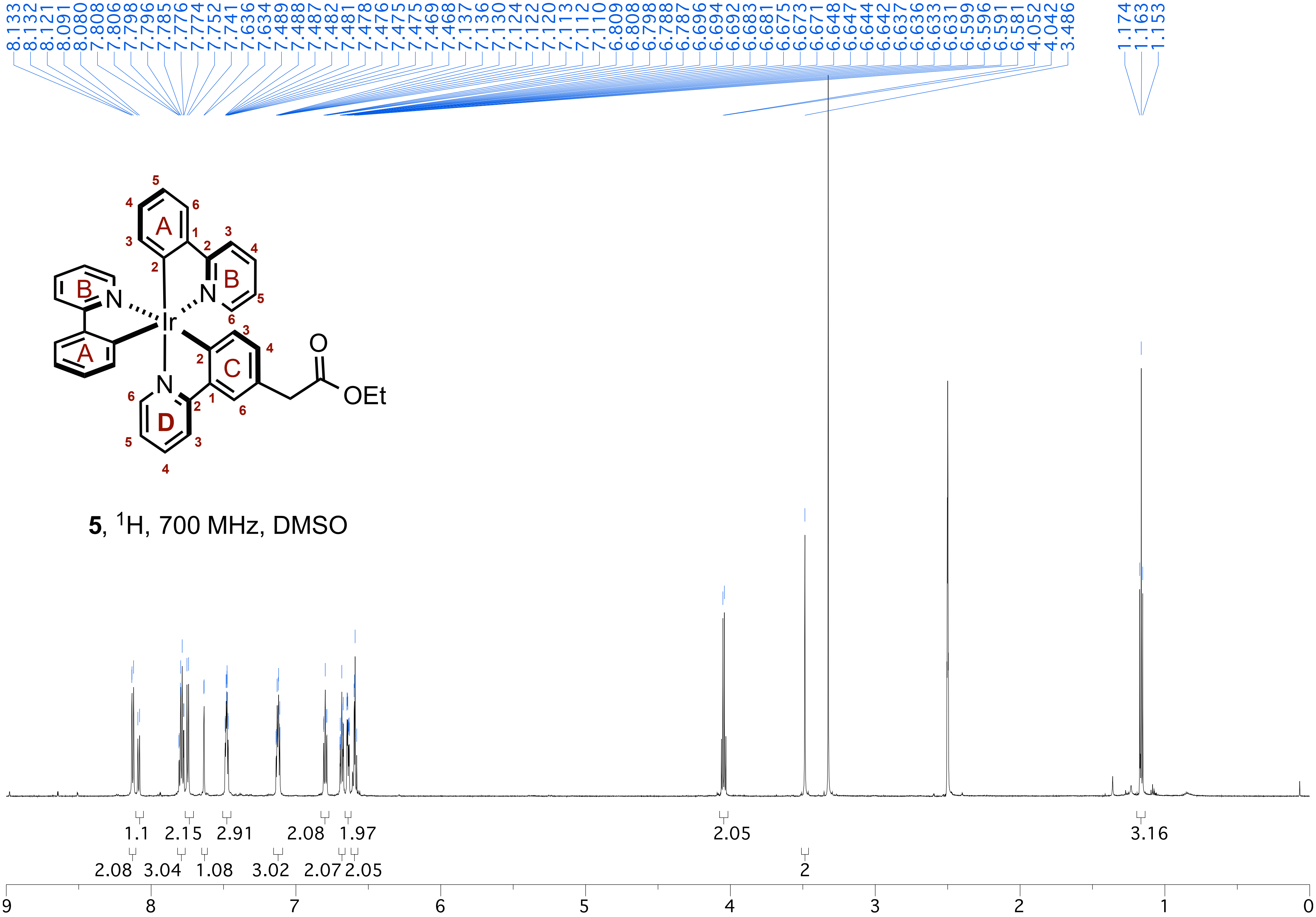

5, ^{1}H, 700 MHz, DMSO

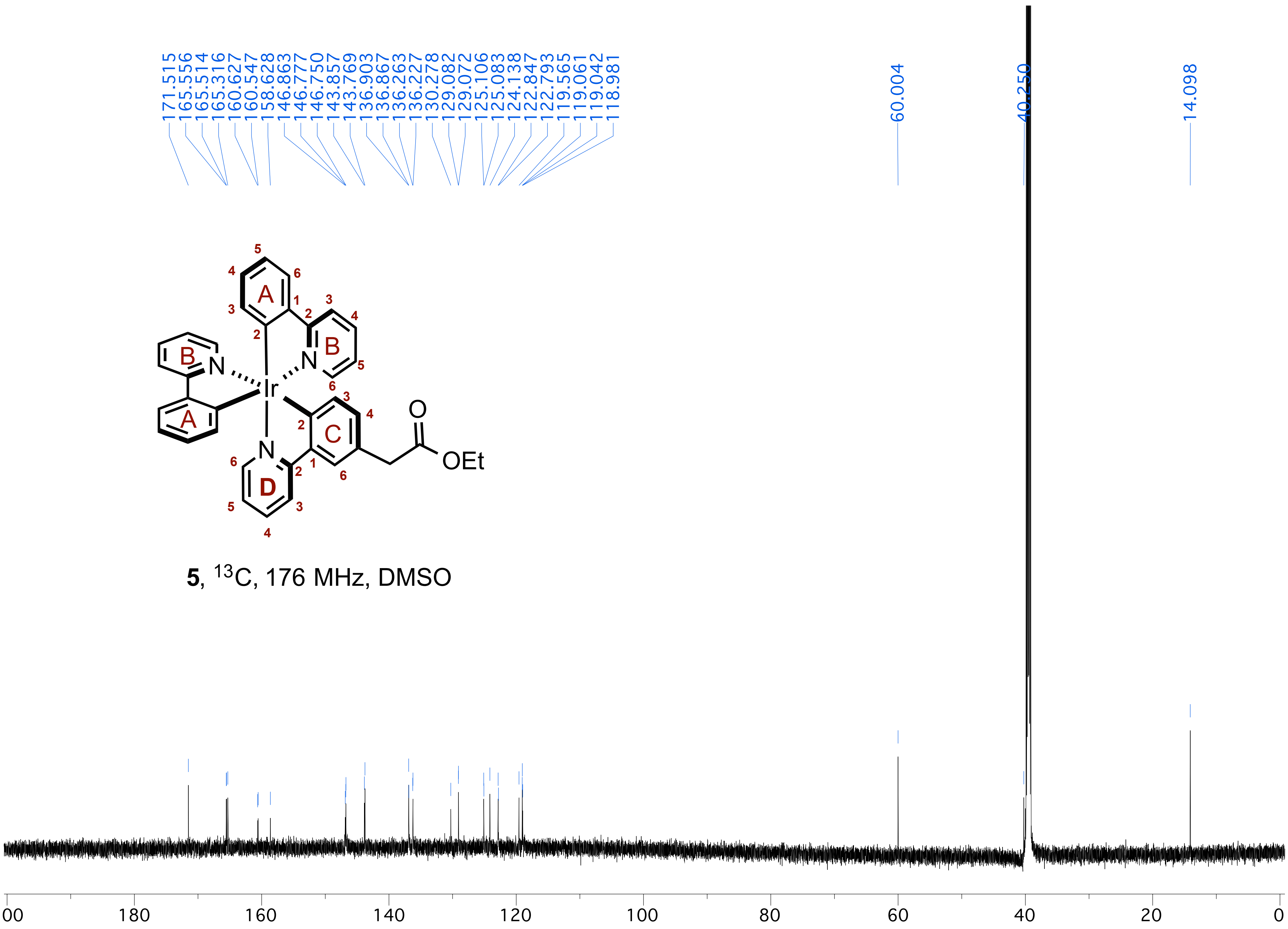

5, ^{13}C, 176 MHz, DMSO

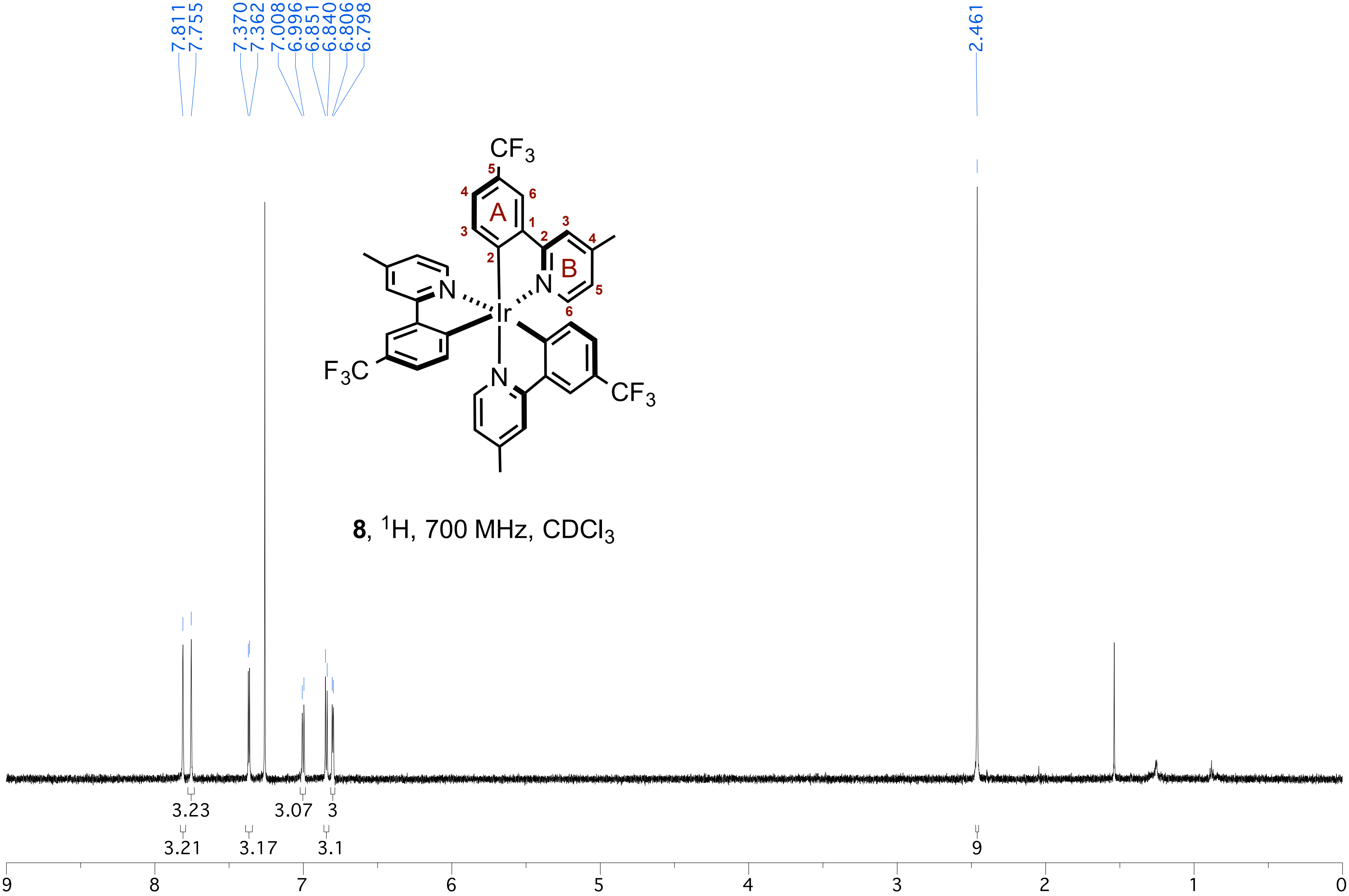

7.811
7.755
7.370
7.362
7.008
6.996
6.851
6.840
6.806
6.798
2.461
CF3
A
B
N
Ir
F3C
CF3
8, 1H, 700 MHz, CDCl3
3.23
3.07
3
3.21
3.17
3.1
9
9
8
7
6
5
4
3
2
1
0

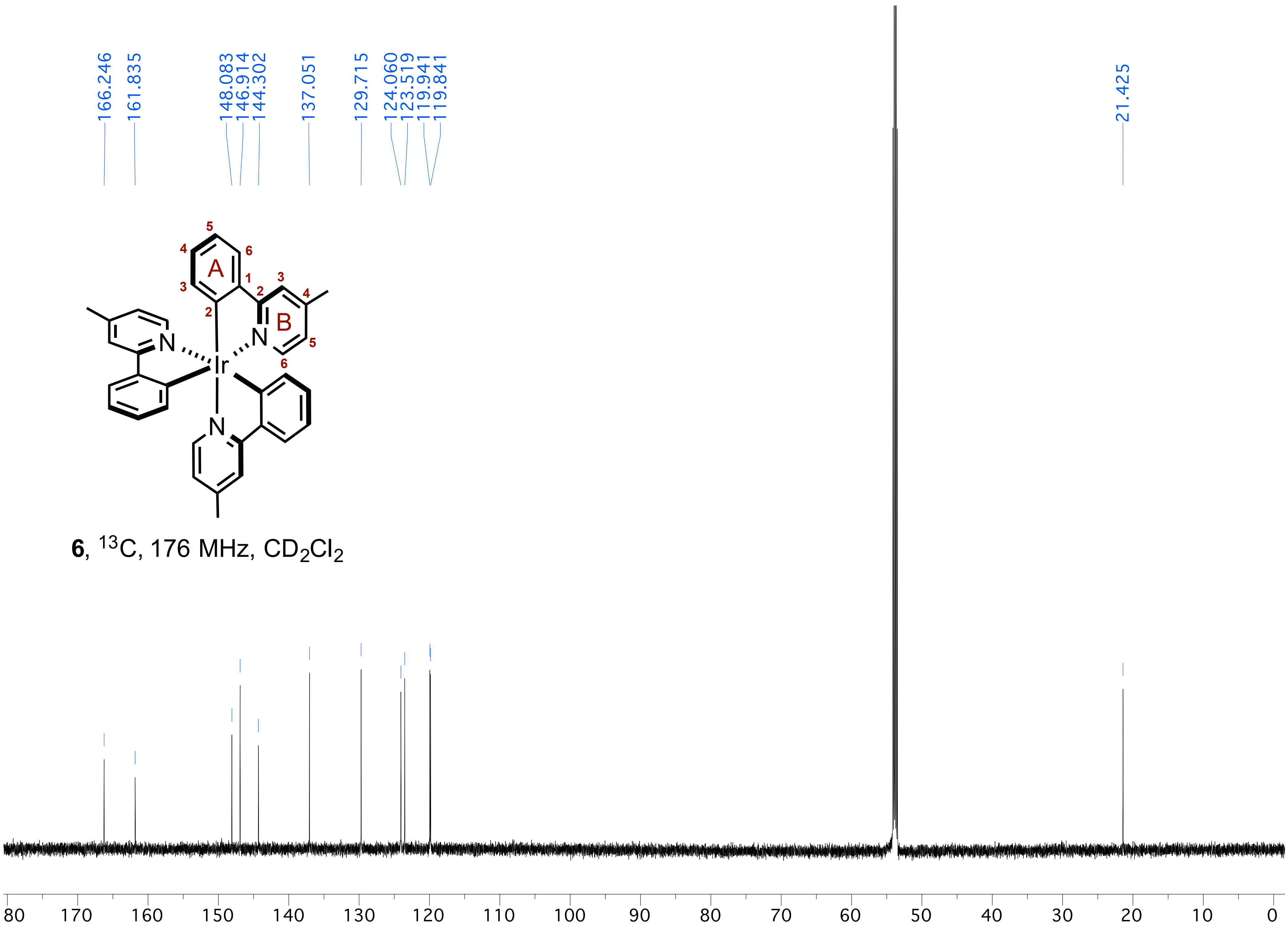

6, ^{13}C, 176 MHz, CD_2Cl_2

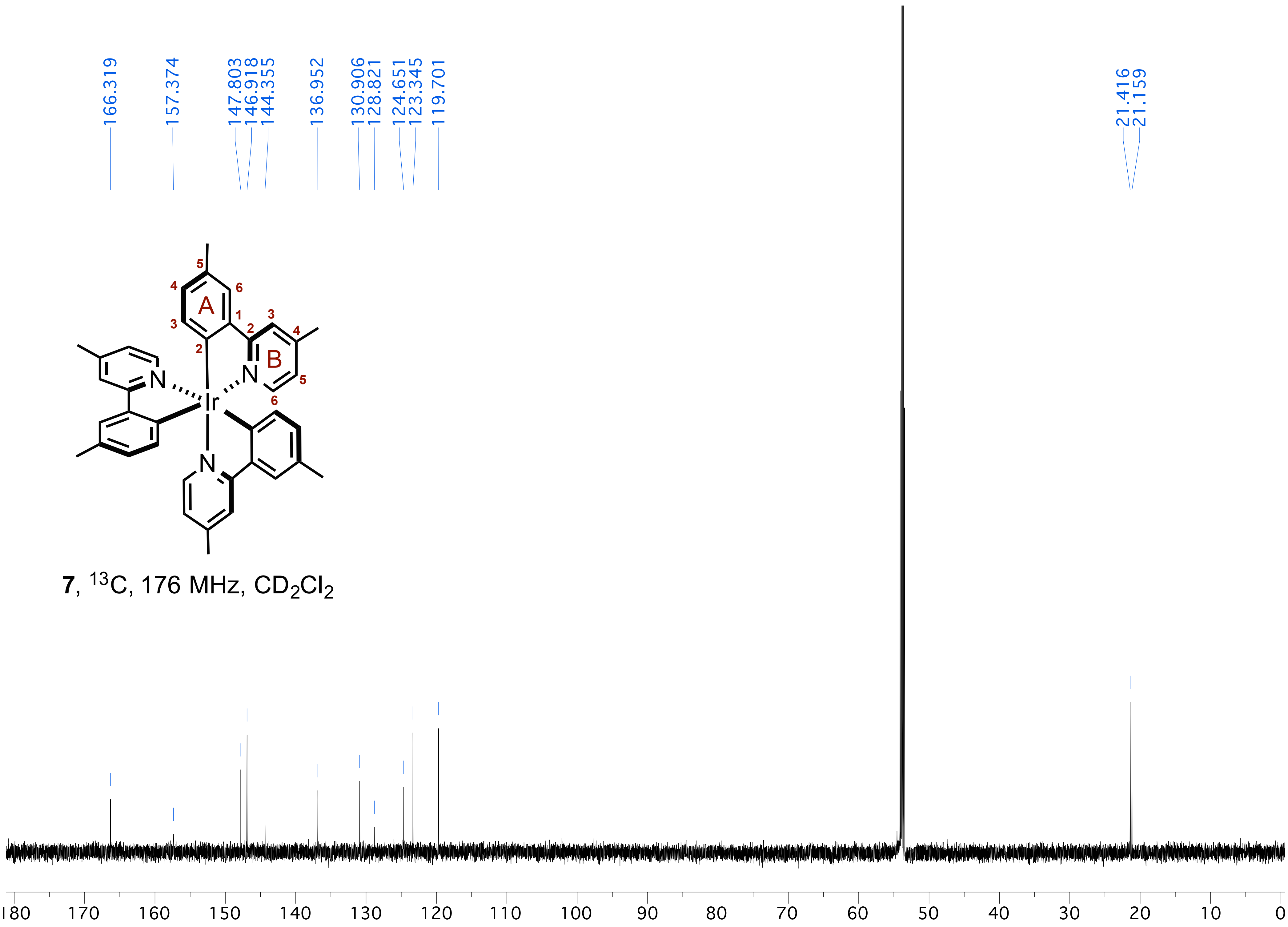
166.319
157.374
147.803
146.918
144.355
136.952
130.906
128.821
124.651
123.345
119.701
21.416
21.159
A
B
Ir
N
1
2
3
4
5
6
7, 13C, 176 MHz, CD2Cl2
180
170
160
150
140
130
120
110
100
90
80
70
60
50
40
30
20
10
0

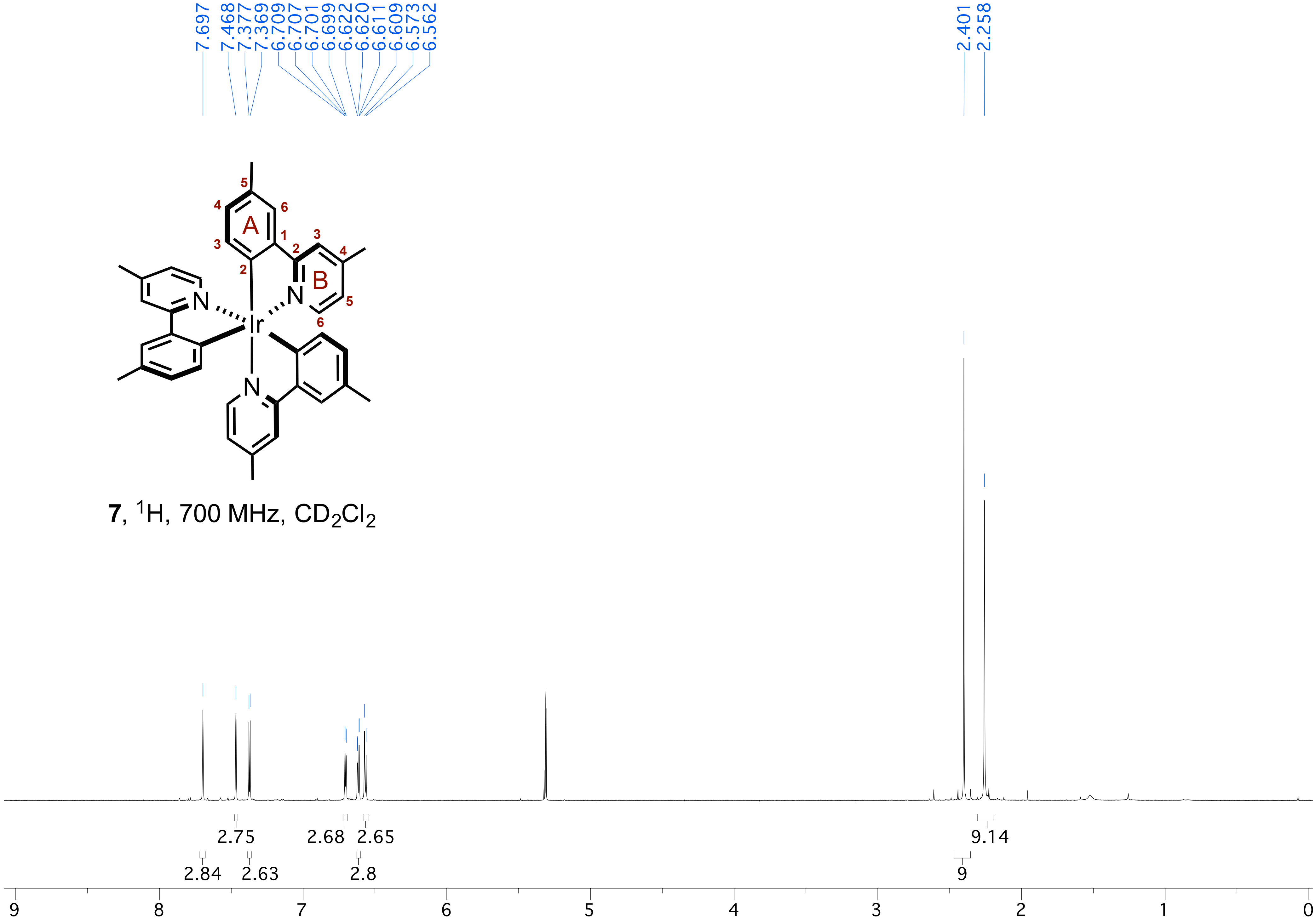
7.697
7.468
7.377
7.369
6.709
6.707
6.701
6.699
6.622
6.620
6.611
6.609
6.573
6.562
2.401
2.258
A
B
Ir
N
7, ^{1}H, 700 MHz, CD_2Cl_2
2.75
2.68
2.65
9.14
2.84
2.63
2.8
9
9
8
7
6
5
4
3
2
1
0

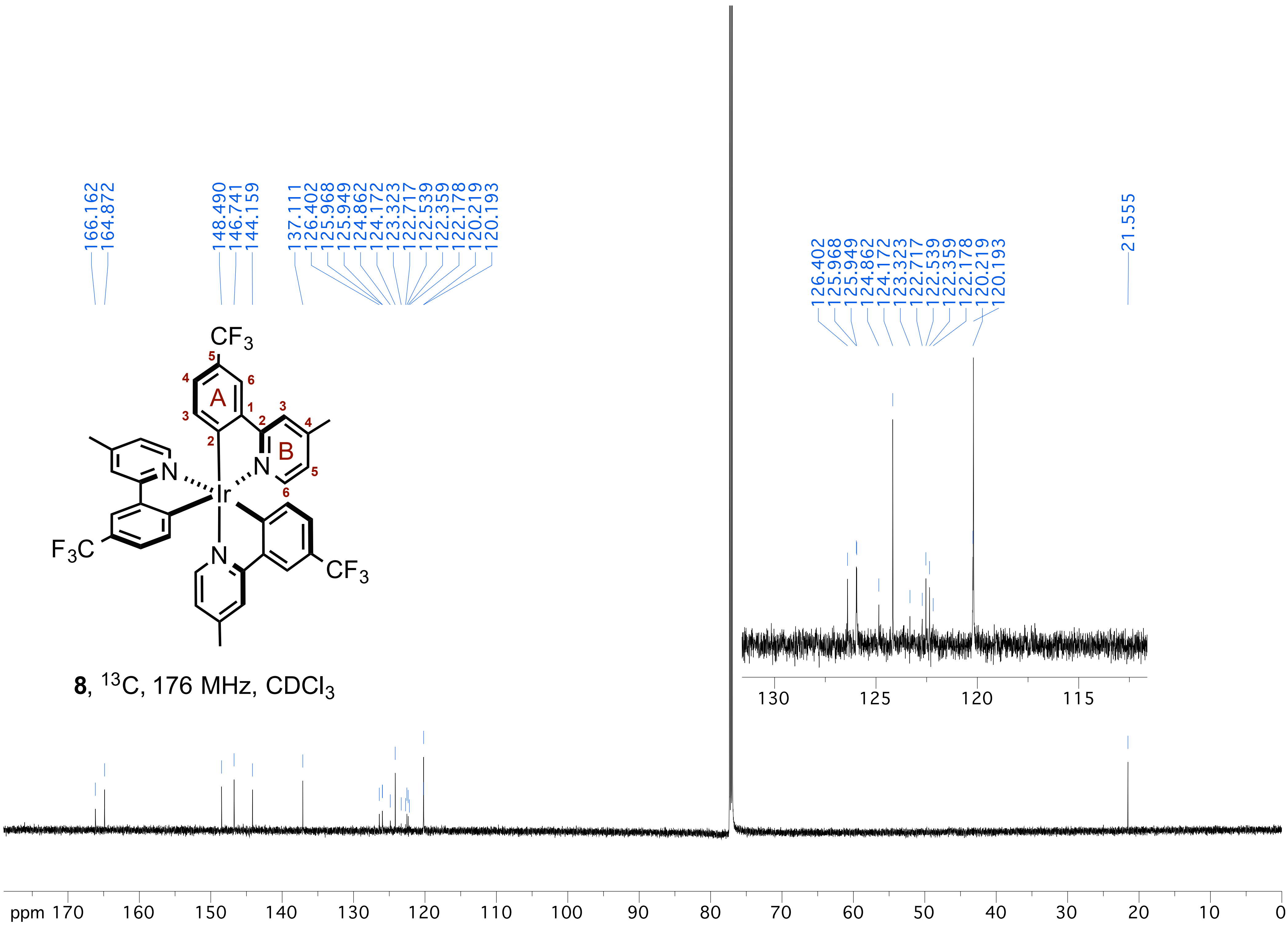
166.162
164.872
148.490
146.741
144.159
137.111
126.402
125.968
125.949
124.862
124.172
123.323
122.717
122.539
122.359
122.178
120.219
120.193
21.555
CF3
A
B
N
Ir
F3C
8, 13C, 176 MHz, CDCl3
130
125
120
115
ppm 170
160
150
140
130
120
110
100
90
80
70
60
50
40
30
20
10
0

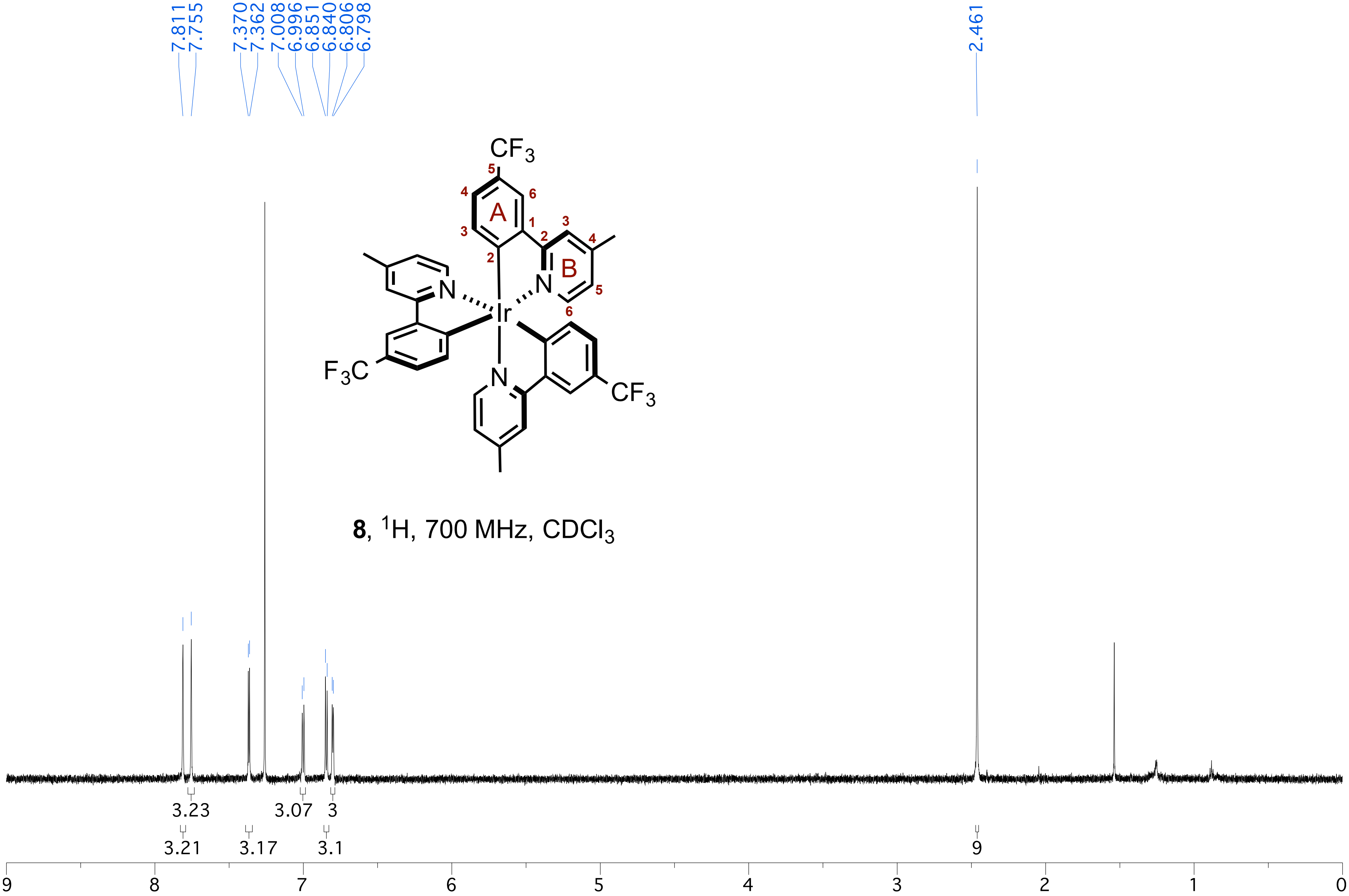
7.811
7.755
7.370
7.362
7.008
6.996
6.851
6.840
6.806
6.798
2.461
CF3
A
B
N
Ir
F3C
CF3
8, 1H, 700 MHz, CDCl3
3.23
3.07
3
3.21
3.17
3.1
9
9
8
7
6
5
4
3
2
1
0

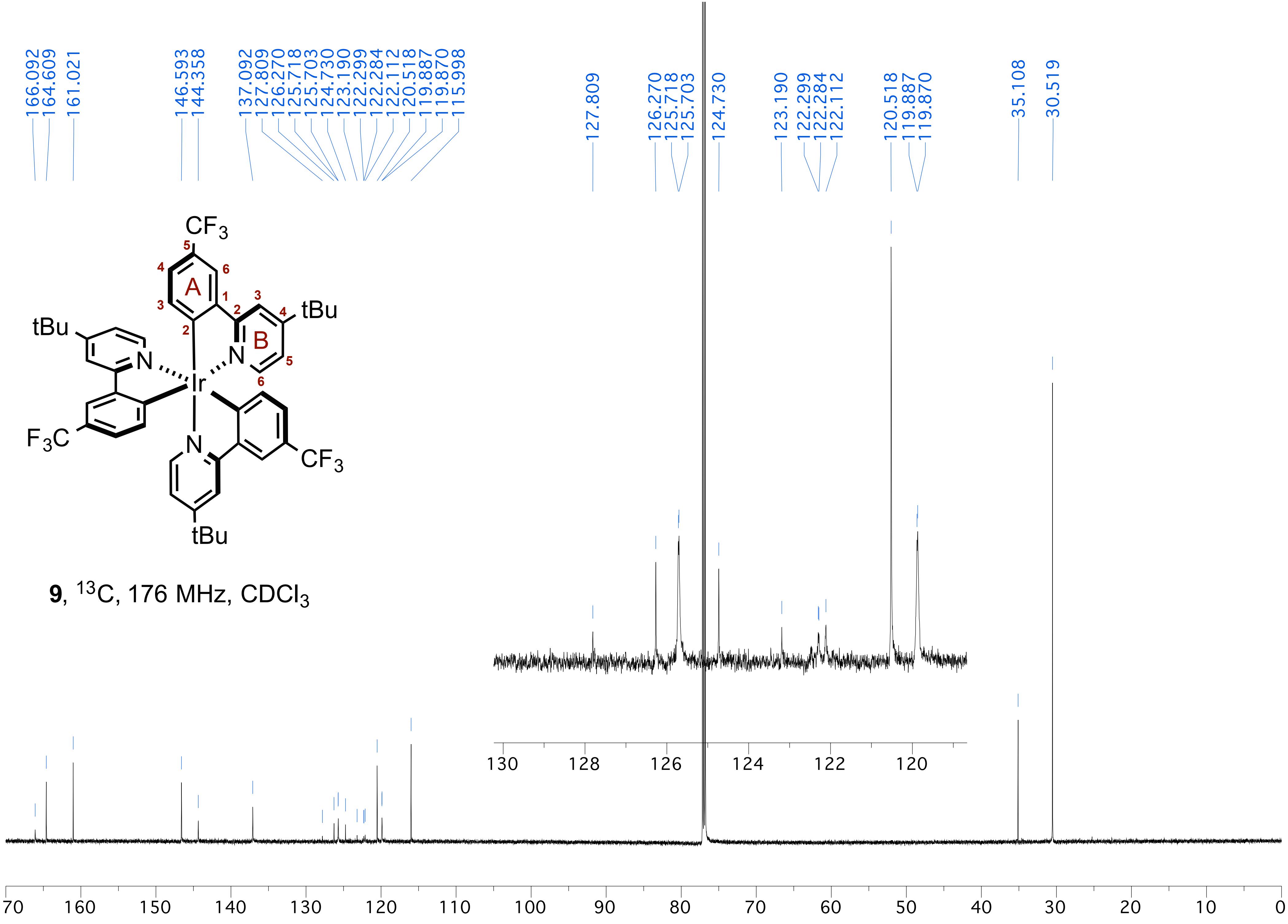

9, ^{13}C, 176 MHz, $CDCl_3$

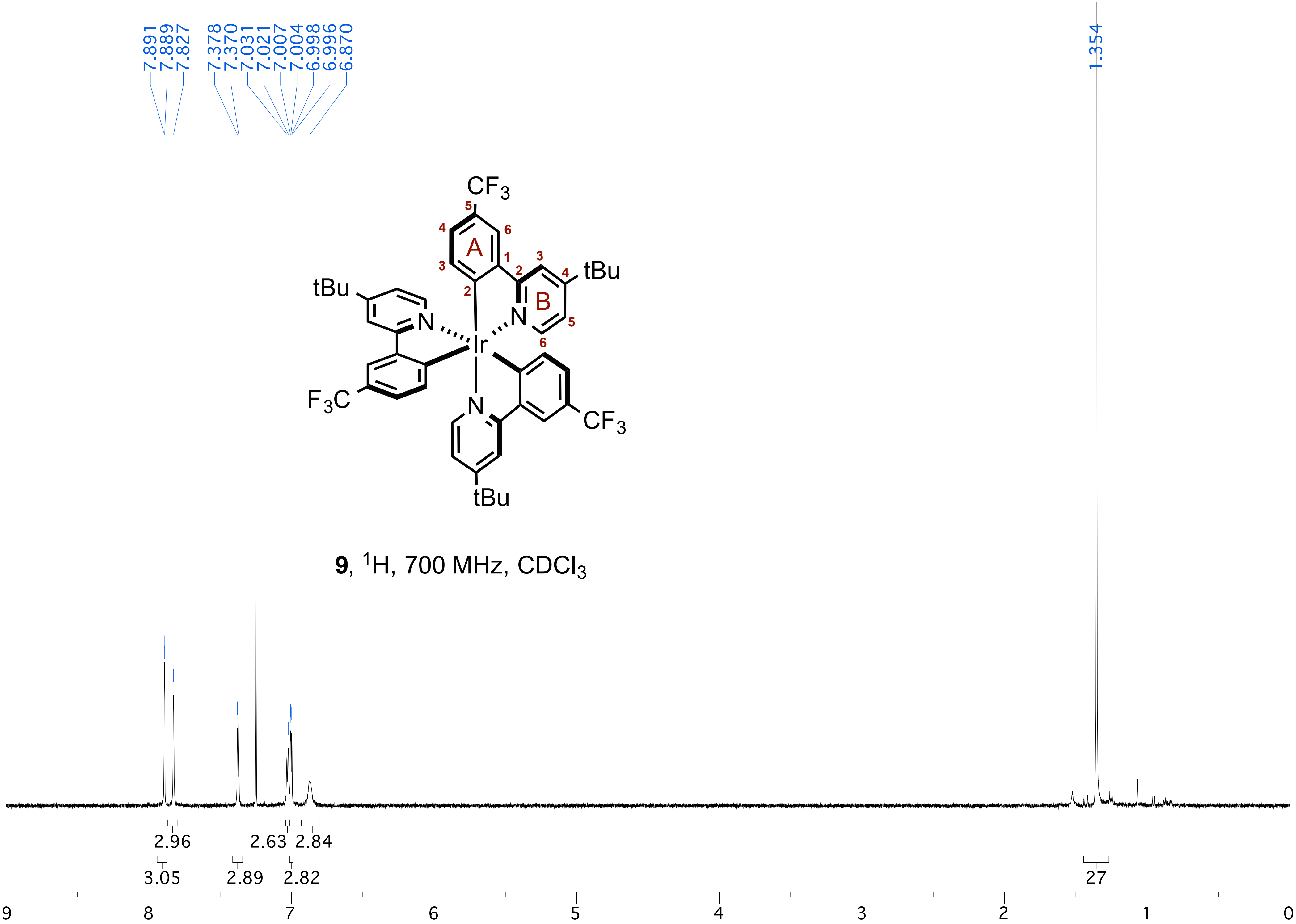

9, ^{1}H, 700 MHz, $CDCl_3$

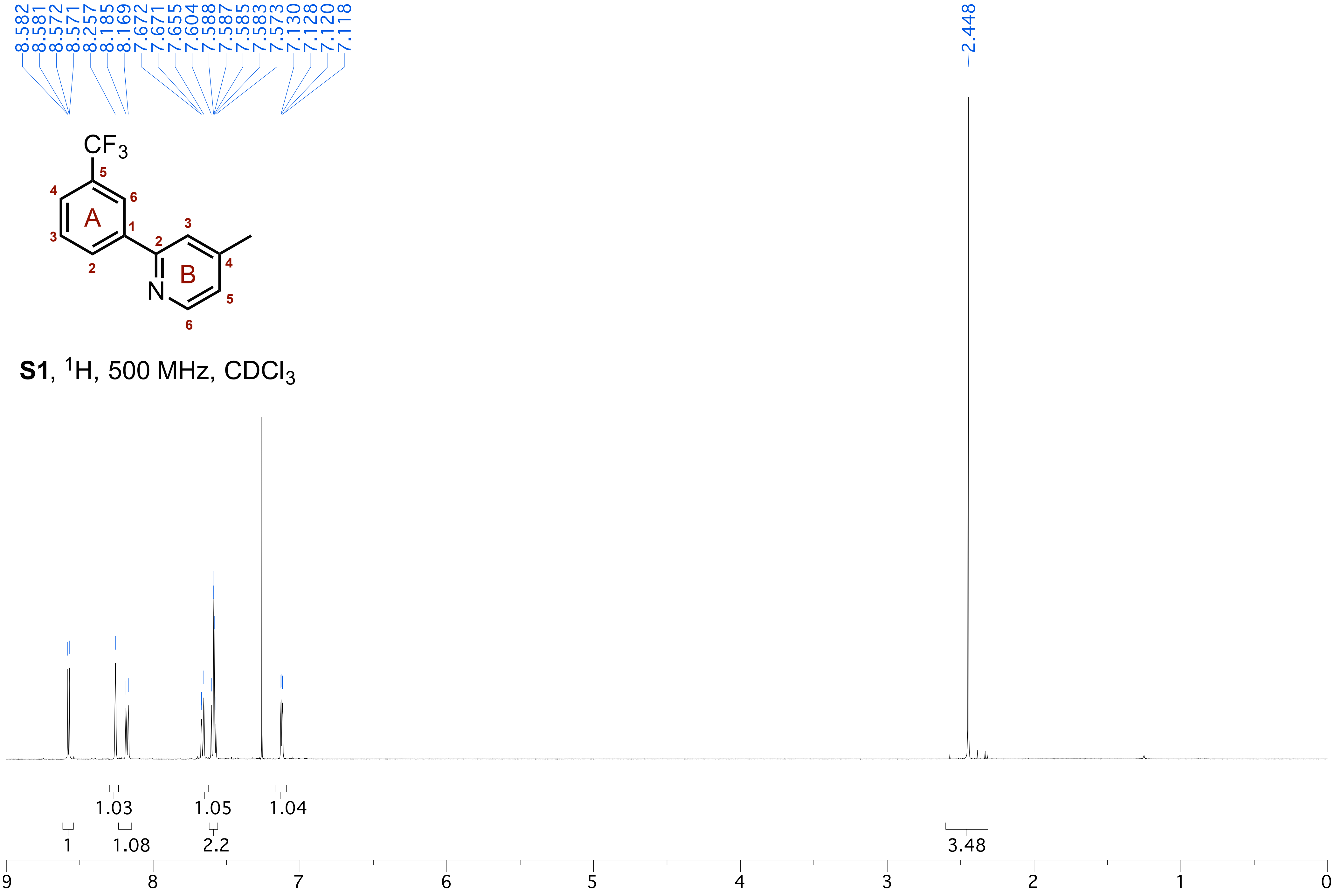
8.582
8.581
8.572
8.571
8.257
8.185
8.169
7.672
7.671
7.655
7.604
7.588
7.587
7.585
7.583
7.573
7.130
7.128
7.120
7.118
2.448
CF3
A
B
N
S1, 1H, 500 MHz, CDCl3
1.03
1.05
1.04
1
1.08
2.2
3.48
9
8
7
6
5
4
3
2
1
0

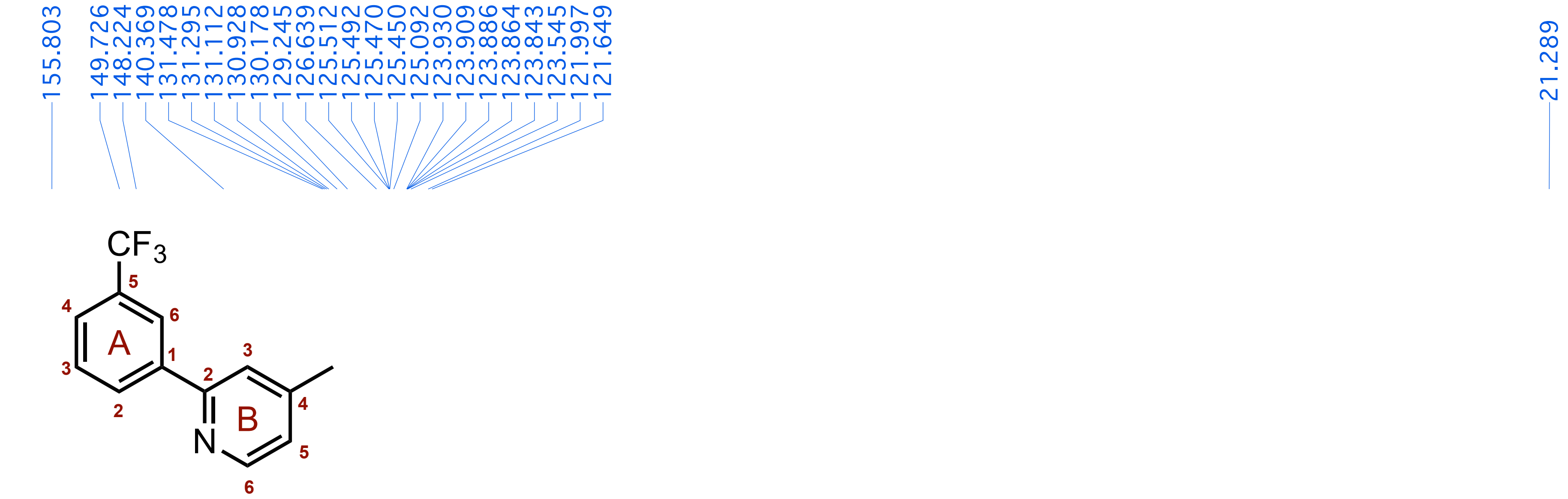

S1, ^{13}C, 176 MHz, $CDCl_3$

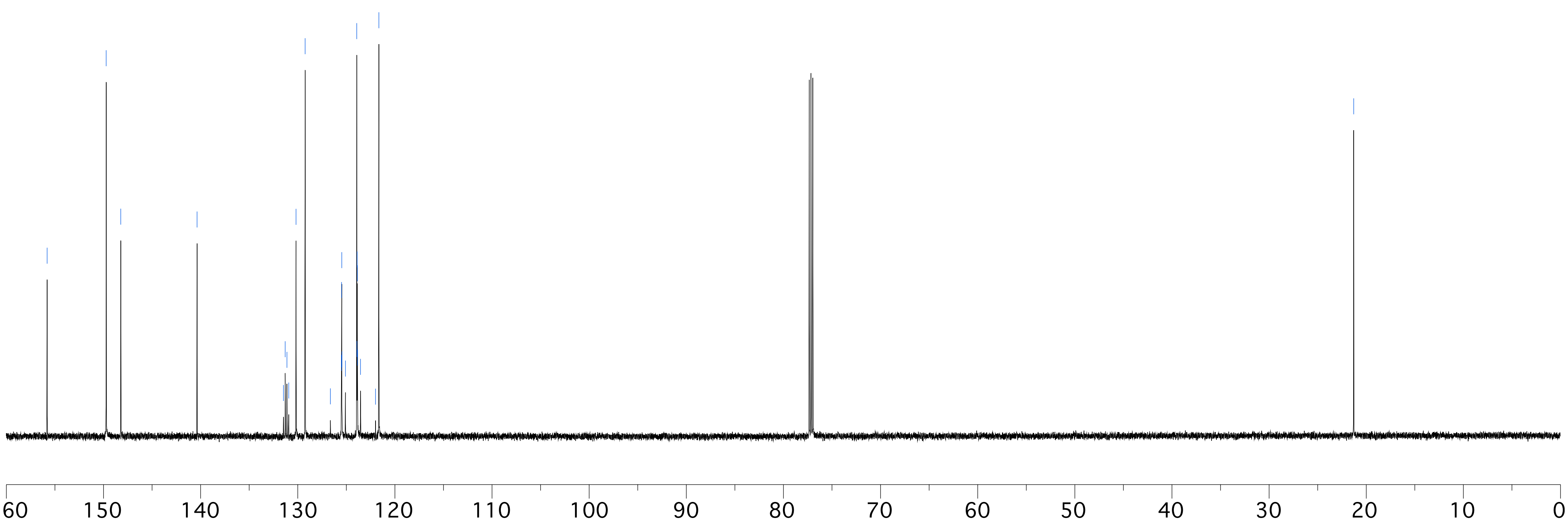

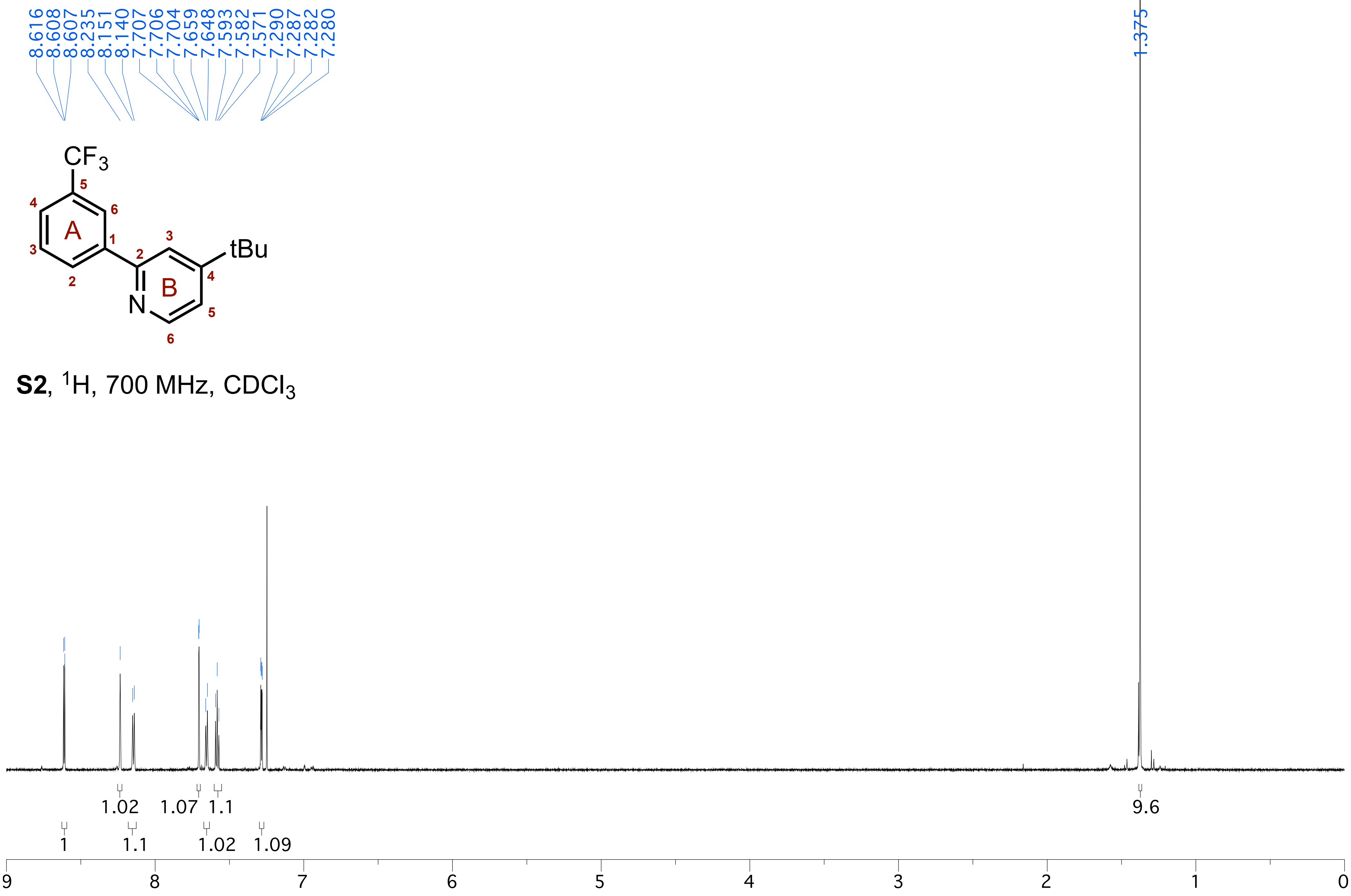

S2, ^{1}H, 700 MHz, $CDCl_3$

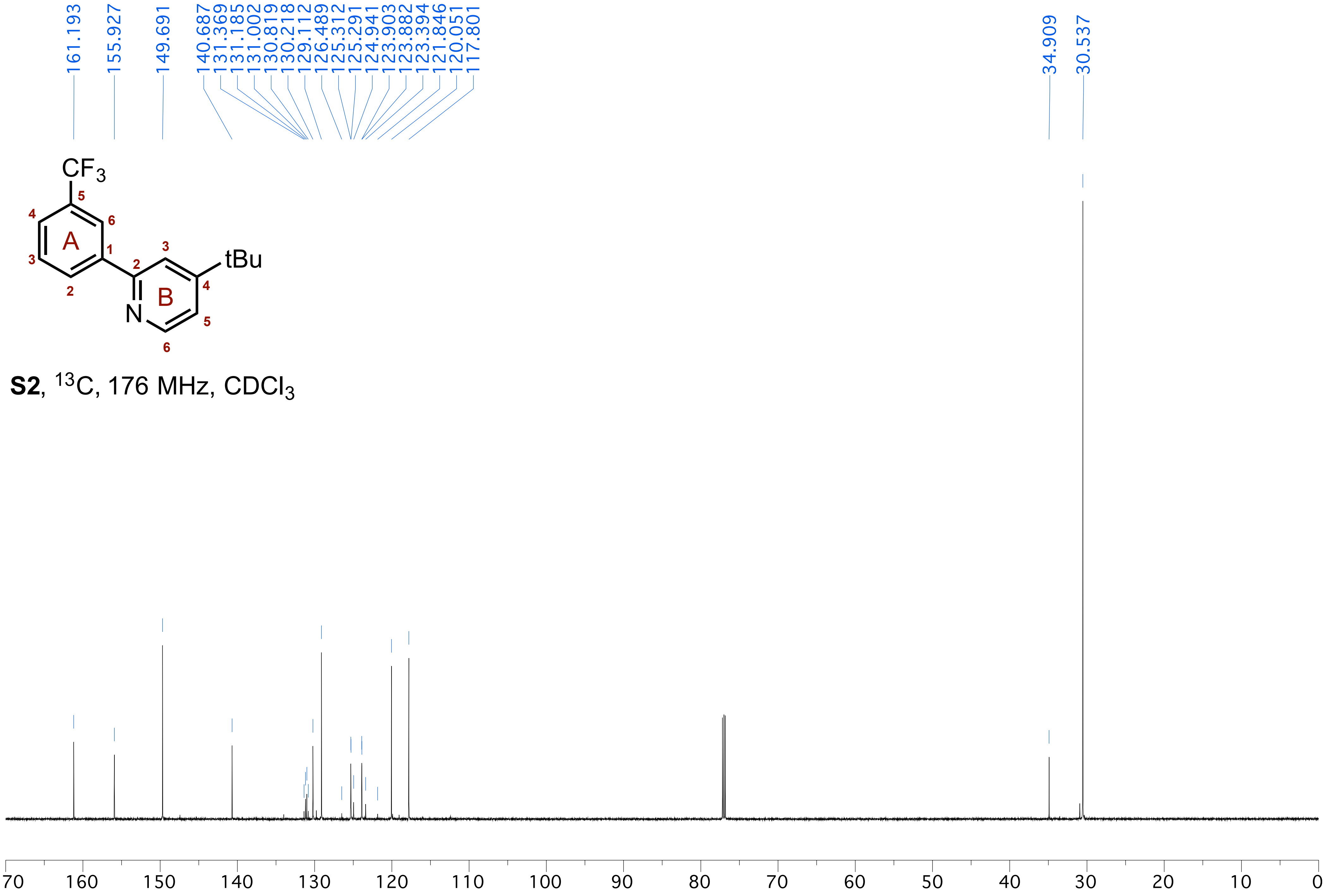

S2, ^{13}C, 176 MHz, $CDCl_3$

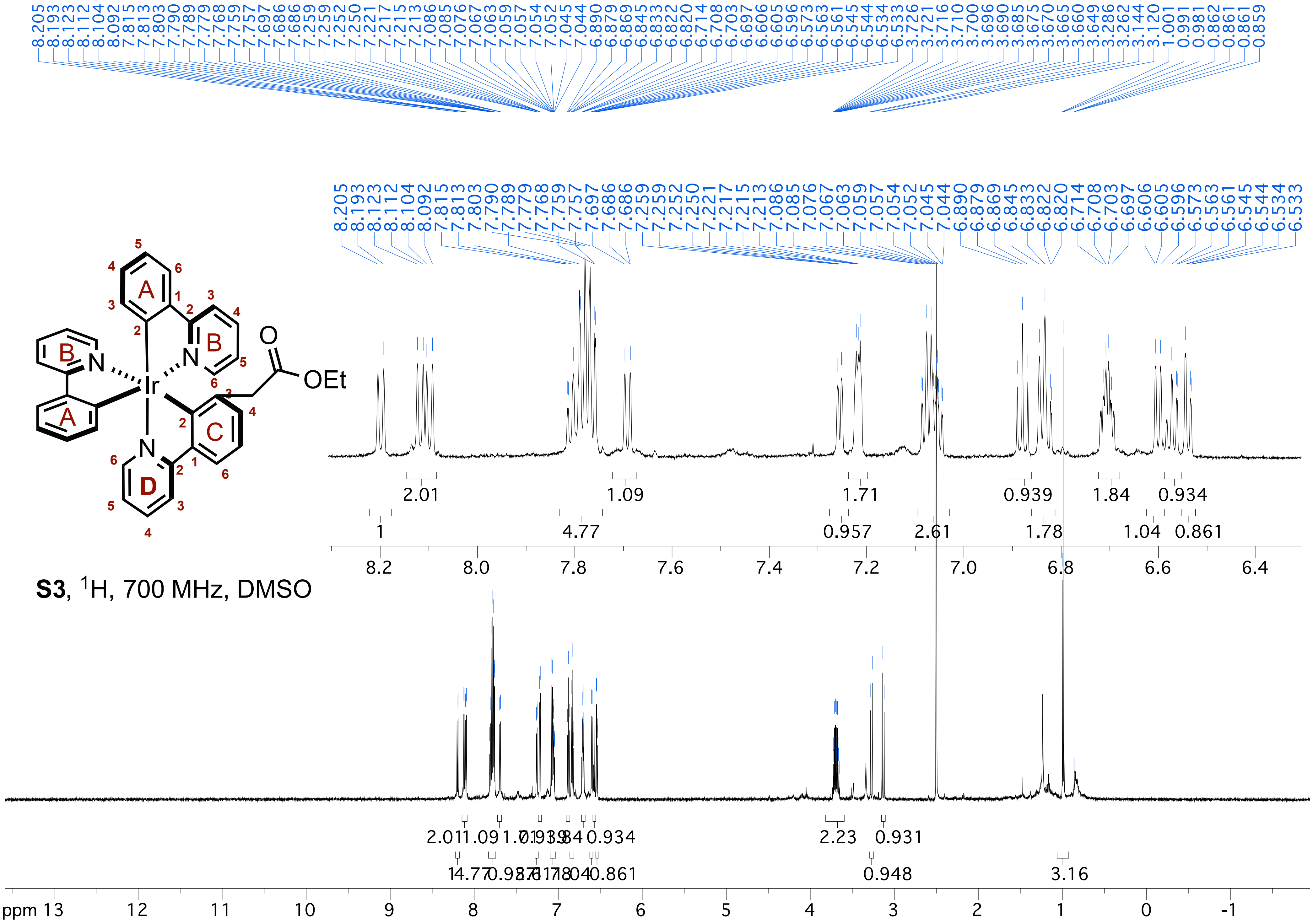

S3, ^1H, 700 MHz, DMSO

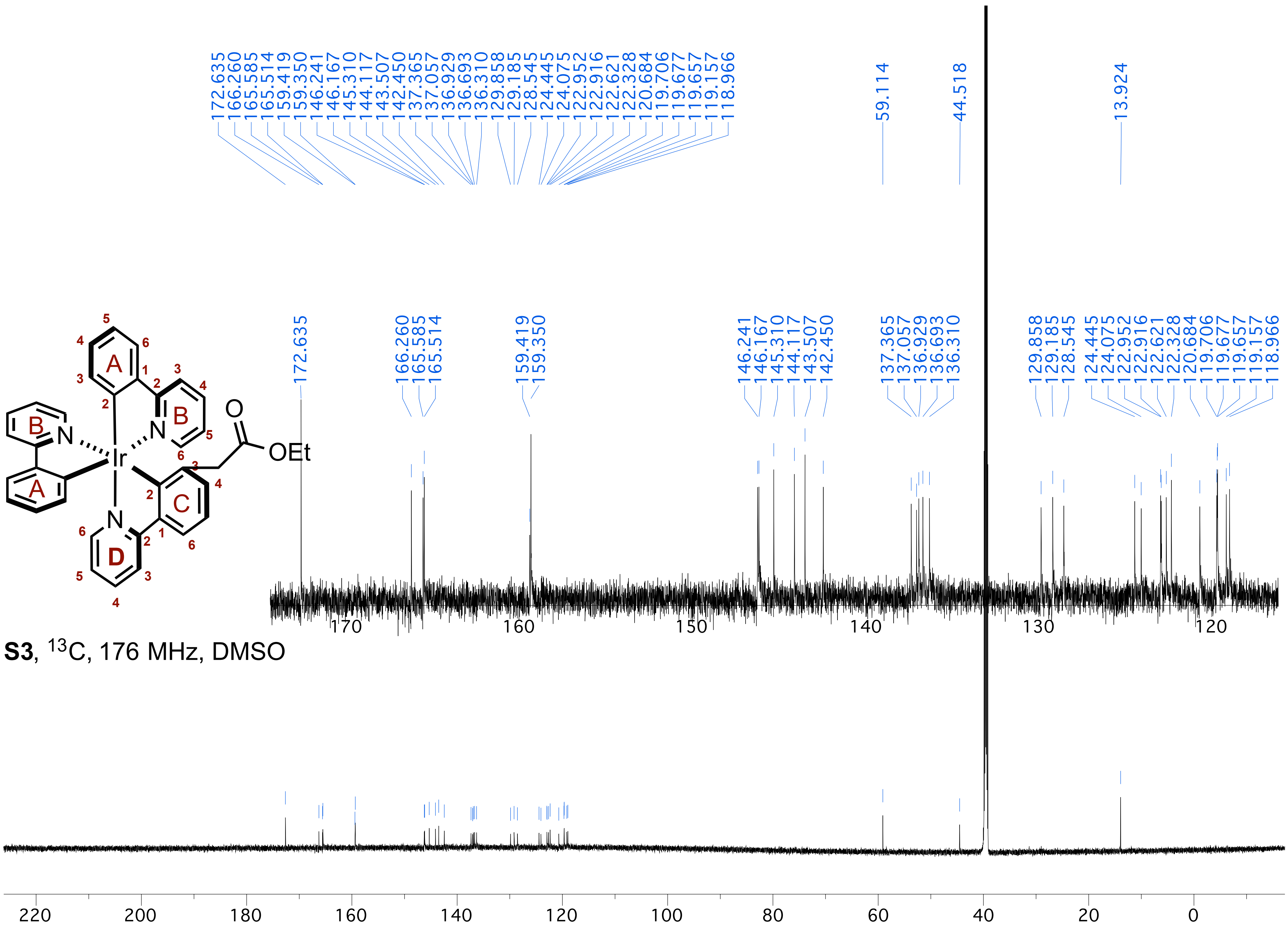

S3, ^{13}C, 176 MHz, DMSO

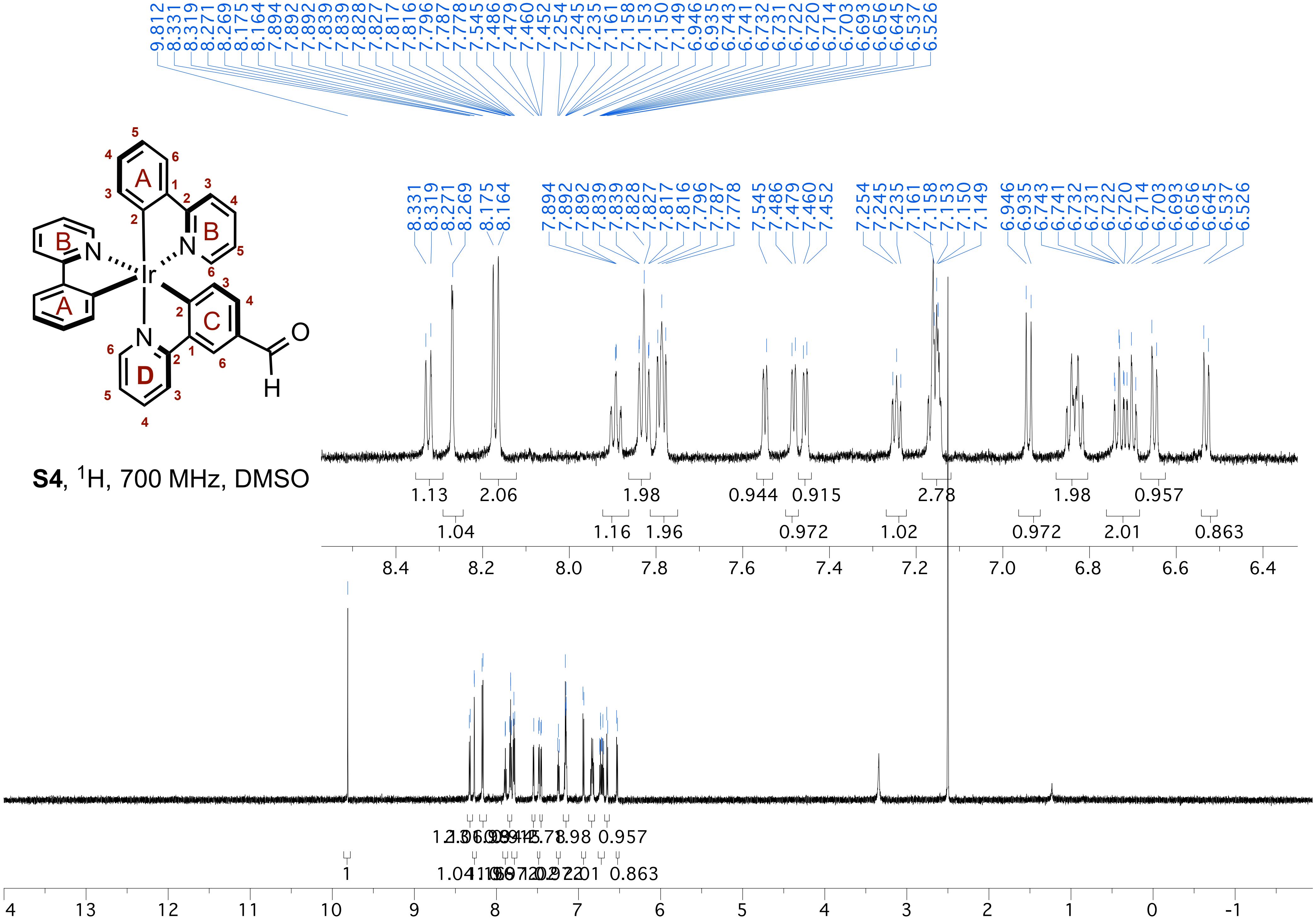

S4, ^{1}H, 700 MHz, DMSO

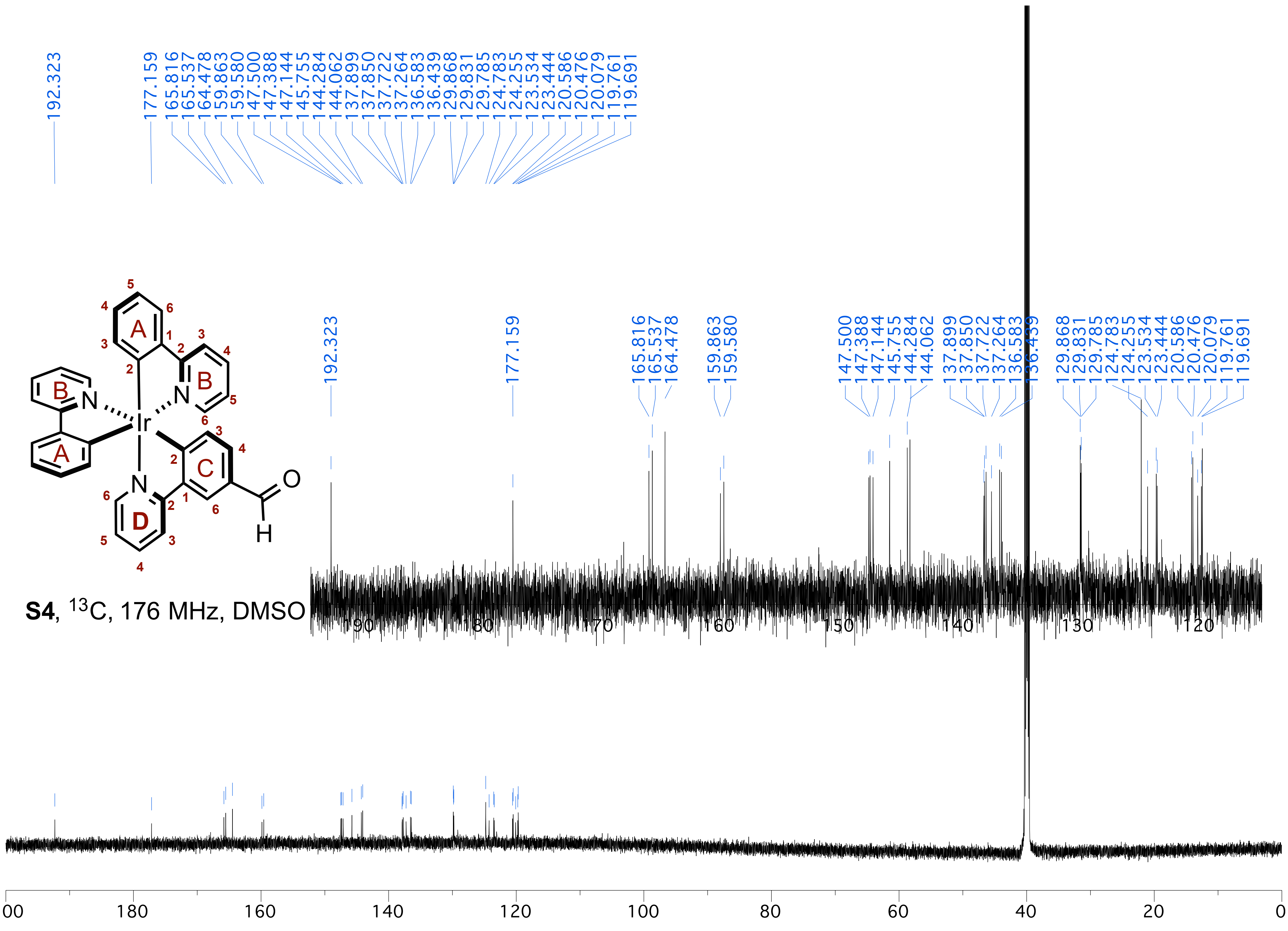

192.323
177.159
165.816
165.537
164.478
159.863
159.580
147.500
147.388
147.144
145.755
144.284
144.062
137.899
137.850
137.722
137.264
136.583
136.439
129.868
129.831
129.785
124.783
124.255
123.534
123.444
120.586
120.476
120.079
119.761
119.691
S4, 13C, 176 MHz, DMSO

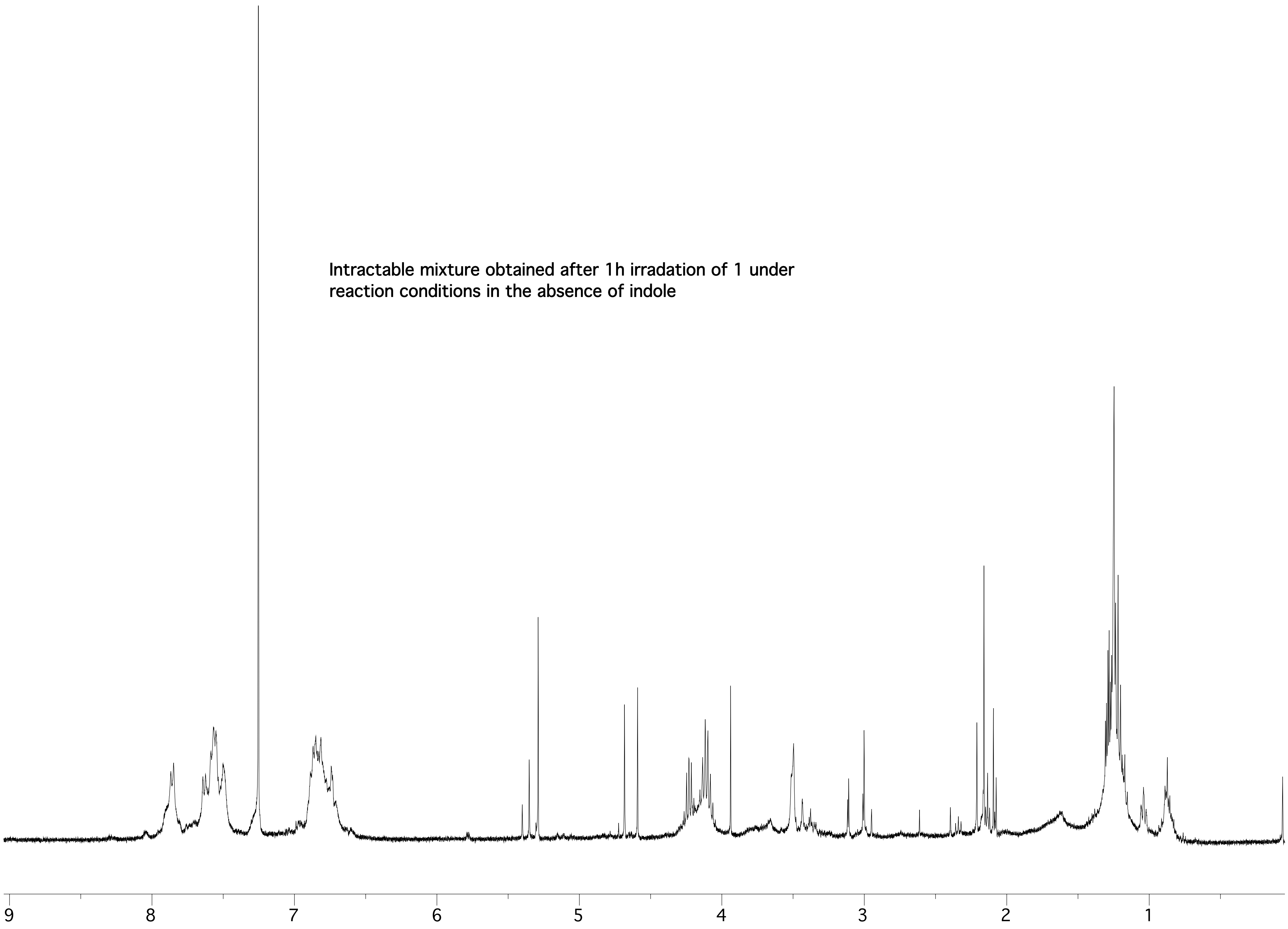
Intractable mixture obtained after 1h irradation of 1 under
reaction conditions in the absence of indole
9
8
7
6
5
4
3
2
1
